# Evidence and Potential Mechanisms of Jin-Gui Shen-Qi Wan as a Treatment for Type 2 Diabetes Mellitus: A Systematic Review and Meta-Analysis

**DOI:** 10.3389/fphar.2021.699932

**Published:** 2021-09-06

**Authors:** Zhipeng Hu, Xiaoke Liu, Maoyi Yang

**Affiliations:** Hospital of Chengdu University of Traditional Chinese Medicine, Chengdu, China

**Keywords:** Jin-Gui Shen-Qi Wan, traditional Chinese medicine, type 2 diabetes mellitus, systematic review, meta-analysis

## Abstract

**Background:** Type 2 diabetes mellitus (T2DM) is a subtype of diabetes mellitus characterized by progressive dysfunction of β-cell insulin secretion and insulin resistance. *Jīn-Guì Shèn-Qì Wán* (JGSQW) has for many years been widely used in clinical practice as a treatment for T2DM. However, its effect remains unknown.

**Objectives:** This study aims to summarize the clinical evidence of the effect of JGSQW on glucose and lipid metabolism in T2DM and the potential mechanisms underlying this effect.

**Methods:** Six databases were searched without language or publication status restrictions. Data were extracted to a predefined template for synthesis.

**Results:** Fourteen studies with 1586 participants were included in this meta-analysis. All 14 studies were judged to be at high risk of bias. JGSQW is safe for T2DM patients. Pooled results indicated that combination treatment results in a reduction in glycated hemoglobin (HbA1c) (mean difference (MD) −0.49%; 95% CI −0.67 to −0.31), fasting blood glucose (FBG) (MD −0.84; 95% CI −1.19 to −0.49), and 2-hour postprandial glucose 2hBG (MD −1.38; 95% CI −1.60 to −1.16). No significant difference in glucose metabolism was observed between JGSQW and hypoglycemic agents. The available evidence was insufficient to determine the effects on lipid metabolism. Sensitivity analyses indicated that these results were robust.

**Conclusion:** By combining the available evidence, we found that JGSQW is safe for T2DM patients. Compared with hypoglycemic agents alone, combination treatment with JGSQW enhances the effect on glucose metabolism in patients with T2DM. We found no difference in the efficacy of JGSQW alone compared to hypoglycemic agents alone. In terms of lipid metabolism, the current evidence is insufficient and too inconsistent for us to draw firm conclusions, so further studies are needed.

## Introduction

Type 2 diabetes mellitus (T2DM) is a subtype of diabetes mellitus (DM) characterized by progressive dysfunction of β-cell insulin secretion and insulin resistance (2. Classification and Diagnosis of Diabetes: Standards of Medical Care in Diabetes-2021 2021). Over 75% of patients with T2DM have abnormal lipid metabolism (Athyros et al., 2018; Eid et al., 2019) which contributes to the development of T2DM by increasing the insulin resistance. With more than 425 million diabetic patients globally, T2DM has become one of the most prevalent chronic diseases and the prevalence may increase with the rising numbers of obese patients (Cho et al., 2018; Li et al., 2020). In 2019, despite the fact that nearly 10% of global healthcare costs were related to diabetes management, 4.2 million adult deaths were attributed to diabetes and its complications (Peters et al., 2017; Association 2018; Dall et al., 2019). Although a series of hypoglycemic agents have been developed, an effective treatment of T2DM remains a challenge.

In China, botanical drugs are widely used in the treatment of T2DM (Chen et al., 2019; Teng et al., 2018)*. Jīn-Guì Shèn-Qì Wán* (JGSQW), first recorded in *Essentials from the Golden Cabinet* (*Jīn Guì Yào Lüè*), is a widely used traditional Chinese medicine (TCM) formula consisting of eight medicines: *Rehmannia glutinosa* (Gaertn.) DC. (*dì huáng*), Orobanchaceae; *Dioscorea* oppositifolia L. (*huái shān yào*), Dioscoreaceae; *Cornus officinalis* Siebold and Zucc. (*shān zhū yú*), Cornaceae; *Alisma plantago-aquatica* L. (*zé xiè*), Alismataceae; *Smilax glabra* Roxb. (*fú líng*), Smilacaceae; *Paeonia × suffruticosa* Andrews. (*mŭ dān pí*), Paeoniaceae; *Neolitsea cassia* (L.) Kosterm. (*guì zhī*), Lauraceae; and *Aconitum carmichaelii* Debeaux. (*zhì fù zĭ*), Ranunculaceae. The composition and all possible names of JGSQW are presented in Table 1. To date, there are no high-quality systematic reviews published on JGSQW as a treatment for T2DM. The experimental studies have indicated that JGSQW may exert its effect on T2DM through multiple mechanisms (Alam et al., 2014; Luo et al., 2015; Chen et al., 2016; Suchal et al., 2017; Zhuang et al., 2018; Yao et al., 2019). However, no studies to date have systematically summarized its beneficial effects and potential mechanisms.

**TABLE 1:** Composition and all possible names of JGSQW.

Pharmaceutical	Pīn yīn	Other names of JGSQW
*Rehmannia glutinosa* (Gaertn.) DC., Orobanchaceae	*dì huáng*	Shenqi Wan
*Dioscorea oppositifolia* L., Dioscoreaceae	huái shān yào	Shenqi Pill
*Cornus officinalis* Siebold and Zucc., Cornaceae	shān zhū yú	Dihuang Wan
*Alisma plantago-aquatica* L., Alismataceae	*zé xiè*	Dihuang Pill
*Smilax glabra* Roxb., Smilacaceae	*fú líng*	Jingui Shenqi Pill
*Paeonia × suffruticosa* Andrews., Paeoniaceae	*mŭ dān pí*	Jingui shenqi Wan
*Neolitsea cassia* (L.) Kosterm., Lauraceae	*guì zhī*	Guifu Dihuang Wan
*Aconitum carmichaelii* Debeaux., Ranunculaceae	*zhì fù zĭ*	Guifu Dihuang Pill

Given this situation, the aim of this study is to 1) conduct a systematic review and meta-analysis of the clinical evidence of the effect of JGSQW on glucose and lipid metabolism in T2DM and 2) summarize the beneficial effects and potential mechanisms of JGSQW on T2DM as demonstrated in the experimental studies. In this study, the herb–drug interaction is analyzed through careful examination of the clinical and preclinical evidence. This study presents an accurate update on this issue as a basis for future research and clinical practice.

## Materials and Methods

To optimize our approach, this systematic review was conducted and reported following the guidelines provided by the Cochrane Handbook for Systematic Reviews of Interventions version 6.0 (updated July 2019), methodological expectations for conduct, reporting and updating of systematic reviews of intervention (MECIR), the Preferred Reporting Items for Systematic Reviews and Meta-Analyses (PRISMA) 2020 statement, and A Measurement Tool to Assess Systematic Reviews 2 (AMSTAR 2) (Shea et al., 2017; Page et al., 2021). The PRISMA 2020 checklist is provided in Supplementary Material S1. As demonstrated by the AMSTAR 2 assessment in Supplementary Material S2, the overall methodological quality of this meta-analysis is high.

### Search Strategies

Three English language databases (PubMed, Embase, and the Cochrane Central Register of Controlled Trials (CENTRAL)) and three Chinese language databases (China National Knowledge Infrastructure (CNKI), Wanfang Data Knowledge Service Platform, and VIP information resource integration service platform (cqvip)) were searched from inception to February 1, 2021, without language or publication status restrictions. The ClinicalTrials.gov database and Chinese Clinical Trial Registry (CHiCTR) were also searched to find any ongoing or unpublished clinical trials. In addition, the reference lists of the reviews and meta-analyses on this topic were searched. All the names of JGSQW are interchangeable and were used in database searching. The detailed search strategies are provided in Supplementary Material S3.

### Inclusion and Exclusion Criteria for Meta-Analysis

#### Study Types

Randomized controlled trials (RCTs) were included. Observational studies were not included due to the high risk of bias and confounding factors.

#### Participants

Studies including adults (18 years or older) with an established diagnosis of T2DM or T2DM complications were included. No restrictions were placed on other demographic factors of participants and settings. Studies including only a subset of relevant participants were included and the sensitivity analysis was carried out to assess the impact of including these studies.

#### Interventions

In TCM, subtle changes in formula composition can lead to significant differences in indications. According to the basic principles of evidence-based medicine, different interventions cannot be combined in the same meta-analysis. The composition of JGSQW in the included studies must be the eight medicines mentioned above without modification. The medicine was taken orally. Ideally, the authors should report the composition of the medicine they used including the source of any Chinese patent medicine. Studies missing this information were included in the primary analysis and excluded from the sensitivity analysis. Studies using modified JGSQW were not included in this meta-analysis. Comparators could include a placebo, anti-diabetic agents, and lifestyle management. Co-interventions, if administered to the intervention group, should also apply to the control group.

#### Comparisons

The following comparisons were investigated separately in this research:

The first is JGSQW alone vs hypoglycemic agents, and the second is the combination treatment of JGSQW and hypoglycemic agents vs hypoglycemic agents.

In addition, we performed further comparisons according to the specific medicines used in the experimental and control groups.

#### Outcome Measures

The following outcome measures with established measurement methods were included:

Primary: glycated hemoglobin (HbA1c).

Secondary: fasting blood glucose (FBG), two-hour postprandial glucose (2hBG), high-density lipoprotein cholesterol (HDL-C), low-density lipoprotein cholesterol (LDL-C), total cholesterol (TC), and triglyceride (TG).

If multiple time points were reported, the results with the longest time point were included in the analysis.

#### Safety Outcome

Any adverse events during the trials can be included.

### Data Collection and Analysis

#### Study Selection

Two authors (Zhipeng Hu and Maoyi Yang) screened all the citations independently by reading the titles and abstracts. Studies meeting the inclusion criteria were obtained for further screening. Disagreements were resolved by discussion with the third author (Xiaoke Liu). Studies that could not be included for reasons such as incomplete information in the abstract were classified as “Studies Awaiting Classification.” Studies excluded after reading the full text and reasons for exclusion are listed in Supplementary Material S4.

#### Data Extraction

A form was developed prior to data collection to record the following data about each study: first author and year, country, diagnostic criteria, participant age (treatment/control; years), number of participants (treatment/control), number of females (treatment/control), duration of T2DM (treatment/control; years), co-intervention, treatment, comparator, duration of treatment, funding, and outcomes. Data extraction was carried out by two authors independently (Zhipeng Hu and Maoyi Yang), and the discrepancies were resolved by discussion with the third author (Xiaoke Liu). We contacted study authors by email for more unpublished information if necessary. Transformation of reported data was not carried out in this study.

#### Risk of Bias Assessment

A risk of bias assessment was carried out using the 2019 Cochrane risk-of-bias tool (version 2) for randomized trials (RoB2) (Sterne et al., 2019). This risk of bias assessment includes the following five domains: bias arising from the randomization process, bias due to deviations from intended interventions, bias due to missing outcome data, bias in measurement of the outcome, and bias in selection of the reported result—as well as an overall risk-of-bias judgement. The nature of the effect of interest was an “intention-to-treat” effect. Discrepancies were resolved by discussion with the third author (Xiaoke Liu). To ensure transparency of assessment, judgments and agreement on these are provided in Supplementary Material 5.

#### Data Synthesis

Data analysis was carried out in accordance with the *Cochrane Handbook for Systematic Reviews of Interventions* version 6.0 (updated August 2019). Meta package (version 4.11–0) for RStudio (Version 1.2.1335, https://www.rstudio.com/) was used for data synthesis. Forest plots were used to present the results. mean difference (MD) with 95% confidence intervals (CI) were used as effect measures for continuous outcomes. No dichotomous outcomes were used in this study. Prediction intervals were calculated to indicate the extent of between-study variation if the number of included studies was more than ten. Considering the likelihood of heterogeneity, a random-effects model was used to pool the studies and a fixed-effects model was used for the sensitivity analysis.

#### Heterogeneity and Subgroup Analyses

A chi-squared test was used to test the heterogeneity between the studies with a significance level of *P* < 0.1. *I* ^*2*^ statistics were used to quantify the heterogeneity with values of 0–40%, 30–60%, and 75–100% indicating low, moderate, and high heterogeneity, respectively. The following potential effect modifiers were considered when conducting subgroup analyses:

Baseline HbA1c (> 7% or ≤7%), baseline body mass index (BMI) (> 24 kg/m^2^ or ≤ 24 kg/m^2^), comorbidity (yes or no), age (≥ 65 years old or <65 years old), and form of JGSQW (patent or decoction).

The following additional subgroup analysis was performed for FBG: level of control group (≥ 9 mmol/L or < 9 mmol/L).

#### Assessing Non-Reporting Biases

We evaluated the non-reporting biases by comparing the published literature and protocols or registration information if possible. The funnel plot and Egger’s test were carried out if at least 10 studies could be included in the analysis.

#### Dealing with Missing Data

We contacted the corresponding authors for missing data. In the absence of a reply, we imputed replacement values.

### Identification of Phytochemical Profile, Herb–Drug Interactions With Anti-Diabetic Drugs, and Potential Mechanisms

The phytochemical profile of eight herbal medicines and JGSQW was identified in the platform of the TCM System Pharmacology (TCMSP) database (http://lsp.nwu.edu.cn/tcmsp.php) with two screening criteria: oral bioavailability (OB) ≥ 30% and drug-likeness (DL) ≥ 0.18.

The preclinical studies are an important source for understanding the beneficial effects and herb–drug interactions. In order to provide a more comprehensive understanding of JGSQW as a treatment for T2DM, we identified all relevant preclinical studies to date and extracted data on herb–drug interactions between JGSQW and anti-diabetic drugs and on the potential mechanisms for tabulation.

## Results

### Database Search

**FIGURE 1:**
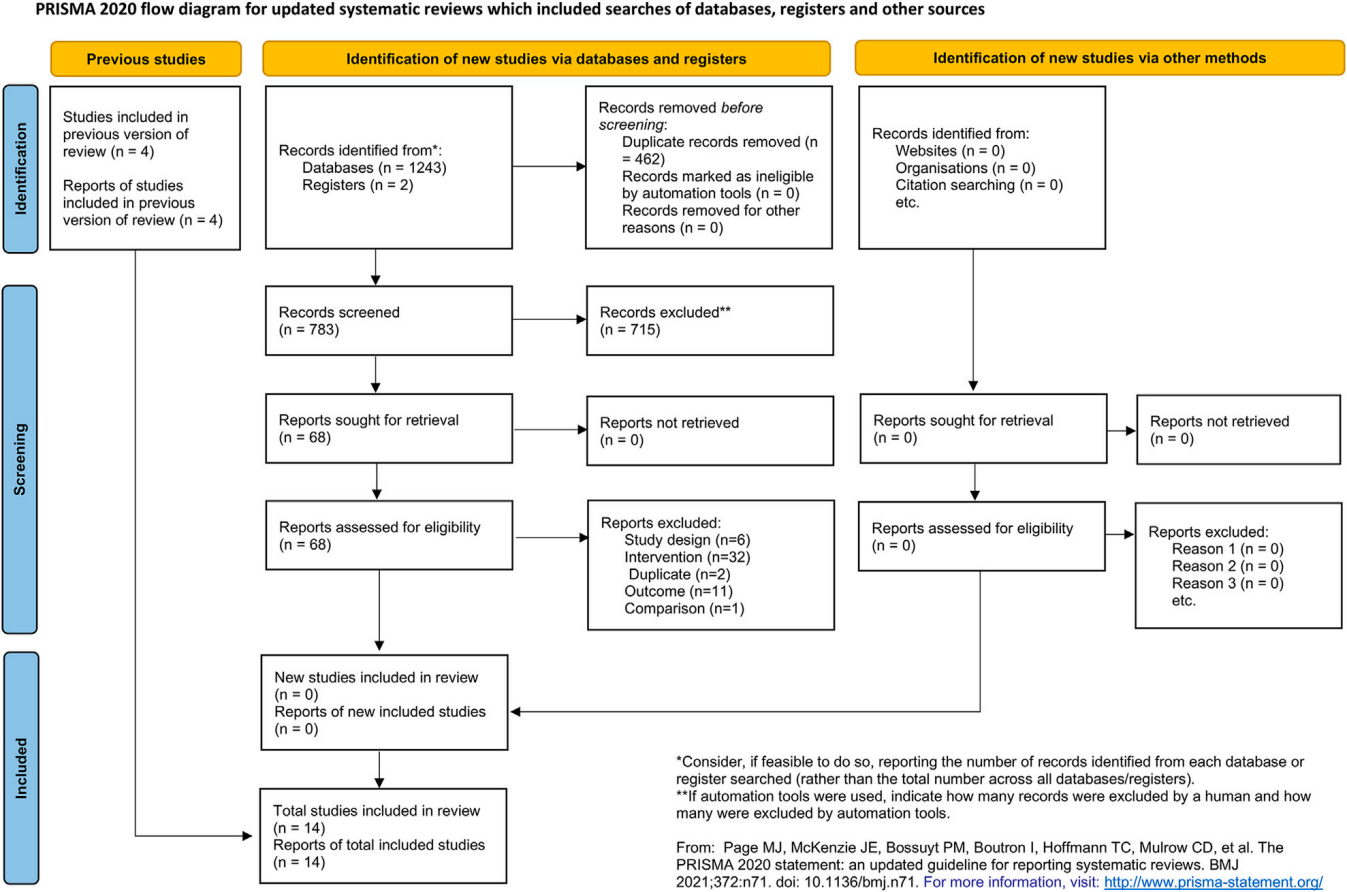
PRISMA flow diagram.

A total of 1,245 citations were retrieved from the database search. Four hundred and sixty-two citations were excluded due to duplication. Seven hundred and fifteen citations were excluded after reading the titles and abstracts. After reading the full-texts, 52 citations were excluded. No additional publication was identified by searching the references of the included studies, related reviews, and meta-analyses. Two studies were found to be awaiting classification due to insufficient information. Fourteen studies were included in the final quantitative analysis. The full list of excluded studies with reasons is provided in Supplementary material S4. The flow chart of the study selection is shown in Figure 1.

### Characteristics of the Included Studies

Fourteen studies including 1,586 participants were included in this meta-analysis (Zhaoyi et al., 2010; Xiaoming 2011; Xinyi 2011; Lina 2012; Yu and Yuan 2012; Yuting 2012; Yinzhong 2013; Yang 2016; Cuirong 2017; Fan 2017; Hongyun 2018; Jingzu et al., 2019; Bin 2020; Shufang 2020). Three studies did not report the detailed composition of medicine, nor did they clearly state whether the Chinese patent medicine was used. Therefore, we excluded these three studies from the sensitivity analysis and re-analyzed the data to evaluate their impact on the pooled effect (Yinzhong 2013; Yang 2016; Fan 2017). Two studies compared the efficacy of JGSQW and hypoglycemic agents directly, while other studies used a combination of hypoglycemic agents and JGSQW in the experimental group (Zhaoyi, Guang and Hongwei 2010; Xiaoming 2011).

The duration of T2DM in the participants in the included studies was between 3 and 8 years. Five studies included patients with T2DM without complications (Xiaoming 2011; Xinyi 2011; Lina 2012; Yuting 2012; Yinzhong 2013). Six studies included participants with diabetic nephropathy (Yang 2016; Cuirong 2017; Hongyun 2018; Jingzu, Jigong, Peixin and Yu 2019; Bin 2020; Shufang 2020), one included participants with diabetic peripheral neuropathy (Yu and Yuan 2012), and two included participants with hyperlipidemia (Zhaoyi, Guang and Hongwei 2010; Fan 2017). Detailed characteristics of these 14 studies can be seen in Table 2.

**TABLE 2:** Characteristics of the included studies.

Fan Jia, 2016	Yang Xiao, 2016	Yinzhong Li, 2012	Xinyi Zhang, 2011	Zhaoyi Huang, 2010	Xiaoming Yang, 2011	Lina Chen, 2012	Cuirong Hou, 2017	Hongyu Sun, 2018	Jingzu Zhang, 2019	Shufang Geng, 2020	Bin Jiang, 2020	Yu Zhao, 2012	Yuting Guo, 2012	First author and year
China	China	China	China	China	China	China	China	China	China	China	China	China	China	Country
RCT	RCT	RCT	RCT	RCT	RCT	RCT	RCT	RCT	RCT	RCT	RCT	RCT	RCT	Study design
52.47 ± 2.85/52.07 ± 2.73	46.2 ± 9.7	34–48/32–46	56.4 ± 10.9/60.6 ± 2.1	48.5 ± 5.8/47.2 ± 5.2	52.65 ± 10.52/51.86 ± 10.70	54.21 ± 26.42/53.54 ± 23.32	61.38 ± 10.36/61.84 ± 10.46	55.6 ± 11.2/54.8 ± 11.4	53.1 ± 3.1/53.7 ± 3.1	55.28 ± 4.12/55.36 ± 4.18	54.65 ± 5.5/55.20 ± 5.24	56.29 ± 4.33/57.43 ± 3.89	65.7 ± 2.1/63.1 ± 8.1	Age (mean ± standard deviation; treatment/control)
65/65	24/24	39/39	100/100	35/35	120/120	60/60	45/45	33/31	118/118	37/37	60/60	30/26	30/30	Sample size (treatment/control)
32/30	12/11	18/19	40/50	16/15	50/56	15/17	21/22	15/14	50/51	12/14	25/26	14/8	15/15	Number of females (treatment/control)
NR	NR	NR	6.5 ± 1.8/7.1 ± 1.3	NR	3.92 ± 3.45/3.72 ± 4.86	NR	8.31 ± 2.16/8.62 ± 2.32	NR	3.1 ± 0.3/3.2 ± 0.3	3.87 ± 0.51/3.91 ± 0.55	3.26 ± 1.43/3.5 ± 1.51	6–20/6–20	5.3 ± 2.0/5.7 ± 2.1	Duration (years) of T2DM (treatment/control)
Glimepiride	Only lifestyle intervention	Insulin, metformin	Metformin, Xiaoke Pill, simvastatin	NR	NR	Glimepiride	Glimepiride	Insulin + glimepiride	Hypoglycemic agents	Hypoglycemic agents	Hypoglycemic agents	Mecobalamin, hypoglycemic agents	Glimepiride	Co-intervention
JGSQW	JGSQW	JGSQW, 10g, tid	JGSQW, 6g, tid	JGSQW, bid	JGSQW, 5g, tid	JGSQW, 5g, tid	JGSQW, 6g, bid	JGSQW, 6g, bid	JGSQW, bid	JGSQW, bid	JGSQW, bid	JGSQW, 6g, bid	JGSQW, 6g, tid	treatment intervention
NR	NR	NR	NR	Simvastatin, metformin	Xiaoke Pill	NR	NR	NR	NR	NR	NR	Fufang Danshen Pian	NR	Comparator intervention
NR	6W	3 months	60d	12W	4W	4W	NR	NR	4W	4W	4W	4W	12W	Duration
NR	NR	NR	NR	NR	NR	NR	NR	NR	NR	NR	NR	NR	NR	Funding
NR	NR	8.3 ± 1.1/8.4 ± 1.2	NR	7.56 ± 0.89/7.92 ± 0.78	8.78 ± 1.64/8.67 ± 2.08	8.43 ± 1.05/8.34 ± 1.24	NR	NR	NR	NR	NR	NR	9.7 ± 1.6/9.6 ± 1.8	Baseline HbA1c (%) (treatment/placebo)
NR	24.7 ± 2.1/25.6 ± 2.2	27.3 ± 2.50/27.6 ± 2.17	24.91 ± 0.44/24.87 ± 0.24	NR	NR	NR	NR	NR	NR	NR	NR	NR	24.14 ± 2.34/25.34 ± 2.68	Baseline BMI (kg/m^2^) (treatment/placebo)
11.21 ± 2.37/11.29 ± 3.21	12.1 ± 2.6/11.2 ± 2.6	10.5 ± 0.8/10.6 ± 0.7	9.12 ± 2.18/9.33 ± 2.07	10.12 ± 0.28/10.89 ± 0.97	9.8 ± 2.56/9.78 ± 2.54	9.06 ± 1.87/9.15 ± 2.87	10.98 ± 2.56/11.18 ± 3.72	NR	12.09 ± 3,26/12.15 ± 3.32	NR	NR	7.02 ± 2.32/7.38 ± 2.32	12.4 ± 2.2/12.1 ± 2.1	Baseline FBG (mmol/L)
13.73 ± 4.33/13.72 ± 4.58	NR	12.9 ± 0.6/12.1 ± 0.6	14.26 ± 3.54/14.57 ± 3.33	15.12 ± 0.61/16.65 ± 0.70	15.02 ± 4.35/15.01 ± 4.19	15.52 ± 4.73/15.76 ± 4.85	13.75 ± 4.26/13.67 ± 4.56	NR	13.87 ± 4.59/13.89 ± 4.66	NR	NR	NR	14.0 ± 2.6/13.4 ± 3.1	Baseline 2hBG (mmol/L)
NR	NR	1.14 ± 0.15/1.15 ± 0.08	NR	0.87 ± 0.06/0.83 ± 0.07	1.26 ± 0.53/1.27 ± 0.45	NR	NR	NR	NR	NR	NR	NR	NR	Baseline HDL-C (mmol/L)
NR	NR	3.5 ± 0.4/3.4 ± 0.6	NR	5.14 ± 0.91/4.98 ± 0.15	NR	NR	NR	NR	NR	NR	NR	NR	NR	Baseline LDL-C (mmol/L)
NR	6.2 ± 0.5/6.5 ± 0.4	NR	6.28 ± 1.32/5.87 ± 1.18	6.23 ± 0.89/6.19 ± 0.17	6.21 ± 1.13/5.76 ± 1.16	NR	NR	NR	NR	NR	NR	NR	NR	Baseline TC (mmol/L)
NR	2.6 ± 0.2/2.5 ± 0.2	2.60 ± 0.4/2.58 ± 0.6	2.04 ± 0.89/2.13 ± 1.2	2.59 ± 0.86/2.67 ± 0.32	2.17 ± 0.2/1.93 ± 0.88	NR	NR	NR	NR	NR	NR	NR	NR	Baseline TG (mmol/L)
NR	NR	6.1 ± 0.3/6.6 ± 0.6	NR	6.77 ± 0.81/6.55 ± 0.63	7.73 ± 1.5/7.74 ± 1.5	7.34 ± 1.03/7.76 ± 1.08	NR	NR	NR	6.91 ± 1.4/7.62 ± 1.63	NR	NR	7.3 ± 2.3/8.0 ± 2.1	HbA1c (%) (treatment/control)
NR	NR	26.9 ± 1.18/26.6 ± 1.54	23.21 ± 0.15/24.66 ± 0.38	NR	NR	NR	NR	NR	NR	NR	NR	NR	NR	BMI (kg/m^2^) (treatment/control)
6.92 ± 1.22/7.2 ± 1.68	NR	6.8 ± 0.6/7.8 ± 0.6	7.11 ± 1.52/8.04 ± 1.75	6.92 ± 0.54/6.35 ± 0.76	7.1 ± 1.77/7.67 ± 2.55	6.92 ± 1.32/7.43 ± 1.34	6.88 ± 1.26/7.21 ± 1.68	6.84 ± 4.32/7.2 ± 1.67	7.17 ± 1.69/6.67 ± 1.25	7.1 ± 2.07/9.26 ± 2.34	7.16 ± 1.68/9.58 ± 2.26	6.76 ± 2.57/6.68 ± 2.12	7.6 ± 1.1/8.4 ± 1.3	FBG (mmol/L)
8.02 ± 3.27/8.87 ± 3.32	NR	7.3 ± 0.5/8.7 ± 0.7	11.09 ± 3.87/12.90 ± 1.7	9.56 ± 1.42/9.27 ± 1.34	9.89 ± 3/10.93 ± 3.77	9.23 ± 3.64/10.89 ± 3.65	8.16 ± 4.34/8.63 ± 3.41	6.87 ± 1.24/8.62 ± 3.4	8.65 ± 3.23/7.91 ± 3.26	7.97 ± 3.67/10.75 ± 3.53	8.64 ± 3.22/9.92 ± 3.17	NR	8.5 ± 2.5/9.8 ± 1.3	2hBG (mmol/L)
NR	NR	1.22 ± 0.16/1.16 ± 0.07	NR	1.21 ± 0.10/1.02 ± 0.08	1.28 ± 0.45/1.28 ± 0.45	NR	NR	NR	NR	NR	NR	NR	NR	HDL-C (mmol/L)
NR	NR	NR	NR	3.73 ± 0.16/3.21 ± 0.12	NR	NR	NR	NR	NR	NR	NR	NR	NR	LDL-C (mmol/L)
NR	3.8 ± 0.6/5.9 ± 0.3	NR	5.27 ± 0.83/5.46 ± 1.2	4.14 ± 0.59/4.15 ± 0.66	4.82 ± 0.88/5.82 ± 2.45	NR	NR	NR	NR	NR	NR	NR	NR	TC (mmol/L)
NR	1.4 ± 0.3/2.2 ± 0.1	1.1 ± 0.4/1.4 ± 0.2	1.5 ± 0.27/1.96 ± 1.22	1.71 ± 0.24/1.75 ± 0.31	1.65 ± 0.19/1.85 ± 0.73	NR	NR	NR	NR	NR	NR	NR	NR	TG (mmol/L)
Hyperlipidemia	DN	NR	NR	Hyperlipidemia	NR	NR	DN	DN	DN	DN	DN	DPN	NR	Comorbidity
NR	NR	No adverse effects were observed	NR	NR	NR	NR	NR	NR	NR	NR	NR	NR	Hypoglycemia	Adverse event
No	No	No	No	No	No	No	No	No	No	No	No	No	No	Quality control reported?
No	No	No	No	No	No	No	No	No	No	No	No	No	No	Chemical analysis reported?

NR: not reported; JGSQW: Jīn-Guì Shèn-Qì Wán; DN: diabetic nephropathy; DPN: diabetic peripheral neuropathy; T2DM: type 2 diabetes mellitus; HbA1c: glycated hemoglobin; FBG: fasting blood glucose; 2hBG: 2-h postprandial glucose; HDL-C: high-density lipoprotein cholesterol; LDL-C: low-density lipoprotein cholesterol; TC: total cholesterol; TG: triglyceride; BMI: body mass index.

### Risk of Bias Assessment

**FIGURE 2:**
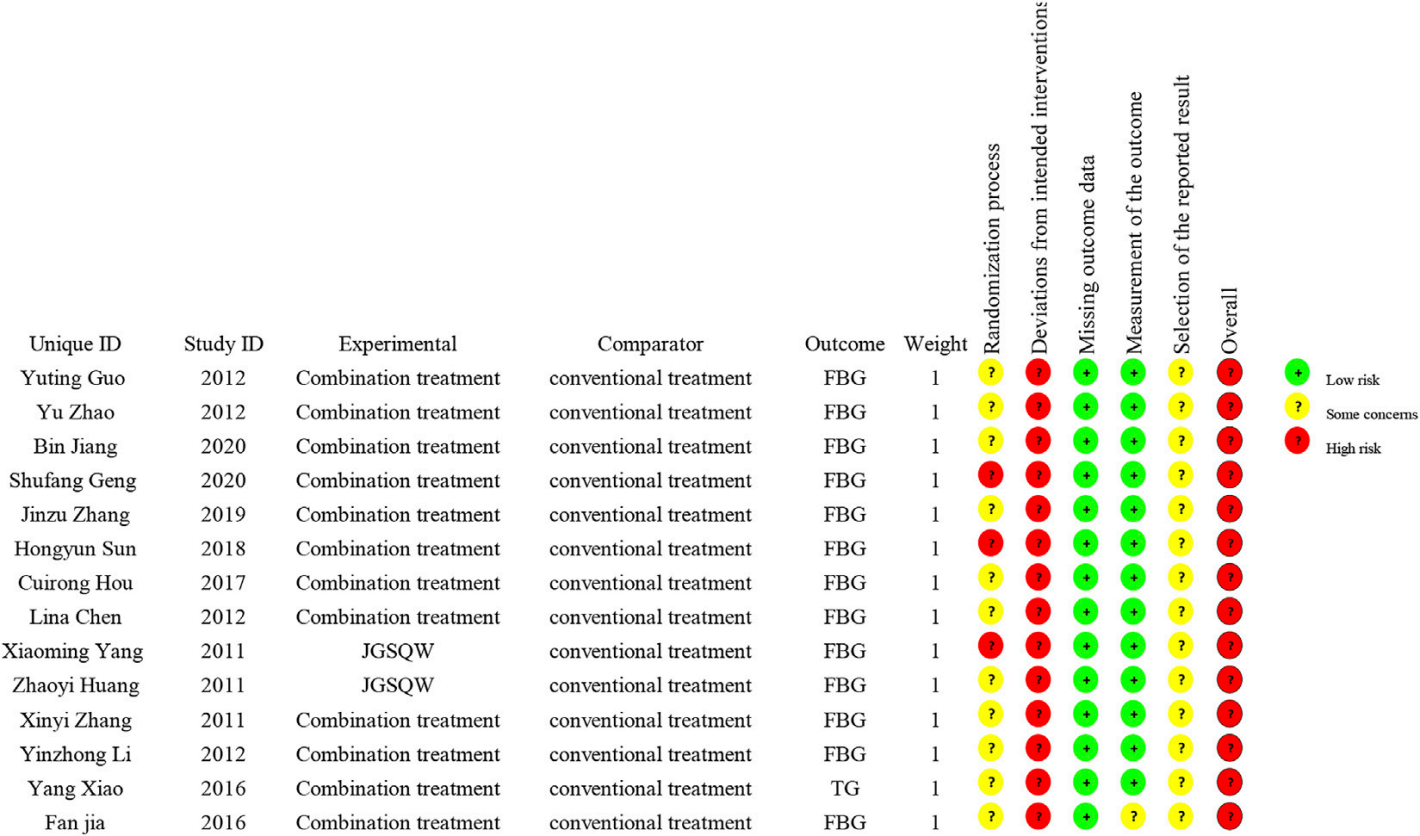
Risk-of-bias graph.

**FIGURE 3:**
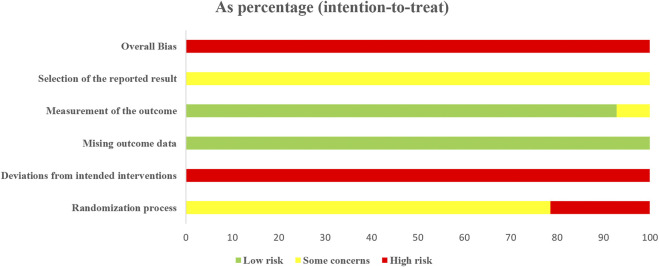
Risk-of-bias summary.

All 14 included studies were judged to be at high risk of bias. The results of the risk of bias assessment are shown in Figures 2 and 3 and the details are provided in Supplementary Material S5.

### Effect of JGSQW on T2DM

#### HbA1c

**FIGURE 4:**
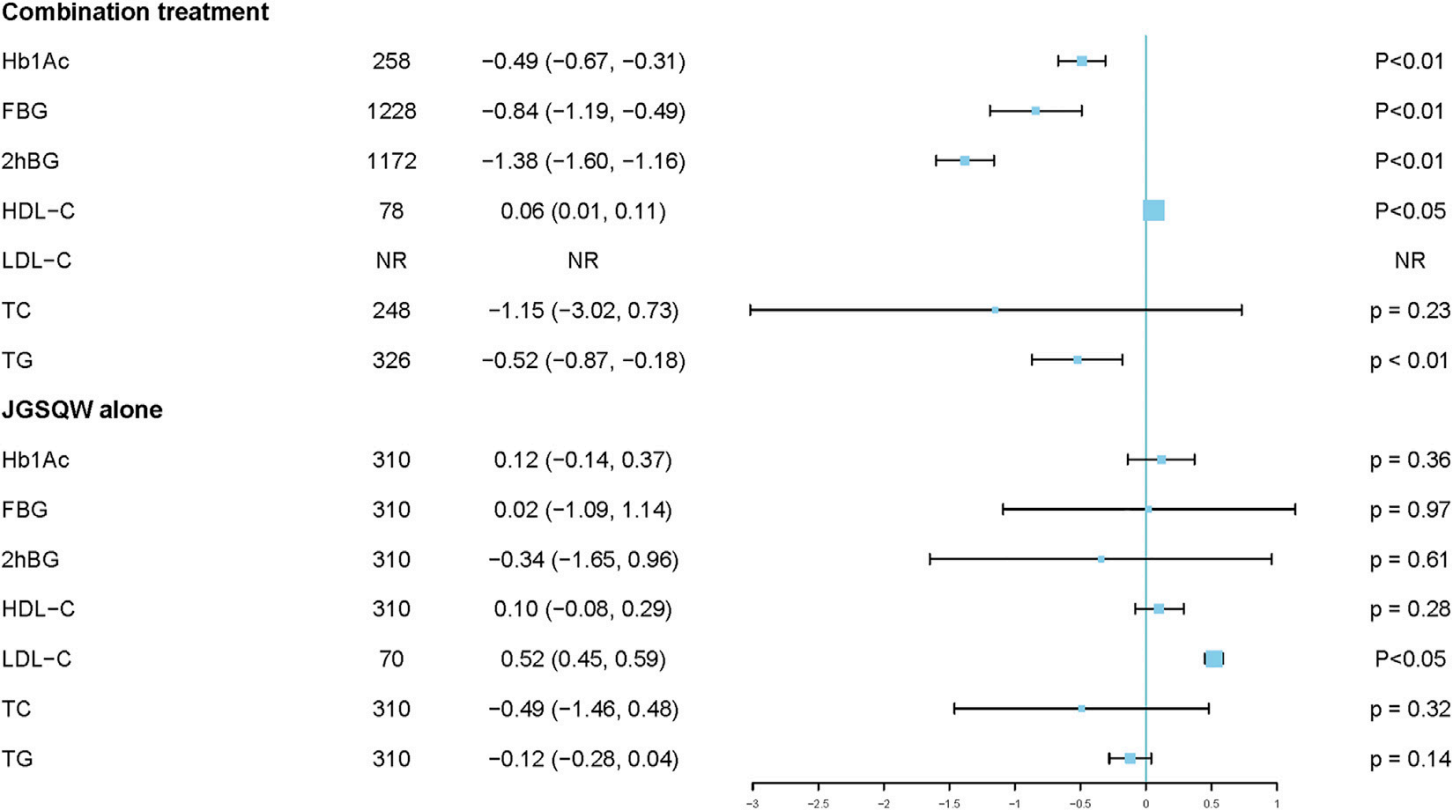
Summary of the main findings.

Three studies including 258 participants reported effects on HbA1c by combination treatment compared with hypoglycemic agents alone (Lina 2012; Yuting 2012; Yinzhong 2013). Pooled results indicated that combination treatment results in a reduction in HbA1c (MD −0.49%; 95% CI −0.67 to −0.31; *p* <0.01; *I* ^*2*^ = 0%) (Figure 4; Supplementary Material S6.1). Sensitivity analyses indicated that the result was robust (Supplementary Material S6.2). Subgroup analyses showed no interaction with age (*p* = 0.70) (Supplementary Material S6.3).

We performed further comparisons based on specific medicines used in the studies. Compared with glimepiride 2 mg qd alone, combination treatment of JGSQW patent medicine and glimepiride 2 mg qd decreased HbA1c (MD −0.45%; 95% CI −0.81 to −0.09; *p* = 0.01; *I* ^*2*^ = 0%) (Lina 2012; Yuting 2012). Compared with metformin plus insulin, combination treatment of JGSQW patent medicine and metformin plus insulin decreased HbA1c (MD −0.50%; 95% CI −0.71 to −0.29; *p* < 0.01) (Yinzhong 2013) (Supplementary Material S6.4).

Two studies including 310 participants reported an effect of JGSQW on HbA1c compared with hypoglycemic agents (Zhaoyi, Guang and Hongwei 2010; Xiaoming 2011). The pooled effect showed no significant difference (MD –0.12%; 95% CI –0.14 to 0.37; *P* = 0.36; *I* ^*2*^ = 0%) (Figure 4) (Supplementary Material S6.5). The sensitivity analysis using a fixed-effects model did not modify this result significantly (Supplementary Material S6.5).

#### FBG

Eleven studies including 1,228 participants reported the effect of JGSQW on FBG by combination treatment compared with hypoglycemic agents alone (Xinyi 2011; Lina 2012; Yu and Yuan 2012; Yuting 2012; Yinzhong 2013; Cuirong 2017; Fan 2017; Hongyun 2018; Jingzu, Jigong, Peixin and Yu 2019; Bin 2020; Shufang 2020). The pooled results indicated that combination treatment resulted in a reduction in FBG, but the variation between studies was large (MD −0.84; 95% CI −1.19 to −0.49; prediction interval −1.99 to 0.31; *p* <0.01; *I* ^*2*^ = 76%) (Figure 4) (Supplementary Material S6.6). Sensitivity analyses indicated that this result was robust (Supplementary Material S6.7). Subgroup analyses according to different ages, comorbidity of participants, and forms of JGSQW showed no significant interaction with these factors (*P* = 0.90, 0.99, and 0.30, respectively) (Supplementary Material S6.8–S6.10). The subgroup analysis according to different levels of control group (≥9 mmol/L or <9 mmol/L) showed significant subgroup difference (*P* <0.01) and the heterogeneities in these two subgroups were decreased (*I* ^*2*^ = 40 and 0%, respectively) (Supplementary Material S6.11).

Further comparisons based on specific medicines used in the studies showed that, compared with glimepiride 2 mg qd, combination treatment of JGSQW patent medicine and glimepiride 2 mg qd decreased FBG (MD −0.54; 95% CI −0.86 to −0.22; *P* < 0.01; *I* ^*2*^ = 0%) (Lina 2012; Yuting 2012; Cuirong 2017). Compared with mixed oral agents, combination treatment of JGSQW patent medicine and mixed oral agents had no significant effect on FBG (MD 0.08; 95% CI −1.15 to 1.31; *P* = 0.90) (Yu and Yuan 2012). Compared with glimepiride 2 mg qd + insulin + acarbose 1# tid, combination treatment of JGSQW decoction and glimepiride 2 mg qd + insulin + acarbose 1# tid decreased FBG (MD −1.66; 95% CI −3.07 to −0.24; *P* = 0.02; *I* ^*2*^ = 93%) (Jingzu, Jigong, Peixin and Yu 2019; Bin 2020; Shufang 2020). Compared with glimepiride 2 mg qd + insulin, combination treatment of JGSQW patent medicine and glimepiride 2 mg qd + insulin had no effect on FBG (MD −0.36; 95% CI −1.95 to 1.23; *P* = 0.66) (Hongyun 2018). Compared with metformin + Xiaoke Pill, combination treatment of JGSQW patent medicine and metformin + Xiaoke Pill decreased FBG (MD –0.93; 95% CI −1.38 to −0.48; *p* < 0.01) (Xinyi 2011). Compared with metformin + insulin, combination treatment of JGSQW patent medicine and metformin + insulin decreased FBG (MD −1.00; 95% CI −1.27 to −0.73; *p* <0.01) (Yinzhong 2013). Compared with glimepiride 2 mg qd, combination treatment of JGSQW decoction and glimepiride 2 mg qd had no effect on FBG (MD −0.28; 95% CI −0.78 to 0.22; *P* = 0.28) (Fan 2017) (Supplementary Material S6.12).

Two studies including 310 participants reported effects of JGSQW or hypoglycemic agents on FBG (Zhaoyi, Guang and Hongwei 2010; Xiaoming 2011). Zhaoyi et al. (2010) reported that, compared with Xiaoke Pills, JGSQW decreased FBG (MD −0.57; 95% CI −1.13 to −0.01), while Xiaoming (2011) reported that JGSQW was less effective than metformin hydrochloride tablets (Supplementary Material S6.13).

#### 2hBG

Ten studies including 1,172 participants reported the effects of combination treatment compared with hypoglycemic agents alone on 2hBG (Xinyi 2011; Lina 2012; Yuting 2012; Yinzhong 2013; Cuirong 2017; Fan 2017; Hongyun 2018; Jingzu, Jigong, Peixin and Yu 2019; Bin 2020; Shufang 2020). The pooled results indicated that combination treatment results in a reduction in 2hBG (MD –1.38; 95% CI –1.60 to –1.16; prediction interval –1.63 to –1.12; *p* <0.01; *I* ^*2*^ = 0%) (Figure 4; Supplementary Material S6.14). Sensitivity analyses indicated that this result was robust (Supplementary Material S6.15). Subgroup analyses according to different ages, comorbidity of participants, and forms of JGSQW showed no significant interactions (*P* = 0.89, 0.43, and 0.58, respectively) (Supplementary Material S6.17–S6.19).

Further comparisons based on specific medicines used in the studies showed that, compared with glimepiride 2 mg qd, combination treatment of JGSQW patent medicine and glimepiride 2 mg qd decreased 2hBG (MD −1.25; 95% CI −1.96 to −0.53; *P* < 0.01; *I* ^*2*^ = 0%) (Lina 2012; Yuting 2012; Cuirong 2017). Compared with glimepiride 2 mg qd + insulin + acarbose 1# tid, combination treatment of JGSQW decoction and glimepiride 2 mg qd + insulin + acarbose 1# tid decreased 2hBG (MD −1.41; 95% CI −2.44 to −0.37; *p* < 0.01; *I* ^*2*^ = 58%) (Jingzu, Jigong, Peixin and Yu 2019; Bin 2020; Shufang 2020). Compared with glimepiride 2 mg qd + inslin, combination treatment of JGSQW patent medicine and glimepiride 2 mg qd + inslin decreased 2hBG (MD −1.75; 95% CI −3.02 to −0.48; *P* < 0.01) (Hongyun 2018). Compared with metformin + Xiaoke Pill, combination treatment of JGSQW patent medicine and metformin + Xiaoke Pill decreased 2hBG (MD −1.81; 95% CI −2.64 to −0.98; *p* < 0.01) (Xinyi 2011). Compared with metformin + insulin, combination treatment of JGSQW patent medicine and metformin + insulin decreased 2hBG (MD −1.40; 95% CI −1.67 to −1.13; *P* < 0.01) (Yinzhong 2013). Compared with glimepiride 2 mg qd, combination treatment of JGSQW decoction and glimepiride 2 mg qd had no significant effect on 2hBG (MD −0.85; 95% CI −0.98 to 0.28; *p* = 0.14) (Fan 2017) (Supplementary Material S6.19).

Two studies including 310 participants reported the effects of JGSQW compared with hypoglycemic agents on 2hBG (Zhaoyi, Guang and Hongwei 2010; Xiaoming 2011). Zhaoyi et al. (2010) reported that, compared with Xiaoke Pills, JGSQW decreased 2hBG (MD −1.04; 95% CI −1.90 to −0.18), while Xiaoming (2011) reported no difference compared with metformin hydrochloride tablets (Supplementary Material S6.20).

#### HDL-C

One study including 78 participants reported a significant increase in HDL-C with combination treatment compared with hypoglycemic agents alone (MD 0.06; 95% CI 0.01–0.11) (Yinzhong 2013) (Supplementary Material S6.21).

Two studies including 310 participants reported the effects of JGSQW compared with hypoglycemic agents on HDL-C (Zhaoyi, Guang and Hongwei 2010; Xiaoming 2011). Zhaoyi et al. (2010) reported no difference compared with Xiaoke Pills, while Xiaoming (2011) reported significant difference compared with metformin hydrochloride tablets (Supplementary Material S6.22).

#### LDL-C

One study reported that, compared with Xiaoke Pills, JGSQW increased the level of LDL-C (MD 0.52; 95% CI 0.45–0.59) (Zhaoyi, Guang and Hongwei 2010) (Supplementary Material S6.23).

#### TC

Two studies including 248 participants reported the effect of combination treatment compared with hypoglycemic agents alone on TC (Xinyi 2011; Yang 2016). Due to significant heterogeneity among studies, the data were not pooled. Yang (2016) reported that combination treatment of JGSQW decoction and lifestyle intervention decreased the level of TC compared with lifestyle intervention alone (MD −2.10; 95% CI −2.37 to −1.83), while Xinyi (2011) showed no difference for JGSQW patent medicine compared with metformin + Xiaoke Pill (MD −0.19; 95% CI −0.48 to 0.10) (Supplementary Material S6.24).

Two studies including 310 participants reported the effect of JGSQW compared with hypoglycemic agents on TC (Zhaoyi, Guang and Hongwei 2010; Xiaoming 2011). Zhaoyi et al. (2010) reported no difference compared with Xiaoke Pills, while Xiaoming (2011) reported significant difference compared with metformin hydrochloride tablets (MD −1.00; 95% CI −1.47 to −0.53) (Supplementary Material S6.25).

#### TG

Three studies including 326 participants reported the effect of combination treatment compared with hypoglycemic agents alone on TG (Xinyi 2011; Yinzhong 2013; Yang 2016). Due to significant heterogeneity among studies, the data were not pooled. Compared with metformin + Xiaoke Pill, combination treatment of JGSQW patent medicine and metformin + Xiaoke Pill decreased 2hBG (MD −0.46; 95% CI −0.70 to −0.22; *P* < 0.01) (Xinyi 2011). Compared with metformin + insulin, combination treatment of JGSQW patent medicine and metformin + insulin decreased 2hBG (MD −0.30; 95% CI −0.44 to −0.16; *P* < 0.01) (Yinzhong 2013). Compared with lifestyle intervention, combination treatment of JGSQW decoction and lifestyle intervention had no effect on 2hBG (MD −0.80; 95% CI −0.93 to 0.67; *p* < 0.01) (Yang 2016) (Supplementary Material S6.26).

Two studies including 310 participants reported the effects of JGSQW compared with hypoglycemic agents on TG (Zhaoyi, Guang and Hongwei 2010; Xiaoming 2011). The pooled effect showed no significant difference between these interventions (MD –0.12; 95% CI –0.28 to 0.04; *p* = 0.14; *I* ^*2*^ = 64%) (Figure 4; Supplementary Material S6.27). The sensitivity analysis using a fixed-effects model did not modify this result significantly (Supplementary Material S6.27).

#### Publication Bias

A funnel plot of data relating to 2hBG showed symmetry (Supplementary Material S6.28), and Egger’s test showed no statistical significance (*p* = 0.9994). A funnel plot of the FBG data showed asymmetry (Supplementary Material S6.29) and the result of Egger’s test showed no statistical significance (*p* = 0.8944).

#### Adverse Events

Two studies including 138 participants reported adverse events as outcomes (Yuting 2012; Yinzhong 2013). The work of Yuting (2012), which included 60 participants, reported that five hypoglycemic events occurred in the experimental group, while six such events occurred in the control group. The work of Yinzhong (2013), which included 78 participants, reported that no adverse events occurred during the study.

### Phytochemical Profile and Potential Mechanisms of JGSQW on T2DM in the Experimental Studies

A total of 102 components were retrieved by searching the ingredients of eight herbs. After removing 11 duplicates, we identified a total of 91 active components for JGSQW. The detailed information of the components of herbal medicines and JGSQW are provided in Supplementary Material S7.1–S7.9. A total of 40 preclinical studies investigated the interaction between JGSQW and anti-diabetic agents and summarized the potential mechanisms of JGSQW on T2DM. The beneficial effects and potential mechanisms are summarized in Table 3.

**TABLE 3:** Herb-drug interactions with the anti-diabetic drugs and the potential mechanisms of JGSQW as a treatment for T2DM reported by preclinical studies.

References	Anti-diabetic drugs	Beneficial effects	Potential mechanisms
Chen et al. (2008), Chen and Zeng (2012), Chen and Yang (2013), Chen et al. (2014), Chen (2012), Cheng et al. (2001), Gao (2008), Hirotani et al. (2010), Hirotani Ikeda, Yamamoto, et al. (2007), Hu (2012), Jin et al. (2011), Jin et al. (2012a), Jin et al. (2012b), Jin and Pan (2008), Li (2017), Li et al. (2017), Li (2012), Liu et al. (2013), Liu and Hu (2011), Liu and Hu (2012), Pan (2008), Zhang, Qin, et al. (2014), Zhao (2019)	Metformin, rosiglitazone	Improving glucose metabolism	Reducing hepatic gluconeogenesis, insulin resistance, chronic low-grade tissue inflammation, increasing leptin, skeletal muscle InsR, hepatic GLUT2, PEPCK expression, increasing HK and PFK activity, and promoting glucose oxidation and utilization
Gao (2008); Pan (2008); Liu and Hu (2011); Chen (2012); Chen and Zeng (2012); Li (2012); Liu, Zhang, Yu, Yan and Zheng (2013); Chen, Wang and Zheng (2014); Li (2017); Li, Qiu, Zhao, Deng, Liu and Hu (2017)	Rosiglitazone	Reducing blood lipids	—
Chen (2008); Chen and Zheng (2008); Hirotani, Ikeda, Ikeda, et al. (2007)	Not used	Improving gastrointestinal functions	Improving gastric emptying, intestinal propulsion, levels of substance P in the intermuscular plexus of the gastric sinus, normalizing or suppressing the small intestinal disaccharidase activity and the epithelial cell proliferation mediated by GLP-2.
Gao (2008); Pan (2008); Jin, Chen and Li (2011); Jin, Chen and Li (2012a); Jin, Chen and Li (2012b); Chen (2012); Li (2012); Wang and Yang (2012); Yao et al. (2016); Huang (2017)	Rosiglitazone	Kidney protection	Improving renal function, increasing nitric oxide and nitric oxide synthase levels, and promoting the repairment of damaged kidney tissues; decreasing UAER, ET, CTGF, and TGF-β1 levels and increasing IGF-1 levels in kidney tissues; affecting the expression of apoptosis-related genes Bcl-2 and Bax
Chen and Wang (2015); Liang et al. (2002); Liu et al. (2004); Shi et al. (2011a); Shi et al. (2011b); Wang and Yang (2012); Wang et al. (2019); Zhang, Chen, et al. (2014)	Metformin	Protection of nerve tissue and function	Enhancing the expression of Ng, mGluR5, NT-3, and nNOS in the hippocampal CA1 region of type 2 diabetic rats; reducing aldose reductase activity (AR), sorbitol (SNS) concentration, superoxide dismutase (SOD) activity, and serum malondialdehyde (MDA) levels; protecting against nerve damage; improving sciatic nerve conduction velocity
Huang (2011); Wang et al. (2012)	Metformin	Sexual function improvement	Increasing testosterone, Nitric Oxide (NO), sex hormone binding globulin (SHBG), androgen levels

## Discussion

### Main Results and Application of This Research

Some important findings have emerged from this analysis of data from 14 studies. Combining JGSQW with hypoglycemic agents does not increase the incidence of adverse events such as hypoglycemia, indicating that JGSQW is safe in the clinical practice. HbA1c, FBG, and 2hBG are the three parameters for evaluating the glycemic control. Among them, HbA1c reflects the average glycemic level over the past few months and is therefore considered the gold standard for evaluating the glycemic control. In this study, compared with a control group, the combined use of JGSQW was found to reduce the level of HbA1c, enhancing the glycemic control level of patients with T2DM.

Changes in both FBG and 2hBG will cause changes in HbA1c levels. This study found less reduction of 2hBG than of FBG, which means that JGSQW mainly decreases these levels by reducing 2hBG. The glucose lowering effects of JGSQW were consistent regardless of the form of JGSQW. We did not find significant heterogeneity in the results of studies on HbA1c and 2hBG, suggesting that the effect of JGSQW is consistent across different settings. We found substantial heterogeneity in the results of studies on FBG. The subgroup analysis on different levels of control group showed that the heterogeneity decreased significantly and there was a significant difference in the effect size among different subgroups, indicating that the effect of JGSQW may depend on the patients’ FBG levels. The significant heterogeneity in the results of direct comparisons between JGSQW and hypoglycemic agents may be due to the different hypoglycemic agents used in the studies. Due to the lack of a placebo control, the net effect for JGSQW remains unknown.

Inconsistency was also found among the results on lipid metabolism, limiting the clinical application of those findings. On examining the data, we found that the differences in lipid-lowering efficacy may be due to different comorbidities (with and without hyperlipidemia) of participants. Overall, the influence of JGSQW on lipid metabolism is unclear and its effect on type 2 diabetic patients with hyperlipidemia deserves further investigation.

In this study, we identified 91 active ingredients of JGSQW, of which the most common are Sitosterol, Stigmasterol, (−)-taxifolin, Alisol B, and (+)-catechin. These components lay the material foundation for the effect of JGSQW. However, the components of JGSQW are not only the addition of compounds in various Chinese herbal medicine but also the possibility of new compounds in the preparation process, which may be neglected in the network pharmacology. Therefore, the components of JGSQW need to be verified through experiments in future research.

### Risk of Bias

Using the latest ROB2 tool, we found that all included studies had a high risk of bias, mainly caused by deviations from intended interventions. In addition, most studies failed to report in detail their randomization method, including the generation of randomized sequences, and none described allocation concealment. These factors and an absence of intention to treat analysis may have led to exaggerated treatment effects. Overall, due to the clear methodological shortcomings of the included studies, there is uncertainty in our judgment of the overall results.

### Non-Reporting Biases

In general, the presence of publication bias leads to a pooled result that favors the intervention group. In the present study, we tested the publication bias by the funnel plot and Egger’s test for FBG and 2hBG. The funnel plot for 2hBG was asymmetric, while the statistical results suggested the absence of publication bias. This contradictory result may be due to the low power of the statistical test. We did not perform publication bias tests for other outcomes due to low numbers of studies reporting these. An effective method to assess the presence of publication bias is to compare the published studies with the study registration information. In view of the low statistical power of the funnel plot and Egger’s test and the fact that none of the clinical trials included in this study were registered, we cannot fully exclude the possibility of publication bias.

### Novelty and Limitations of This Research

This study is the first high-quality systematic review to summarize the effect of JGSQW in the treatment of T2DM. Compared with previous studies, 10 new studies were included in this systematic review. The methodological quality of the present study was evaluated using the AMSTAR 2 method and was found to be high. In addition, we have provided detailed Supplementary Materials, allowing repetition and further evaluation of this research. This is also the first study to comprehensively summarize the evidence on the mechanisms by which JGSQW may affect T2DM in the experimental studies. The results of our study will be of great value for future basic and clinical research.

Despite the use of high-quality methodology, inevitable significant limitations remain. All of the clinical trials had clear methodological flaws and it is difficult to quantify the impact of these risks of bias on the pooled results. Secondly, in this study, there are differences in the numbers of studies and participants for different outcomes. This is partly because different studies observed and consequently reported different outcomes. In order to decrease the diversity in outcomes and facilitate standardization of the outcome measurement across the trials, efforts to develop core outcome sets for conditions have been made in recent years (Williamson et al., 2012; Boers et al., 2014; Bain et al., 2016; Kirkham et al., 2016; Kirkham et al., 2017; Williamson et al., 2017; Kirkham et al., 2019; Egan et al., 2020). Despite this significant progress, further work is needed in the standardization of outcome reporting. Consistency in the reporting of study results can be effectively improved by adopting a widely agreed core outcome set and by pre-registering the studies before they are conducted. In addition to the limitations mentioned above, incomplete data reporting impedes further analysis. Since most of the included studies are published in Chinese journals, there may be a certain degree of language bias. Overall, the results of this study were limited by the low quality of the included studies.

## Conclusion

In summary, by combining the available evidence, we found that JGSQW is safe for the T2DM patients. Compared with hypoglycemic agents alone, combination treatment with JGSQW enhances the effect on glucose metabolism in patients with T2DM. We found no difference in the efficacy of JGSQW alone compared to hypoglycemic agents alone. In terms of lipid metabolism, the current evidence is insufficient and too inconsistent for us to draw firm conclusions, so further studies are needed.

## Data Availability

The original contributions presented in the study are included in the article/Supplementary Material; further inquiries can be directed to the corresponding author.

## Abbreviations

T2DM, type 2 diabetes mellitus; JGSQW, Jīn-Guì Shèn-Qì Wán; DM, diabetes mellitus; TCM, traditional Chinese medicine; MECIR, methodological expectations for conduct, reporting and updating of systematic reviews of intervention; PRISMA, Preferred Reporting Items for Systematic Reviews and Meta-Analyses; AMSTAR 2, A Measurement Tool to Assess Systematic Reviews 2; CENTRAL, Cochrane Central Register of Controlled Trials ; CNKI, China National Knowledge Infrastructure; cqvip, VIP information resource integration service platform; CHiCTR, Chinese Clinical Trial Registry; RCT, randomized controlled trial; HbA1c, glycated hemoglobin; FBG, fasting blood glucose; 2hBG: 2-hour postprandial glucose; HDL-C, high-density lipoprotein cholesterol; LDL-C, low-density lipoprotein cholesterol; TC, total cholesterol; TG: triglyceride; ROB2, version 2 of the Cochrane risk-of-bias tool for randomized trials; MD: mean difference; CI, confidence interval; BMI, body mass index.
