# Supplementary material for: Evidence and Potential Mechanisms of Jin-Gui Shen-Qi Wan as a Treatment for Type 2 Diabetes Mellitus: A Systematic Review and Meta-Analysis
Source: Front Pharmacol. 2021 Sep 6;12:699932. doi: 10.3389/fphar.2021.699932 (PMC8450514; doi:10.3389/fphar.2021.699932)
Supplement: Supplementary file 1 [file DataSheet1.docx]

**Table of Contents**

[1. PRISMA 2020 checklist 4](#_Toc77800544)

[2. AMSTAR 2 Checklist 8](#_Toc77800545)

[3. Search strategies in databases 15](#_Toc77800546)

[CNKI: 15](#_Toc77800547)

[PubMed: 15](#_Toc77800548)

[Wanfang: 15](#_Toc77800549)

[VIP: 16](#_Toc77800550)

[EMBASE: 16](#_Toc77800551)

[Cochrane Library 17](#_Toc77800552)

[CHiCTR 17](#_Toc77800553)

[ClinicalTrials.gov 18](#_Toc77800554)

[4. List of studies excluded after reading full-text and reasons 20](#_Toc77800555)

[Study design: 20](#_Toc77800556)

[Intervention: 20](#_Toc77800557)

[Duplicate: 20](#_Toc77800558)

[Outcome: 20](#_Toc77800559)

[Comparison: 20](#_Toc77800560)

[Awaiting classification: 20](#_Toc77800561)

[References 21](#_Toc77800562)

[5. Supporting information for risk of bias assessment 31](#_Toc77800563)

[Yuting Guo, 2012 31](#_Toc77800564)

[Yu Zhao, 2012 34](#_Toc77800565)

[Bin Jiang, 2020 36](#_Toc77800566)

[Shufang Geng, 2020 38](#_Toc77800567)

[Jingzu Zhang, 2019 41](#_Toc77800568)

[Hongyun Sun, 2018 43](#_Toc77800569)

[Cuirong Hou, 2017 45](#_Toc77800570)

[Lina Chen, 2012 47](#_Toc77800571)

[Xiaoming Yang, 2011 50](#_Toc77800572)

[Zhaoyi Huang, 2010 52](#_Toc77800573)

[Xinyi Zhang, 2011 54](#_Toc77800574)

[Yinzhong Li, 2012 57](#_Toc77800575)

[Yang Xiao, 2016 59](#_Toc77800576)

[Fan Jia, 2016 61](#_Toc77800577)

[6. Supporting information for data analysis 64](#_Toc77800578)

[6.1 Forest plot of HbA1c by combination treatment compared with hypoglycemic agents alone 64](#_Toc77800579)

[6.2 Sensitivity analysis of HbA1c by combination treatment compared with hypoglycemic agents alone 64](#_Toc77800580)

[6.3 Subgroup analysis of HbA1c by combination treatment compared with hypoglycemic agents alone according to ages 64](#_Toc77800581)

[6.4 Further comparisons of combination treatment compared with hypoglycemic agents on HbA1c based on specific medicines 65](#_Toc77800582)

[6.5 Forest plot of effect on HbA1c by JGSQW compared with hypoglycemic agents 65](#_Toc77800583)

[6.6 Forest plot of FBG by combination treatment compared with hypoglycemic agents alone 66](#_Toc77800584)

[6.7 Sensitivity analysis of FBG by combination treatment compared with hypoglycemic agents alone 66](#_Toc77800585)

[6.8 Subgroup analysis of FBG by combination treatment compared with hypoglycemic agents alone according to ages 67](#_Toc77800586)

[6.9 Subgroup analysis of FBG by combination treatment compared with hypoglycemic agents alone according to comorbidity 67](#_Toc77800587)

[6.10 Subgroup analysis of FBG by combination treatment compared with hypoglycemic agents alone according to forms of JGSQW 68](#_Toc77800588)

[6.11 Subgroup analysis of FBG by combination treatment compared with hypoglycemic agents alone according to baseline level of FBG 68](#_Toc77800589)

[6.12 Further comparisons of combination treatment compared with hypoglycemic agents on FBG based on specific medicines 69](#_Toc77800590)

[6.13 Effect on FBG by JGSQW compared with hypoglycemic agents 69](#_Toc77800591)

[6.14 Forest plot of 2hBG by combination treatment compared with hypoglycemic agents alone 70](#_Toc77800592)

[6.15 Sensitivity analysis of 2hBG by combination treatment compared with hypoglycemic agents alone 70](#_Toc77800593)

[6.16 Subgroup analysis of 2hBG by combination treatment compared with hypoglycemic agents alone according to ages 71](#_Toc77800594)

[6.17 Subgroup analysis of 2hBG by combination treatment compared with hypoglycemic agents alone according to comorbidity 72](#_Toc77800595)

[6.18 Subgroup analysis of 2hBG by combination treatment compared with hypoglycemic agents alone according to forms of JGSQW 72](#_Toc77800596)

[6.19 Further comparisons of combination treatment compared with hypoglycemic agents on 2hBG based on specific medicines 73](#_Toc77800597)

[6.20 Forest plot on 2hBG by JGSQW compared with hypoglycemic agents 73](#_Toc77800598)

[6.21 Forest plot of HDL-C by combination treatment compared with hypoglycemic agents alone 74](#_Toc77800599)

[6.22 Forest plot of HDL-C by JGSQW compared with hypoglycemic agents 74](#_Toc77800600)

[6.23 Forest plot of LDL-C by JGSQW compared with Xiaoke Pills 74](#_Toc77800601)

[6.24 Forest plot on TC by combination treatment compared with hypoglycemic agents alone 75](#_Toc77800602)

[6.25 Forest plot of TC by JGSQW compared with hypoglycemic agents 75](#_Toc77800603)

[6.26 Forest plot of TG by combination treatment compared with hypoglycemic agents alone 76](#_Toc77800604)

[6.27 Forest plot of TG by JGSQW compared with hypoglycemic agents 76](#_Toc77800605)

[6.28 Funnel plot of 2hBG 77](#_Toc77800606)

[6.29 Funnel plot of FBG 77](#_Toc77800607)

[7. Phytochemical profile of preparation 77](#_Toc77800608)

[7.1 Phytochemical profile of Rehmannia glutinosa (Gaertn.) DC. Orobanchaceae (*dì huáng*) 78](#_Toc77800609)

[7.2 Phytochemical profile of Dioscorea oppositifolia L. Dioscoreaceae (*huái shān yào*) 78](#_Toc77800610)

[7.3 Phytochemical profile of Cornus officinalis Siebold & Zucc. Cornaceae (*shān zhū yú*) 79](#_Toc77800611)

[7.4 Phytochemical profile of Alisma plantago-aquatica L. Alismataceae (*zé xiè*) 80](#_Toc77800612)

[7.5 Phytochemical profile of Smilax glabra Roxb. Smilacaceae (*fú líng*) 81](#_Toc77800613)

[7.6 Phytochemical profile of Paeonia × suffruticosa Andrews. Paeoniaceae (*mŭ dān pí*) 83](#_Toc77800614)

[7.7 Phytochemical profile of Neolitsea cassia (L.) Kosterm. Lauraceae (*guì zhī*) 84](#_Toc77800615)

[7.8 Phytochemical profile of Aconitum carmichaeli Debeaux. Ranunculaceae (*zhì fù zĭ*) 85](#_Toc77800616)

[7.9 Phytochemical profile of JGSQW 87](#_Toc77800617)

# PRISMA 2020 checklist

| **Section and Topic** | **Item #** | **Checklist item** | **Location where item is reported** |
| --- | --- | --- | --- |
| **TITLE** | | |  |
| Title | 1 | Identify the report as a systematic review. | P1 |
| **ABSTRACT** | | |  |
| Abstract | 2 | See the PRISMA 2020 for Abstracts checklist. | P1 |
| **INTRODUCTION** | | |  |
| Rationale | 3 | Describe the rationale for the review in the context of existing knowledge. | P2 |
| Objectives | 4 | Provide an explicit statement of the objective(s) or question(s) the review addresses. | P3 |
| **METHODS** | | |  |
| Eligibility criteria | 5 | Specify the inclusion and exclusion criteria for the review and how studies were grouped for the syntheses. | P3-4 |
| Information sources | 6 | Specify all databases, registers, websites, organisations, reference lists and other sources searched or consulted to identify studies. Specify the date when each source was last searched or consulted. | P3 |
| Search strategy | 7 | Present the full search strategies for all databases, registers and websites, including any filters and limits used. | Supplementary material |
| Selection process | 8 | Specify the methods used to decide whether a study met the inclusion criteria of the review, including how many reviewers screened each record and each report retrieved, whether they worked independently, and if applicable, details of automation tools used in the process. | P4-5 |
| Data collection process | 9 | Specify the methods used to collect data from reports, including how many reviewers collected data from each report, whether they worked independently, any processes for obtaining or confirming data from study investigators, and if applicable, details of automation tools used in the process. | P5 |
| Data items | 10a | List and define all outcomes for which data were sought. Specify whether all results that were compatible with each outcome domain in each study were sought (e.g. for all measures, time points, analyses), and if not, the methods used to decide which results to collect. | P4, P5 |
|  | 10b | List and define all other variables for which data were sought (e.g. participant and intervention characteristics, funding sources). Describe any assumptions made about any missing or unclear information. | P5 |
| Study risk of bias assessment | 11 | Specify the methods used to assess risk of bias in the included studies, including details of the tool(s) used, how many reviewers assessed each study and whether they worked independently, and if applicable, details of automation tools used in the process. | P5 |
| Effect measures | 12 | Specify for each outcome the effect measure(s) (e.g. risk ratio, mean difference) used in the synthesis or presentation of results. | P5 |
| Synthesis methods | 13a | Describe the processes used to decide which studies were eligible for each synthesis (e.g. tabulating the study intervention characteristics and comparing against the planned groups for each synthesis (item #5)). | P5 |
|  | 13b | Describe any methods required to prepare the data for presentation or synthesis, such as handling of missing summary statistics, or data conversions. | P6 |
|  | 13c | Describe any methods used to tabulate or visually display results of individual studies and syntheses. | P5 |
|  | 13d | Describe any methods used to synthesize results and provide a rationale for the choice(s). If meta-analysis was performed, describe the model(s), method(s) to identify the presence and extent of statistical heterogeneity, and software package(s) used. | P5 |
|  | 13e | Describe any methods used to explore possible causes of heterogeneity among study results (e.g. subgroup analysis, meta-regression). | P5-6 |
|  | 13f | Describe any sensitivity analyses conducted to assess robustness of the synthesized results. | P5 |
| Reporting bias assessment | 14 | Describe any methods used to assess risk of bias due to missing results in a synthesis (arising from reporting biases). | P6 |
| Certainty assessment | 15 | Describe any methods used to assess certainty (or confidence) in the body of evidence for an outcome. | - |
| **RESULTS** | | |  |
| Study selection | 16a | Describe the results of the search and selection process, from the number of records identified in the search to the number of studies included in the review, ideally using a flow diagram. | P6 |
|  | 16b | Cite studies that might appear to meet the inclusion criteria, but which were excluded, and explain why they were excluded. | Supplementary material |
| Study characteristics | 17 | Cite each included study and present its characteristics. | P6-7, table 1 |
| Risk of bias in studies | 18 | Present assessments of risk of bias for each included study. | P7, table 2, table 3 |
| Results of individual studies | 19 | For all outcomes, present, for each study: (a) summary statistics for each group (where appropriate) and (b) an effect estimate and its precision (e.g. confidence/credible interval), ideally using structured tables or plots. | Table 4 |
| Results of syntheses | 20a | For each synthesis, briefly summarise the characteristics and risk of bias among contributing studies. | P7-10 |
|  | 20b | Present results of all statistical syntheses conducted. If meta-analysis was done, present for each the summary estimate and its precision (e.g. confidence/credible interval) and measures of statistical heterogeneity. If comparing groups, describe the direction of the effect. | P7-10 |
|  | 20c | Present results of all investigations of possible causes of heterogeneity among study results. | P7-10 |
|  | 20d | Present results of all sensitivity analyses conducted to assess the robustness of the synthesized results. | P7-10 |
| Reporting biases | 21 | Present assessments of risk of bias due to missing results (arising from reporting biases) for each synthesis assessed. | P10 |
| Certainty of evidence | 22 | Present assessments of certainty (or confidence) in the body of evidence for each outcome assessed. | - |
| **DISCUSSION** | | |  |
| Discussion | 23a | Provide a general interpretation of the results in the context of other evidence. | P11 |
|  | 23b | Discuss any limitations of the evidence included in the review. | P11-12 |
|  | 23c | Discuss any limitations of the review processes used. | P12 |
|  | 23d | Discuss implications of the results for practice, policy, and future research. | P11-12 |
| **OTHER INFORMATION** | | |  |
| Registration and protocol | 24a | Provide registration information for the review, including register name and registration number, or state that the review was not registered. | - |
|  | 24b | Indicate where the review protocol can be accessed, or state that a protocol was not prepared. | - |
|  | 24c | Describe and explain any amendments to information provided at registration or in the protocol. | - |
| Support | 25 | Describe sources of financial or non-financial support for the review, and the role of the funders or sponsors in the review. | P13 |
| Competing interests | 26 | Declare any competing interests of review authors. | P13 |
| Availability of data, code and other materials | 27 | Report which of the following are publicly available and where they can be found: template data collection forms; data extracted from included studies; data used for all analyses; analytic code; any other materials used in the review. | P17 |

*From:*  Page MJ, McKenzie JE, Bossuyt PM, Boutron I, Hoffmann TC, Mulrow CD, et al. The PRISMA 2020 statement: an updated guideline for reporting systematic reviews. BMJ 2021;372:n71. doi: 10.1136/bmj.n71

For more information, visit: <http://www.prisma-statement.org/>

| AMSTAR 2 Checklist |
| --- |
| \|  \| \| --- \| \| **1. Did the research questions and inclusion criteria for the review include the components of PICO?** \| \| \| For Yes: \| Optional (recommended) \|  \| \| --- \| --- \| --- \| \|  Population \|  Timeframe for follow up \|  Yes   No \| \|  Intervention \|  \| \|  Comparator group \|  \| \|  Outcome \|  \| \| \|  \| |
| \| **2. Did the report of the review contain an explicit statement that the review methods were established prior to the conduct of the review and did the report justify any significant deviations from the protocol?** \| \| --- \| \| \| For Partial Yes: The authors state that they had a written protocol or guide that included ALL the following: \| For Yes: As for partial yes, plus the protocol should be registered and should also have specified: \|  \| \| --- \| --- \| --- \| \|  review question(s) \|  a meta-analysis/synthesis plan, if appropriate, and \|  Yes   Partial Yes   No \| \|  a search strategy \|  a plan for investigating causes of heterogeneity \| \|  inclusion/exclusion criteria \|  a plan for investigating causes of heterogeneity \| \|  a risk of bias assessment \|  \| \| |
|  |
| \| **3. Did the review authors explain their selection of the study designs for inclusion in the review?** \| \| --- \| \| \| For Yes, the review should satisfy ONE of the following: \|  \| \| --- \| --- \| \|  Explanation for including only RCTs \|  Yes   No \| \|  OR Explanation for including only NRSI \| \|  OR Explanation for including both RCTs and NRSI \| \| |
|  |
| \| **4. Did the review authors use a comprehensive literature search strategy?** \| \| --- \| \| \| For Partial Yes (all the following): \| For Yes, should also have (all the following): \|  \| \| --- \| --- \| --- \| \|  searched at least 2 databases (relevant to research question) \|  searched the reference lists / bibliographies of included studies \|  Yes   Partial Yes   No \| \|  provided key word and/or search strategy \|  searched trial/study registries \| \|  justified publication restrictions (e.g. language) \|  included/consulted content experts in the field \| \|  \|  where relevant, searched for grey literature \| \|  \|  conducted search within 24 months of completion of the review \| \| |
|  |
| \| **5. Did the review authors perform study selection in duplicate?** \| \| --- \| \| \| For Yes, either ONE of the following: \|  \| \| --- \| --- \| \|  at least two reviewers independently agreed on selection of eligible studies and achieved consensus on which studies to include \|  Yes   No \| \|  OR two reviewers selected a sample of eligible studies and achieved good agreement (at least 80 percent), with the remainder selected by one reviewer. \| \| |
|  |
| \| **6. Did the review authors perform data extraction in duplicate?** \| \| --- \| \| \| For Yes, either ONE of the following: \|  \| \| --- \| --- \| \|  at least two reviewers achieved consensus on which data to extract from included studies \|  Yes   No \| \|  OR two reviewers extracted data from a sample of eligible studies and achieved good agreement (at least 80 percent), with the remainder extracted by one reviewer. \| \| |
|  |
| \| **7. Did the review authors provide a list of excluded studies and justify the exclusions?** \| \| --- \| \| \| For Partial Yes: \| For Yes, must also have: \|  \| \| --- \| --- \| --- \| \| provided a list of all potentially relevant studies that were read in full-text form but excluded from the review \| Justified the exclusion from the review of each potentially relevant study \|  Yes   Partial Yes   No \| \| |
|  |
| \| **8. Did the review authors describe the included studies in adequate detail?** \| \| --- \| \| \| For Partial Yes (ALL the following): \| For Yes, should also have ALL the following: \|  \| \| --- \| --- \| --- \| \|  described populations \|  described population in detail \|  Yes   Partial Yes   No \| \|  described interventions \|  described intervention in detail (including doses where relevant) \| \|  described comparators \|  described comparator in detail (including doses where relevant) \| \|  described outcomes \|  described study’s setting \| \|  described research designs \|  timeframe for follow-up \| \| |
|  |
| \| **9. Did the review authors use a satisfactory technique for assessing the risk of bias (RoB) in individual studies that were included in the review?** \| \| --- \| \| \| **RCTs** \|  \|  \| \| --- \| --- \| --- \| \| For Partial Yes, must have assessed RoB from \| For Yes, must also have assessed RoB from: \|  \| \|  unconcealed allocation, and \|  allocation sequence that was not truly random, and \|  Yes   Partial Yes   No   Includes only NRSI \| \|  lack of blinding of patients and assessors when assessing outcomes (unnecessary for objective outcomes such as all-cause mortality) \|  selection of the reported result from among multiple measurements or analyses of a specified outcome \| \| **NRSI** \|  \|  \| \| For Partial Yes, must have assessed RoB: \| For Yes, must also have assessed RoB: \|  \| \|  from confounding, and \|  methods used to ascertain exposures and outcomes, and \|  Yes   Partial Yes   No   Includes only RCTs \| \|  from selection bias \|  selection of the reported result from among multiple measurements or analyses of a specified outcome \| \| |
|  |
| \| **10. Did the review authors report on the sources of funding for the studies included in the review?** \| \| --- \| \| \| For Yes \|  \| \| --- \| --- \| \|  Must have reported on the sources of funding for individual studies included in the review. Note: Reporting that the reviewers looked for this information but it was not reported by study authors also qualifies \|  Yes   No \| \| |
|  |
| \| **11. If meta-analysis was performed did the review authors use appropriate methods for statistical combination of results?** \| \| --- \| \| \| **RCTs** \|  \| \| --- \| --- \| \| For Yes: \|  \| \|  The authors justified combining the data in a meta-analysis \|  Yes   No   No meta-analysis conducted \| \|  AND they used an appropriate weighted technique to combine study results and adjusted for heterogeneity if present. \| \|  AND investigated the causes of any heterogeneity \| \|  \|  \| \| **For NRSI** For Yes: \|  \| \|  The authors justified combining the data in a meta-analysis \|  Yes   No   No meta-analysis conducted \| \|  AND they used an appropriate weighted technique to combine study results, adjusting for heterogeneity if present \| \|  AND they statistically combined effect estimates from NRSI that were adjusted for confounding, rather than combining raw data, or justified combining raw data when adjusted effect estimates were not available \| \|  AND they reported separate summary estimates for RCTs and NRSI separately when both were included in the review \| \| |
|  |
| \| **12. If meta-analysis was performed, did the review authors assess the potential impact of RoB in individual studies on the results of the meta-analysis or other evidence synthesis?** \| \| --- \| \| \| For Yes: \|  \| \| --- \| --- \| \|  included only low risk of bias RCTs \|  Yes  No   No meta-analysis conducted \| \|  OR, if the pooled estimate was based on RCTs and/or NRSI at variable RoB, the authors performed analyses to investigate possible impact of RoB on summary estimates of effect. \| \| |
|  |
| \| **13. Did the review authors account for RoB in individual studies when interpreting/ discussing the results of the review?** \| \| --- \| \| \| For Yes: \|  \| \| --- \| --- \| \|  included only low risk of bias RCTs \|  Yes   No \| \|  OR, if RCTs with moderate or high RoB, or NRSI were included the review provided a discussion of the likely impact of RoB on the results \|  \| \| |
|  |
| \| **14. Did the review authors provide a satisfactory explanation for, and discussion of, any heterogeneity observed in the results of the review?** \| \| --- \| \| \| For Yes: \|  \| \| --- \| --- \| \| There was no significant heterogeneity in the results \| Yes   No \| \| OR if heterogeneity was present the authors performed an investigation of sources of any heterogeneity in the results and discussed the impact of this on the results of the review \|  \| \| |
|  |
| \| **15. If they performed quantitative synthesis did the review authors carry out an adequate investigation of publication bias (small study bias) and discuss its likely impact on the results of the review?** \| \| --- \| \| \| For Yes: \|  \| \| --- \| --- \| \| performed graphical or statistical tests for publication bias and discussed the likelihood and magnitude of impact of publication bias \| Yes  No  No meta-analysis conducted \| \| |
|  |
| \| **16. Did the review authors report any potential sources of conflict of interest, including any funding they received for conducting the review?** \| \| --- \| \| \| For Yes: \|  \| \| --- \| --- \| \| The authors reported no competing interests OR \| Yes   No \| \| The authors described their funding sources and how they managed potential conflicts of interest \| \| |
|  |
| To cite this tool: Shea BJ, Reeves BC, Wells G, Thuku M, Hamel C, Moran J, Moher D, Tugwell P, Welch V, Kristjansson E, Henry DA. AMSTAR 2: a critical appraisal tool for systematic reviews that include randomised or non-randomised studies of healthcare interventions, or both. BMJ. 2017 Sep 21;358:j4008. |

# Search strategies in databases

## CNKI:

The database search in CNKI was carried out on February 1, 2021, and a total of 482 literatures were found.
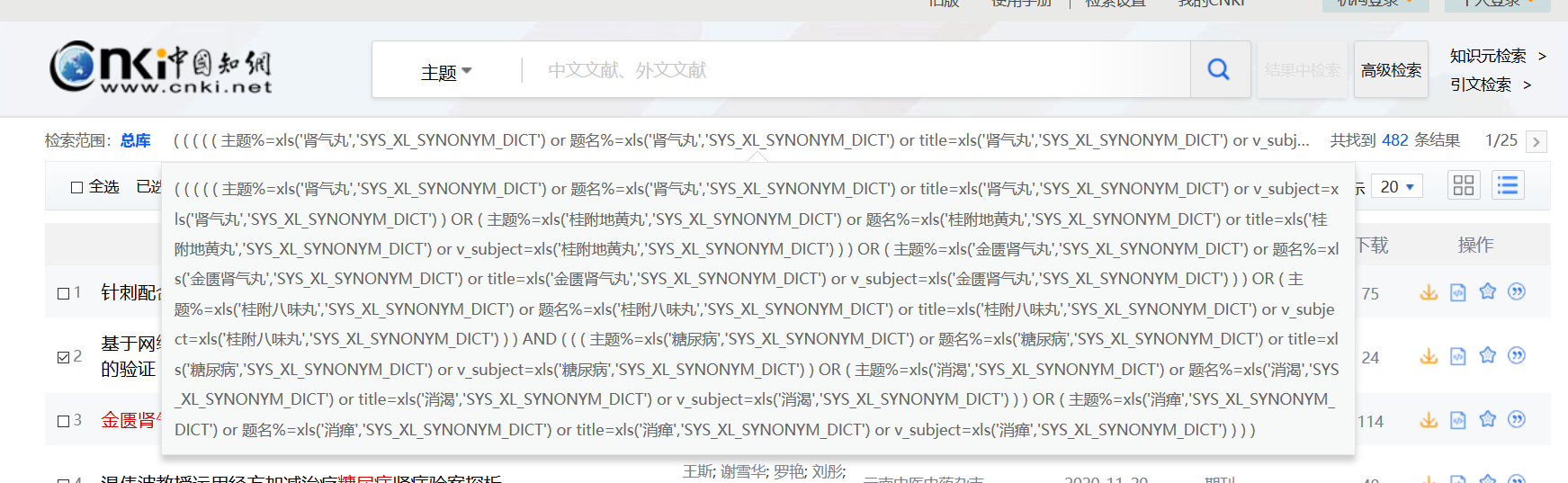


## PubMed:

The database search in PubMed was carried out on February 1, 2021, and a total of 17 literatures were found.


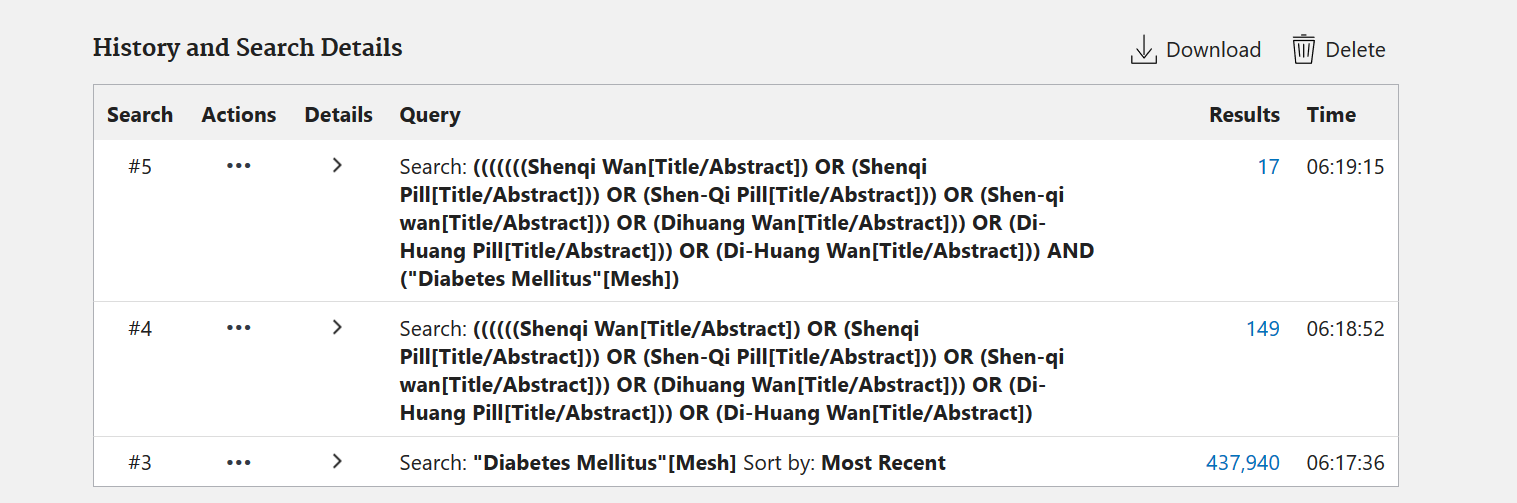


## Wanfang:

The database search in Wanfang was carried out on February 1, 2021, and a total of 523 literatures were found.


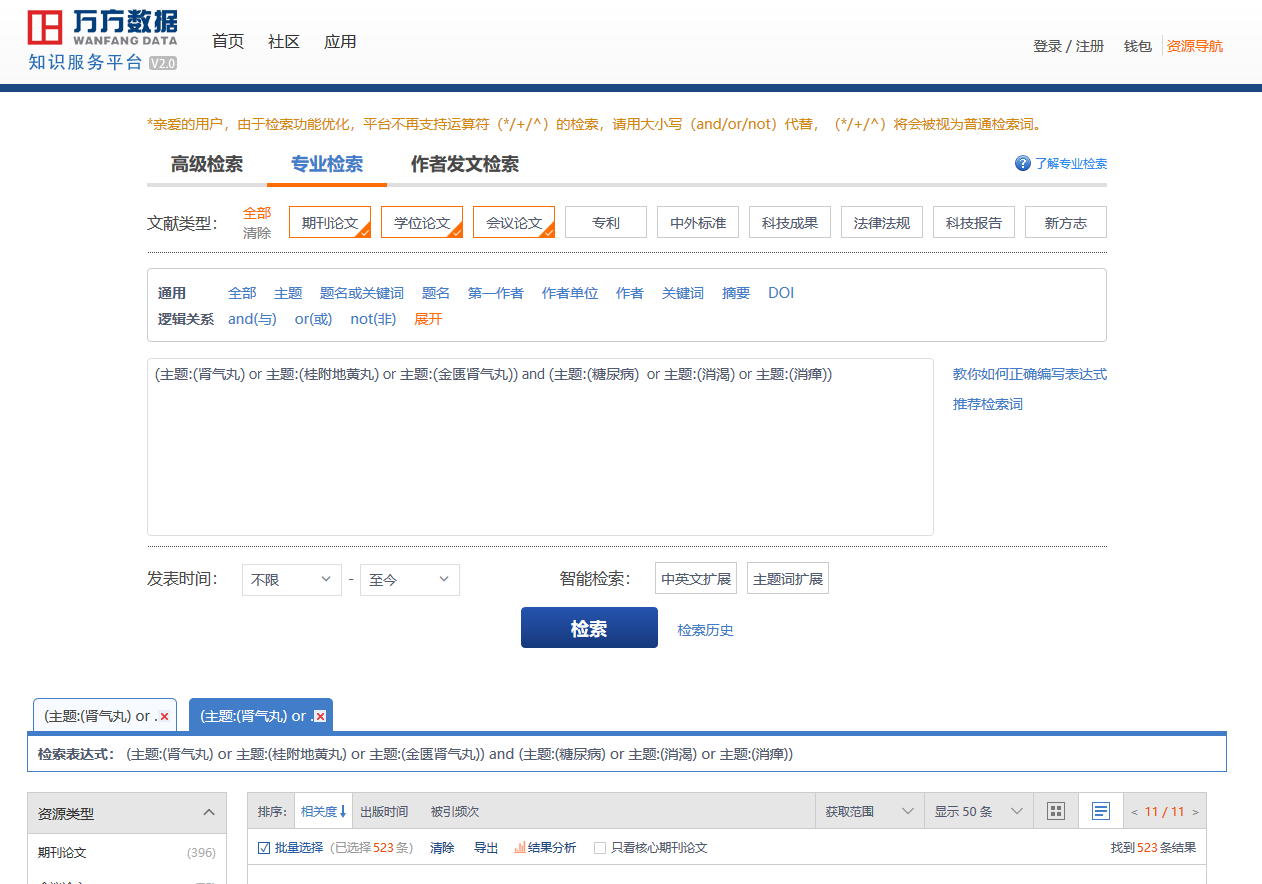


## VIP:

The database search in VIP was carried out on February 1, 2021, and a total of 218 literatures were found.


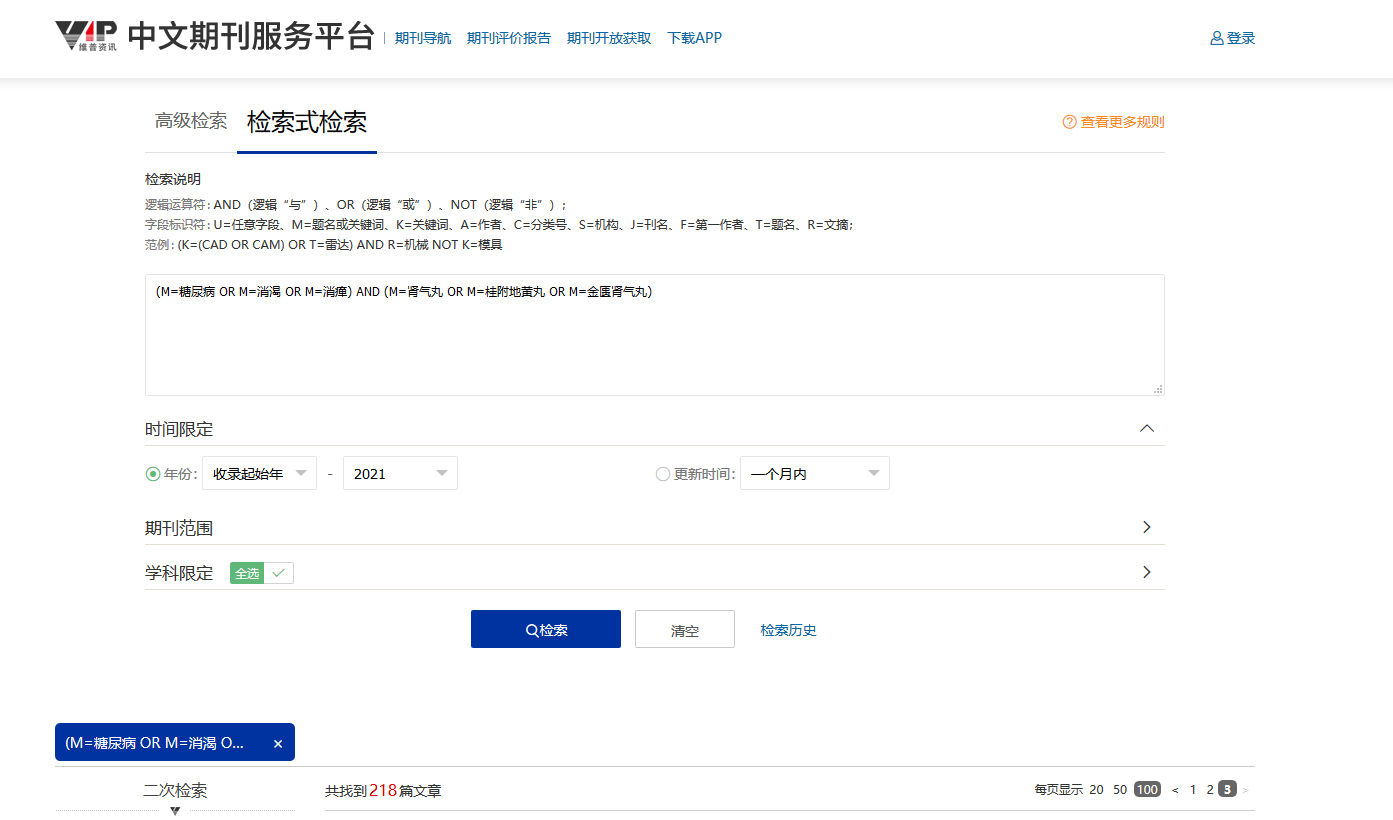


## EMBASE:

The database search in EMBASE was carried out on February 1, 2021, and a total of 1 literature were found.


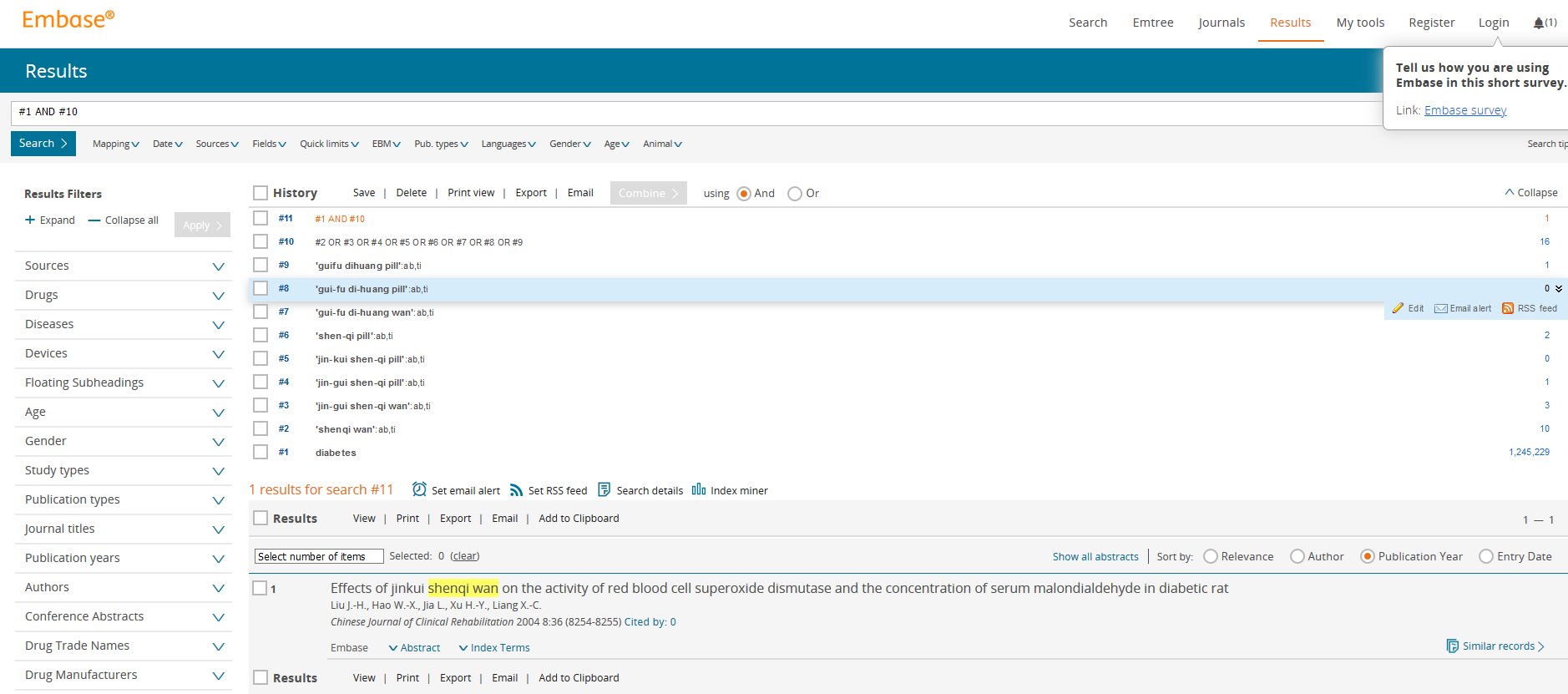


## Cochrane Library

The database search in EMBASE was carried out on February 1, 2021, and a total of 2 literatures were found.


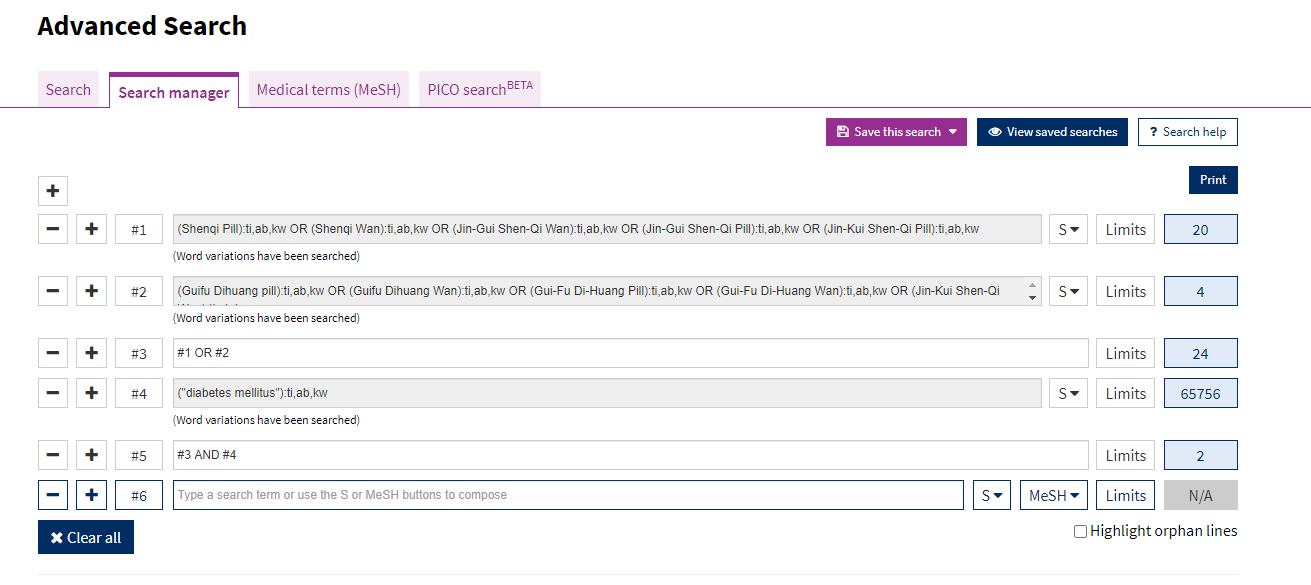


## CHiCTR

The database search in CHiCTR was carried out on February 1, 2021, and a total of 5 literatures were found, 2 of which were about T2DM.


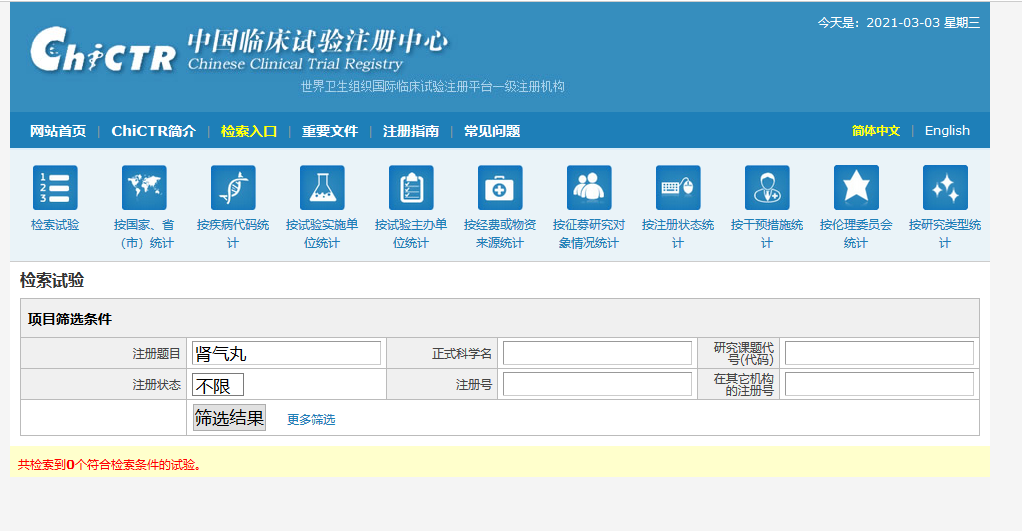


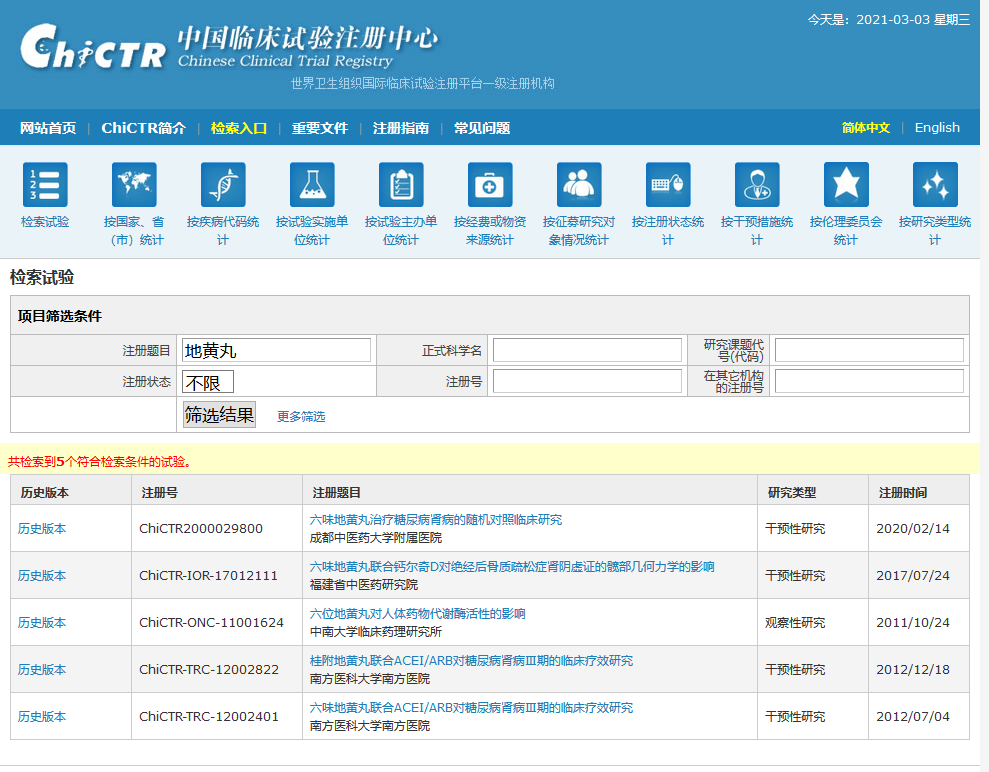


## ClinicalTrials.gov

The database search in ClinicalTrials.gov was carried out on February 1, 2021, and no literature was found.


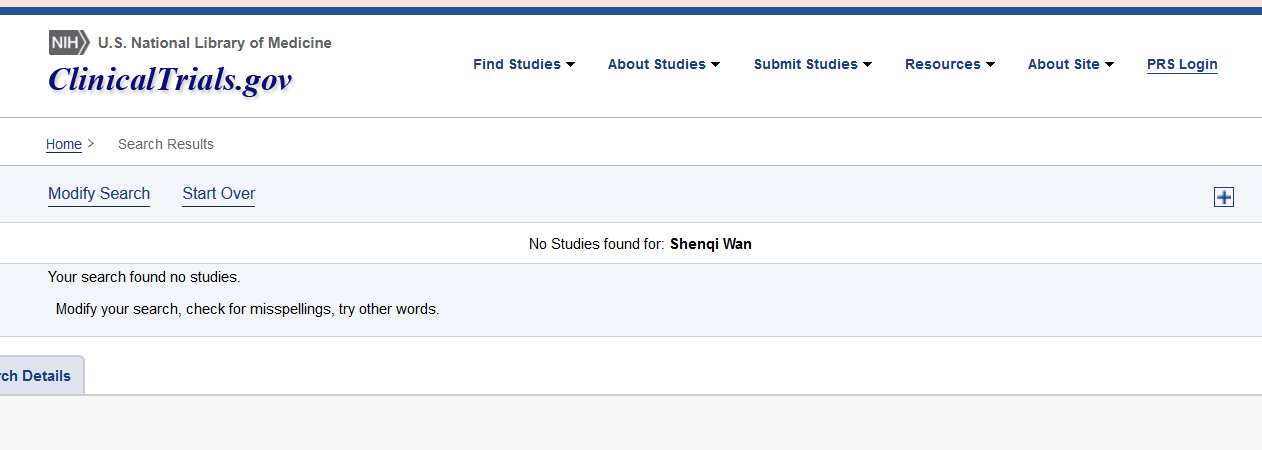


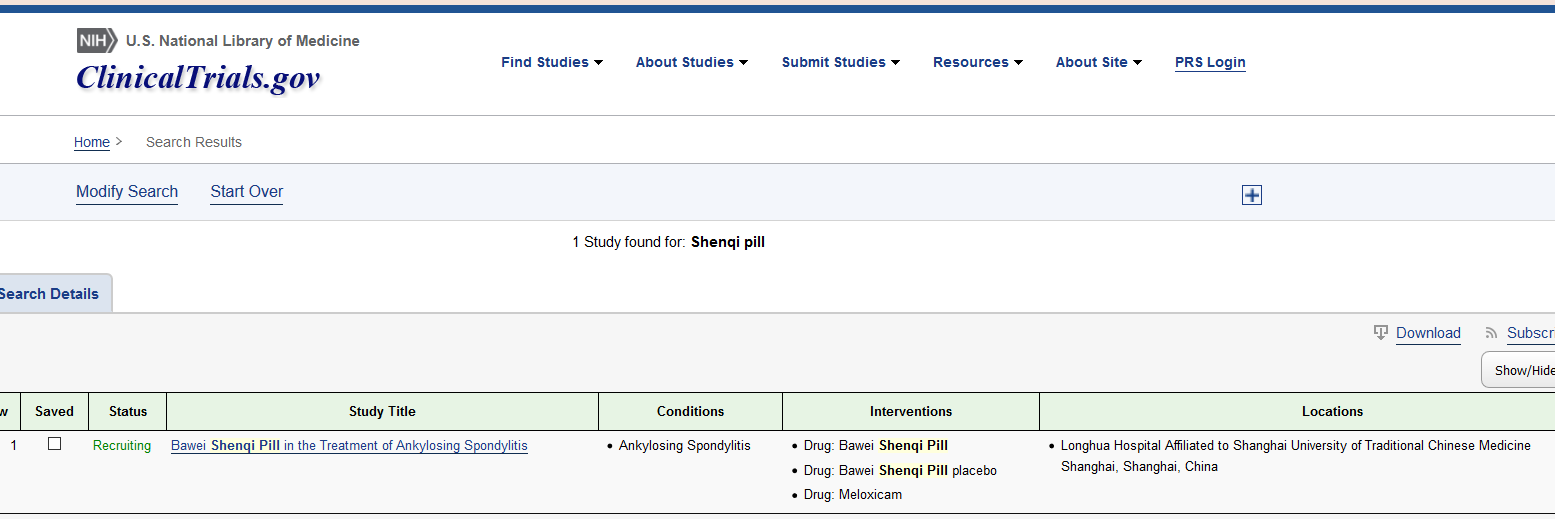


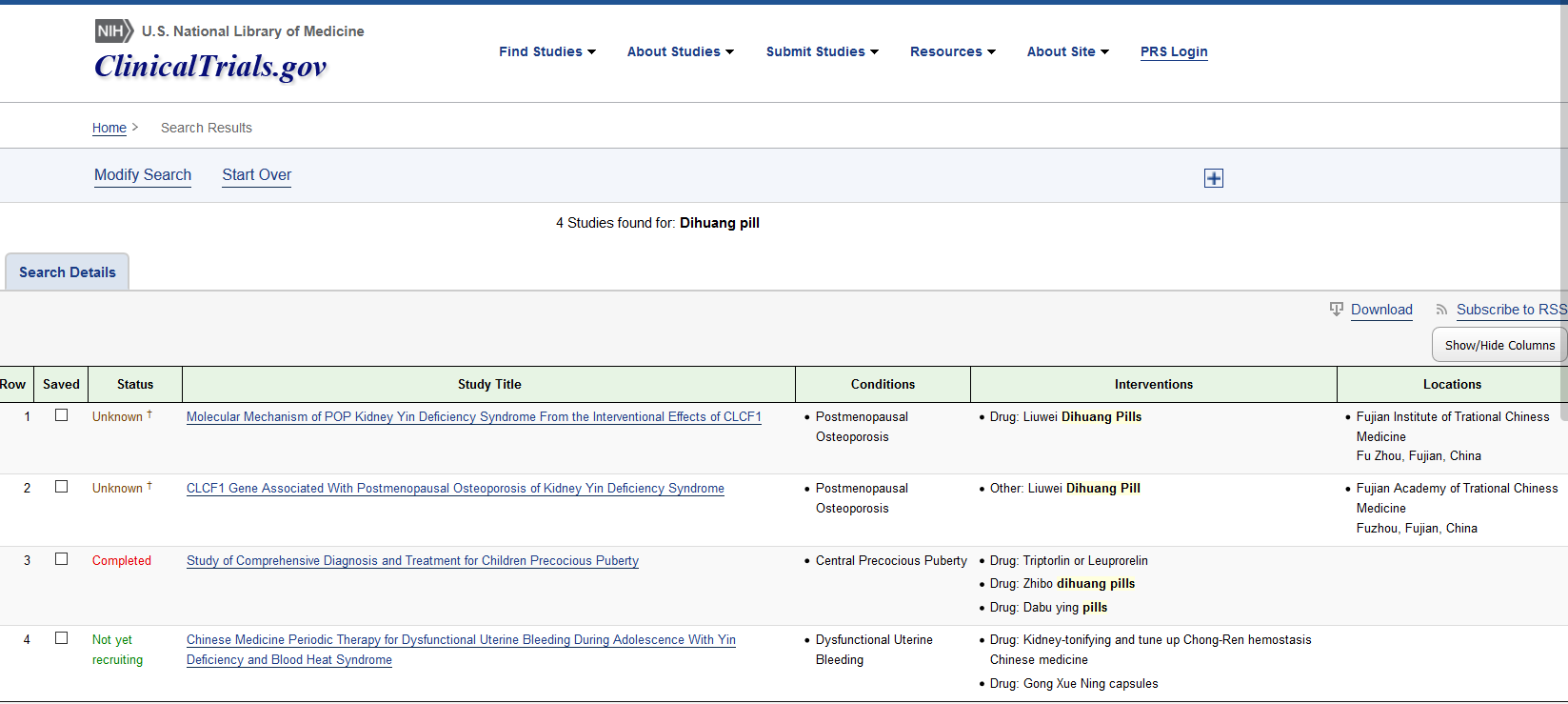


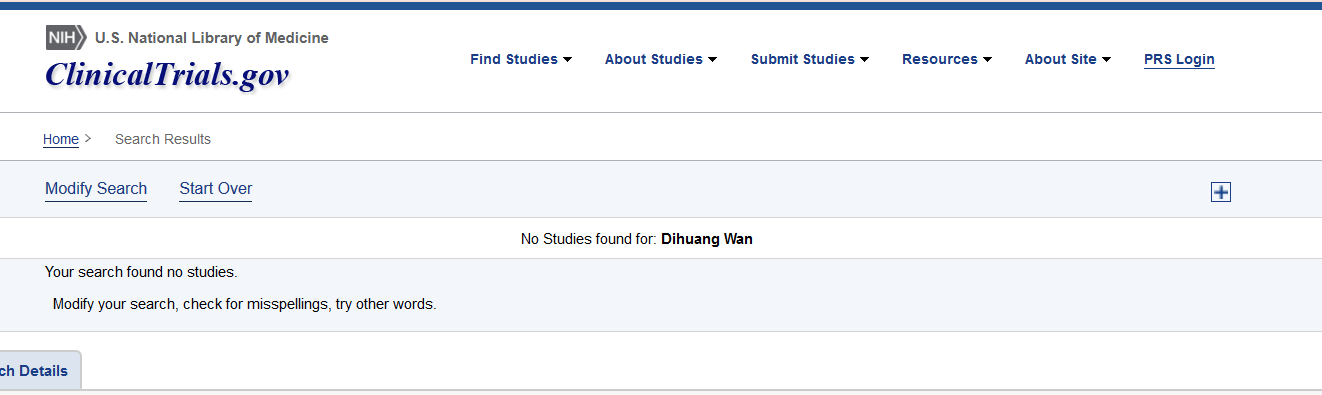


# List of studies excluded after reading full-text and reasons

## Study design:

The following studies were excluded because they are not RCTs (Heqing and Jun 1995; Yi et al. 1996; Yuexi 2010; Dehua 2004; Ming 2003; Mingjiong 2014).

## Intervention:

These studies were excluded because of inappropriate interventions, mainly because of the use of a modified JGSQW (Aihua 2015; Caijing 2018; Fanyan 2019; Fengjuan 2015; Fushu 2012; Hongyu et al. 2014a; Hongyu et al. 2014b; Hongzhuan 2013; Jianguang 2014; Jianjun 2016; Jianmin 2018; Jianying 2016b; Ke 2019; Kun 2019; Le 2012; Li et al. 2011; Lin 2011; Linghong 2018; Ming et al. 2015; Peipei 2016; Richeng 2020; Songjia 2018; Songtao 2000; Tongyuan 2019; Xin 2011; Yae 2009; Yihui 2019; Ying and Shigang 2020; Yongwei 2012; Yuan 2018; Zheng 2019; Zhengqi 2013).

## Duplicate:

The following studies were excluded because they were duplicate articles (Heqing and Jun 1995; Songtao 2000).

## Outcome:

These studies were excluded because they had no available outcome (Jianying 2016a; Jun 2019; Na and Dexian 2011; Qing and Yubo 2014; Shijie 2010; Weiqi 2013; Wen and Wenlan 2019; Yanli 2016; Yanping 2015; Zexiao et al. 2005; Rong and Wei 2006).

## Comparison:

This study was excluded because inappropriate comparison (Shaoju et al. 2017).

## Awaiting classification:

These studies were assessed as awaiting classification because they lack enough information (Guohua 2014; Hongyan 2019).

## References

Aihua, Yang. (2015): Effect of Jingui Shenqi Pill on type 2 diabetes mellitus with deficiency of both yin and Yang. MS. Guangzhou University of traditional Chinese Medicine.

Caijing, Li. (2018): Study on the clinical value of Jinkuishenqi Pills in the treatment of diabetic nephropathy. In *The New World of Diabetes* 21 (01), pp. 180–182.

Dehua, Liu. (2004): Clinical observation on 62 cases of type 2 diabetes mellitus of yin yang deficiency treated by Jingui Shenqi Pill. In *New traditional Chinese medicine* (07), pp. 31–32.

Fanyan, Meng. (2019): Clinical observation on the treatment of type 2 diabetes mellitus (yin yang deficiency syndrome) with modified Jingui Shenqi Pill. MS. Changchun University of traditional Chinese Medicine.

Fengjuan, Shang. (2015): Clinical study on the treatment of diabetic neurogenic bladder with Jingui Shenqi Pill. MS. Shandong University of traditional Chinese Medicine.

Fushu, Xie. (2012): Observation on the curative effect of Jinkui Shenqi Pill on 106 cases of diabetes mellitus. In *China’s health industry* 9 (13), p. 159.

Guohua, Ding (2014): Clinical analysis of Jingui Shenqi Pill in the treatment of type 2 diabetes Clinical analysis of Jingui Shenqi Pill in the treatment of type 2 diabetes mellitus. In *Modern distance education of traditional Chinese medicine in China* 12 (17), pp. 103–104.

Heqing, Huang; Jun, Huang. (1995): Clinical observation on 38 cases of kidney qi deficiency type 2 diabetes treated with modified Jingui Shenqi Pill. In *Hubei Journal of traditional Chinese Medicine* (01), pp. 19–20.

Hongyan, Chen (2019): Therapeutic Effects of Jingui Shenqi Pills Combined with Western Medicine in the Treatment of Diabetes Mellitus Complicated with Edema. In *CHINA JOURNAL OF PHARMACEUTICAL ECONOMI* 14 (02), pp. 66–69.

Hongyu, Ji; Hebin, Li; Jinjin Zhang; Kewei, Zhang. (2014a): Clinical observation on the treatment of diabetic nephropathy stage IV with modified Jingui Shenqi Pill. In *JOURNAL OF NEW CHINESE MEDICINE* 46 (09), pp. 72–73.

Hongyu, Ji; Zhonghua, Zheng; Hebin, Li; Jinjin Zhang; Kewei, Zhang. (2014b): Clinical observation of Jinkui Shenqi Pill in the treatment of stage Ⅳ Diabetic Nephropathy. In *JOURNAL OF NEW CHINESE MEDICINE* 046 (009), pp. 72–73.

Hongzhuan, Wu. (2013): Clinical observation of Jinkui Shenqi Pill in the treatment of type 2 diabetes mellitus. In *Pharmacology and clinic of traditional Chinese Medicine* 29 (03), pp. 191–193.

Jianguang, Wu. (2014): Observation on therapeutic effect of Jingui Shenqi Pill on diabetes. In *Shaanxi traditional Chinese Medicine* 35 (11), pp. 1515–1516.

Jianjun, He. (2016): Effect of Jinkui Shenqi Pill on Diabetic Nephropathy with Yang deficiency of spleen and Kidney and its effect on Renal function. In *Journal of Sichuan of Traditional Chinese Medicine* 34 (06), pp. 84–86.

Jianmin, He. (2018): Analysis of Clinical effect of modified Jinkui Shenqi Pill in the treatment of Diabetes. In *Clinical research of traditional Chinese medicine* 10 (04), pp. 95–96.

Jianying, Wu. (2016a): Clinical effect of Jingui Shenqi Pill on stage Ⅲ diabetic nephropathy. In *Modern health care* (06), p. 170.

Jianying, Wu. (2016b): Clinical observation on treating diabetic nephropathy with modified Jingui Shenqi Pill. MS. Shanxi College of traditional Chinese Medicine.

Jun, Wang. (2019): Effect of valsartan combined with Jingui Shenqi Pill on diabetic erectile dysfunction. In *Electronic Journal of cardiovascular disease of integrated traditional Chinese and Western Medicine* 7 (04), pp. 17–18.

Ke, Jin. (2019): Clinical Observation on the Modified Jinkui Shenqi Pill in the Treatment of Diabetic Nephropathy with Spleen and Kidney Yang Deficiency. In *Practical integration of traditional Chinese and western medicine* 19 (04), pp. 21–23.

Kun, Xia. (2019): Clinical efficacy of Jingui Shenqi Pill in the treatment of diabetic nephropathy. In *A study on the health of women at home and abroad* (07), 39+82.

Le, Zhang. (2012): Study on the clinical efficacy of Liou Wei/Gui Fu Di Huang Wan in DN stage III. MS. Nanfang Medical University.

Li, Liu; Wenfu, Cao; Meiyu, Luo. (2011): Treatment of 26 Cases of Diabetic Peripheral Neuropathy with Jinkuishenqi Pill and Xuesaitong. In *Zunyi Technology* (4), pp. 34–35.

Lin, Wang. (2011): 68 cases of Diabetes treated with Jinkui Shenqi Pill. In *Practical clinical integration of traditional Chinese and western medicine* 11 (02), pp. 57–58.

Linghong, Zhang. (2018): Effect of Jinkui Shenqi Pill on Diabetic Nephropathy with Spleen and Kidney Yang Deficiency and Its Effect on Renal Function. In *Electronic Journal of Cardiovascular Diseases of Integrated Traditional Chinese and Western Medicine* 6 (02), pp. 169–170.

Ming, Deng; Shuiyan, Zhang; Yiheng, Lu. (2015): Clinical observation of Jinkui Shenqi Pill combined with Metformin in the treatment of senile Type 2 Diabetes Mellitus. In *Strait pharmacy* 27 (05), pp. 118–120.

Ming, Zhou. (2003): Clinical study on Jingui Shenqi Pill in treating Jingui Shenqi Pill syndrome. MS. Beijing University of Traditional Chinese Medicine. Available online at <http://www.wanfangdata.com.cn/details/detail.do?_type=degree&id=Y546514>.

Mingjiong, Wang. (2014): Clinical effect analysis of Jingui Shenqi Pill in the treatment of type 2 diabetes mellitus complicated with hyperlipidemia. In *Medical Aesthetics and Cosmetology* 12.

Na, Yang; Dexian, Zhang. (2011): Clinical observation of Jinkui Shenqi Pill Combined with Lotensin in treating diabetic nephropathy of yin yang deficiency type. In *Journal of Shandong University of traditional Chinese Medicine* 35 (03), pp. 232–234.

Peipei, Sun. (2016): Clinical comparative study of modified Jingui Shenqi Pill and Jingui Shenqi Pill in the treatment of stage IV diabetic nephropathy. MS. Shandong University of traditional Chinese Medicine.

Qing, Li; Yubo, Han. (2014): Clinical study of Jingui Shenqi Pill Combined with Mecca in the treatment of stage Ⅲ diabetic nephropathy. In *Guangming traditional Chinese Medicine* 29 (03), pp. 576–578.

Richeng, Yu. (2020): Observation on the effect of Jiawei Shenqi Pill combined with Anti-VEGF drugs in the treatment of Diabetic Retinopathy with deficiency of spleen and Kidney. In *Chinese medical innovation* 17 (23), pp. 73–77.

Rong, Lin; Wei, Li. (2006): 21 cases of diabetic neurogenic bladder treated by Jingui Shenqi Pill Practical Journal of internal medicine of traditional Chinese Medicine (03), p. 260.

Shaoju, Chang; Xianjie, Meng; Li, Li; Lihui, Cao; Hui, Li. (2017): Study on Effect of Serum Hcy，Fibrinogen and Inflammatory Cytokines in Patients with Type 2 Diabetes with Cardiac and Cerebral Vascular Diseases by Sitagliptin and Jinkui Shenqi Pill. In *Liaoning Journal of traditional Chinese Medicine* 44 (04), pp. 800–803.

Shijie, Sun. (2010): Clinical observation of Jingui Shenqi Pill Combined with western medicine in the treatment of stage IV diabetic nephropathy. In *Shaanxi traditional Chinese Medicine* 31 (08), pp. 960–962.

Songjia, Ru. (2018): Effect of Jinkui Shenqi Pill on Diabetic Nephropathy of Spleen and Kidney Yang Deficiency and Its Effect on Renal Function. In *Heilongjiang Medicine* 31 (02), pp. 327–329.

Songtao, Wu. (2000): Treatment of 58 cases of Diabetes with Jiawei Shenqi Pill combined with Western Medicine—A control study of 52 cases treated with Fuyou Jiangtang. In *Journal of Zhejiang Traditional Chinese Medicine* (05), p. 12.

Tongyuan, Lei. (2019): Clinical effect analysis of Jinkui Shenqi Pill in the treatment of diabetes. In *Health required* 000 (030), p. 48.

Weiqi, Zhu. (2013): Treatment of 18 cases of type 2 diabetes mellitus complicated with hyperlipidemia by Jingui Shenqi Pill. In *Modern distance education of traditional Chinese medicine in China* 11 (15), pp. 96–97.

Wen, Chen; Wenlan, Wang. (2019): Effect of Jingui Shenqi Pill on diabetic nephropathy of spleen kidney yang deficiency type and its influence on renal function. In *Oriental medicated food* 000 (013), pp. 17–18.

Xin, Jin. (2011): Clinical observation on 50 cases of Diabetes treated with Jinkui Shenqi Pill. In *CHINA MODERN DOCTOR* 49 (12), 60+66.

Yae, Lu. (2009): Treatment of 60 cases of diabetic nephropathy with Jinkui Shenqi Pill Modified with Western Medicine. In *Journal of Shaanxi College of Traditional Chinese Medicine* 32 (05), pp. 29–31.

Yanli, Gao (Ed.) (2016): Clinical effect of Jingui Shenqi Pill Combined with western medicine in the treatment of stage IV diabetic nephropathy. Conference of “emergency medicine clinical research association”.

Yanping, Hu. (2015): Clinical effect of Jinkui Shenqi Pill Combined with western medicine on stage IV diabetic nephropathy. In *New world of diabetes* (08), p. 36.

Yi, Cao; Ying, Jiangl; Qingping, Zhang. (1996): 18 cases of diabetic nephropathy treated with Jingui Shenqi Pill. In *Shaanxi traditional Chinese Medicine* (08), p. 363.

Yihui, Yan (2019): Treatment of diabetic nephropathy of spleen and kidney yang deficiency type by Jingui Shenqi Pill Combined with Huoxue Huayu method. In *Diabetes education (first ten days)* 016 (005), 49,51.

Ying, Yuan; Shigang, Nie. (2020): Effect of Jinkui Shenqi Pill on Blood Sugar and Renal Function in Diabetic Nephropathy with Deficiency of Yin and Yang. In *Modern Chinese Medicine* 40 (05), 70-72+76.

Yongwei, Jiao. (2012): Observation on Therapeutic Effect of Jinkuishenqi Pill on 53 Cases of Diabetes. In *Medical theory and practice* 25 (01), pp. 47–48.

Yuan, Cheng. (2018): Clinical observation of Nuohelong combined with Jiawei Shenqi Pill in the treatment of Type 2 Diabetes Mellitus with deficiency of both Yin and Yang. MD. Zhejiang University of traditional Chinese Medicine.

Yuexi, Xu. (2010): Therapeutic effect of Jingui Shenqi Pill on diabetes mellitus. In *Chinese Journal of health nutrition: Journal of clinical medicine* 000 (002), p. 58.

Zexiao, Deng; Ruyi, Peng; Huiying, Chen; Jieying, Deng; Ping, Cai; Luolan, Xin; Huixin, Huang. (2005): Clinical observation on 17 cases of diabetic foot treated with povidone iodine ointment and Jingui Shenqi Pill. In *Modern hospital* (04), pp. 66–68.

Zheng, Wang. (2019): Effect observation of modified Jingui Shenqi Pill on diabetic nephropathy of spleen kidney yang deficiency type. In *Heilongjiang traditional Chinese Medicine* 48 (05), pp. 61–62.

Zhengqi, Han. (2013): Report of 84 cases of Diabetic Nephropathy in IV stage treated with Jinkui Shenqi Pill. In *Clinical Journal of traditional Chinese Medicine* 25 (06), p. 524.

# Supporting information for risk of bias assessment

## Yuting Guo, 2012

**Bias arising from the randomization process**

| Signaling question | Response | Supporting information |
| --- | --- | --- |
| 1.1 Was the allocation sequence random ? | NI | The author did not report random number sequence generation in detail. |
| 1.2 Was the allocation sequence concealed until participants were enrolled and assigned to interventions ? | NI | The author did not report allocation sequence concealment in detail. |
| 1.3 Did baseline differences between intervention groups suggest a problem with the randomization process? | N | The baseline was balanced. |

**bias due to deviations from the intended interventions (effect of assignment to intervention)**

| Signaling question | Response | Supporting information |
| --- | --- | --- |
| 2.1. Were participants aware of their assigned intervention during the trial? | Y | Blinding was not used. |
| 2.2. Were carers and people delivering the interventions aware of participants' assigned intervention during the trial? | Y | Blinding was not used. |
| 2.3. If Y/PY/NI to 2.1 or 2.2: Were there deviations from the intended intervention that arose because of the trial context? | NI | No enough information. |
| 2.4 If Y/PY to 2.3: Were these deviations likely to have affected the outcome? | NA | - |
| 2.5. If Y/PY/NI to 2.4: Were these deviations from intended intervention balanced between groups? | NA | - |
| 2.6 Was an appropriate analysis used to estimate the effect of assignment to intervention? | N | ITT analysis was not used. |
| 2.7 If N/PN/NI to 2.6: Was there potential for a substantial impact (on the result) of the failure to analyze participants in the group to which they were randomized? | NI | No enough information. |

**Bias due to missing outcome data**

| Signaling question | Response | Supporting information |
| --- | --- | --- |
| 3.1 Were data for this outcome available for all, or nearly all, participants randomized? | Y | Authors reported data for almost all subjects participating in randomization. |
| 3.2 If N/PN/NI to 3.1: Is there evidence that the result was not biased by missing outcome data? | NA |  |
| 3.3 If N/PN to 3.2: Could missingness in the outcome depend on its true value? | NA |  |
| 3.4 If Y/PY/NI to 3.3: Is it likely that missingness in the outcome depended on its true value? | NA | . |

**bias in measurement of the outcome**

| Signaling question | Response | Supporting information |
| --- | --- | --- |
| 4.1 Was the method of measuring the outcome inappropriate? | N | The method was appropriate. |
| 4.2 Could measurement or ascertainment of the outcome have differed between intervention groups? | N | There was no difference between groups. |
| 4.3 If N/PN/NI to 4.1 and 4.2: Were outcome assessors aware of the intervention received by study participants? | Y | Blinding was not used. |
| 4.4 If Y/PY/NI to 4.3: Could assessment of the outcome have been influenced by knowledge of intervention received? | N | The outcome involves no judgement. |
| 4.5 If Y/PY/NI to 4.4: Is it likely that assessment of the outcome was influenced by knowledge of intervention received? | NA |  |

**bias in selection of the reported result**

| Signaling question | Response | Supporting information |
| --- | --- | --- |
| 5.1 Were the data that produced this result analyzed in accordance with a pre-specified analysis plan that was finalized before unblinded outcome data were available for analysis? | NI | Protocol was not available. |
| s the numerical result being assessed likely to have been selected, on the basis of the results, from... |  |  |
| 5.2. ... multiple eligible outcome measurements (e.g. scales, definitions, time points) within the outcome domain? | NI | Protocol was not available. |
| 5.3 ... multiple eligible analyses of the data? | NI | Protocol was not available. |

## Yu Zhao, 2012

**Bias arising from the randomization process**

| Signaling question | Response | Supporting information |
| --- | --- | --- |
| 1.1 Was the allocation sequence random ? | NI | The author did not report random number sequence generation in detail. |
| 1.2 Was the allocation sequence concealed until participants were enrolled and assigned to interventions ? | NI | The author did not report allocation sequence concealment in detail. |
| 1.3 Did baseline differences between intervention groups suggest a problem with the randomization process? | N | The baseline characteristics was balanced. |

**bias due to deviations from the intended interventions (effect of assignment to intervention)**

| Signaling question | Response | Supporting information |
| --- | --- | --- |
| 2.1. Were participants aware of their assigned intervention during the trial? | Y | Blinding was not used. |
| 2.2. Were carers and people delivering the interventions aware of participants' assigned intervention during the trial? | Y | Blinding was not used. |
| 2.3. If Y/PY/NI to 2.1 or 2.2: Were there deviations from the intended intervention that arose because of the trial context? | NI | No enough information. |
| 2.4 If Y/PY to 2.3: Were these deviations likely to have affected the outcome? | NA | - |
| 2.5. If Y/PY/NI to 2.4: Were these deviations from intended intervention balanced between groups? | NA | - |
| 2.6 Was an appropriate analysis used to estimate the effect of assignment to intervention? | N | ITT analysis was not used. |
| 2.7 If N/PN/NI to 2.6: Was there potential for a substantial impact (on the result) of the failure to analyze participants in the group to which they were randomized? | NI | No enough information. |

**Bias due to missing outcome data**

| Signaling question | Response | Supporting information |
| --- | --- | --- |
| 3.1 Were data for this outcome available for all, or nearly all, participants randomized? | Y | Authors reported data for almost all subjects participating in randomization. |
| 3.2 If N/PN/NI to 3.1: Is there evidence that the result was not biased by missing outcome data? | NA |  |
| 3.3 If N/PN to 3.2: Could missingness in the outcome depend on its true value? | NA |  |
| 3.4 If Y/PY/NI to 3.3: Is it likely that missingness in the outcome depended on its true value? | NA | . |

**bias in measurement of the outcome**

| Signaling question | Response | Supporting information |
| --- | --- | --- |
| 4.1 Was the method of measuring the outcome inappropriate? | N | The method was appropriate. |
| 4.2 Could measurement or ascertainment of the outcome have differed between intervention groups? | N | There was no difference between groups. |
| 4.3 If N/PN/NI to 4.1 and 4.2: Were outcome assessors aware of the intervention received by study participants? | Y | Blinding was not used. |
| 4.4 If Y/PY/NI to 4.3: Could assessment of the outcome have been influenced by knowledge of intervention received? | N | The outcome involves no judgement. |
| 4.5 If Y/PY/NI to 4.4: Is it likely that assessment of the outcome was influenced by knowledge of intervention received? | NA |  |

**bias in selection of the reported result**

| Signaling question | Response | Supporting information |
| --- | --- | --- |
| 5.1 Were the data that produced this result analyzed in accordance with a pre-specified analysis plan that was finalized before unblinded outcome data were available for analysis? | NI | Protocol was not available. |
| s the numerical result being assessed likely to have been selected, on the basis of the results, from... |  |  |
| 5.2. ... multiple eligible outcome measurements (e.g. scales, definitions, time points) within the outcome domain? | NI | Protocol was not available. |
| 5.3 ... multiple eligible analyses of the data? | NI | Protocol was not available. |

## Bin Jiang, 2020

**Bias arising from the randomization process**

| Signaling question | Response | Supporting information |
| --- | --- | --- |
| 1.1 Was the allocation sequence random ? | NI | The author did not report random number sequence generation in detail. |
| 1.2 Was the allocation sequence concealed until participants were enrolled and assigned to interventions ? | NI | The author did not report allocation sequence concealment in detail. |
| 1.3 Did baseline differences between intervention groups suggest a problem with the randomization process? | NI | The baseline was not detailed enough. |

**bias due to deviations from the intended interventions (effect of assignment to intervention)**

| Signaling question | Response | Supporting information |
| --- | --- | --- |
| 2.1. Were participants aware of their assigned intervention during the trial? | Y | Blinding was not used. |
| 2.2. Were carers and people delivering the interventions aware of participants' assigned intervention during the trial? | Y | Blinding was not used. |
| 2.3. If Y/PY/NI to 2.1 or 2.2: Were there deviations from the intended intervention that arose because of the trial context? | NI | No enough information. |
| 2.4 If Y/PY to 2.3: Were these deviations likely to have affected the outcome? | NA | - |
| 2.5. If Y/PY/NI to 2.4: Were these deviations from intended intervention balanced between groups? | NA | - |
| 2.6 Was an appropriate analysis used to estimate the effect of assignment to intervention? | N | ITT analysis was not used. |
| 2.7 If N/PN/NI to 2.6: Was there potential for a substantial impact (on the result) of the failure to analyze participants in the group to which they were randomized? | NI | No enough information. |

**Bias due to missing outcome data**

| Signaling question | Response | Supporting information |
| --- | --- | --- |
| 3.1 Were data for this outcome available for all, or nearly all, participants randomized? | Y | Authors reported data for almost all subjects participating in randomization. |
| 3.2 If N/PN/NI to 3.1: Is there evidence that the result was not biased by missing outcome data? | NA |  |
| 3.3 If N/PN to 3.2: Could missingness in the outcome depend on its true value? | NA |  |
| 3.4 If Y/PY/NI to 3.3: Is it likely that missingness in the outcome depended on its true value? | NA | . |

**bias in measurement of the outcome**

| Signaling question | Response | Supporting information |
| --- | --- | --- |
| 4.1 Was the method of measuring the outcome inappropriate? | N | The method was appropriate. |
| 4.2 Could measurement or ascertainment of the outcome have differed between intervention groups? | N | There was no difference between groups. |
| 4.3 If N/PN/NI to 4.1 and 4.2: Were outcome assessors aware of the intervention received by study participants? | Y | Blinding was not used. |
| 4.4 If Y/PY/NI to 4.3: Could assessment of the outcome have been influenced by knowledge of intervention received? | N | The outcome involves no judgement. |
| 4.5 If Y/PY/NI to 4.4: Is it likely that assessment of the outcome was influenced by knowledge of intervention received? | NA |  |

**bias in selection of the reported result**

| Signaling question | Response | Supporting information |
| --- | --- | --- |
| 5.1 Were the data that produced this result analyzed in accordance with a pre-specified analysis plan that was finalized before unblinded outcome data were available for analysis? | NI | Protocol was not available. |
| s the numerical result being assessed likely to have been selected, on the basis of the results, from... |  |  |
| 5.2. ... multiple eligible outcome measurements (e.g. scales, definitions, time points) within the outcome domain? | NI | Protocol was not available. |
| 5.3 ... multiple eligible analyses of the data? | NI | Protocol was not available. |

## Shufang Geng, 2020

**Bias arising from the randomization process**

| Signaling question | Response | Supporting information |
| --- | --- | --- |
| 1.1 Was the allocation sequence random ? | PN | The author did not use random sequence generation. |
| 1.2 Was the allocation sequence concealed until participants were enrolled and assigned to interventions ? | PN | The author did not use allocation sequence concealment. |
| 1.3 Did baseline differences between intervention groups suggest a problem with the randomization process? | NI | The baseline was not detailed enough. |

**bias due to deviations from the intended interventions (effect of assignment to intervention)**

| Signaling question | Response | Supporting information |
| --- | --- | --- |
| 2.1. Were participants aware of their assigned intervention during the trial? | Y | Blinding was not used. |
| 2.2. Were carers and people delivering the interventions aware of participants' assigned intervention during the trial? | Y | Blinding was not used. |
| 2.3. If Y/PY/NI to 2.1 or 2.2: Were there deviations from the intended intervention that arose because of the trial context? | NI | No enough information. |
| 2.4 If Y/PY to 2.3: Were these deviations likely to have affected the outcome? | NA | - |
| 2.5. If Y/PY/NI to 2.4: Were these deviations from intended intervention balanced between groups? | NA | - |
| 2.6 Was an appropriate analysis used to estimate the effect of assignment to intervention? | N | ITT analysis was not used. |
| 2.7 If N/PN/NI to 2.6: Was there potential for a substantial impact (on the result) of the failure to analyze participants in the group to which they were randomized? | NI | No enough information. |

**Bias due to missing outcome data**

| Signaling question | Response | Supporting information |
| --- | --- | --- |
| 3.1 Were data for this outcome available for all, or nearly all, participants randomized? | Y | Authors reported data for almost all subjects participating in randomization. |
| 3.2 If N/PN/NI to 3.1: Is there evidence that the result was not biased by missing outcome data? | NA |  |
| 3.3 If N/PN to 3.2: Could missingness in the outcome depend on its true value? | NA |  |
| 3.4 If Y/PY/NI to 3.3: Is it likely that missingness in the outcome depended on its true value? | NA | . |

**bias in measurement of the outcome**

| Signaling question | Response | Supporting information |
| --- | --- | --- |
| 4.1 Was the method of measuring the outcome inappropriate? | N | The method was appropriate. |
| 4.2 Could measurement or ascertainment of the outcome have differed between intervention groups? | N | There was no difference between groups. |
| 4.3 If N/PN/NI to 4.1 and 4.2: Were outcome assessors aware of the intervention received by study participants? | Y | Blinding was not used. |
| 4.4 If Y/PY/NI to 4.3: Could assessment of the outcome have been influenced by knowledge of intervention received? | N | The outcome involves no judgement. |
| 4.5 If Y/PY/NI to 4.4: Is it likely that assessment of the outcome was influenced by knowledge of intervention received? | NA |  |

**bias in selection of the reported result**

| Signaling question | Response | Supporting information |
| --- | --- | --- |
| 5.1 Were the data that produced this result analyzed in accordance with a pre-specified analysis plan that was finalized before unblinded outcome data were available for analysis? | NI | Protocol was not available. |
| s the numerical result being assessed likely to have been selected, on the basis of the results, from... |  |  |
| 5.2. ... multiple eligible outcome measurements (e.g. scales, definitions, time points) within the outcome domain? | NI | Protocol was not available. |
| 5.3 ... multiple eligible analyses of the data? | NI | Protocol was not available. |

## Jingzu Zhang, 2019

**Bias arising from the randomization process**

| Signaling question | Response | Supporting information |
| --- | --- | --- |
| 1.1 Was the allocation sequence random ? | Y | The author uses random numbers tables. |
| 1.2 Was the allocation sequence concealed until participants were enrolled and assigned to interventions ? | NI | The author did not report allocation sequence concealment in detail. |
| 1.3 Did baseline differences between intervention groups suggest a problem with the randomization process? | PN | The baseline was probably balanced. |

**bias due to deviations from the intended interventions (effect of assignment to intervention)**

| Signaling question | Response | Supporting information |
| --- | --- | --- |
| 2.1. Were participants aware of their assigned intervention during the trial? | Y | Blinding was not used. |
| 2.2. Were carers and people delivering the interventions aware of participants' assigned intervention during the trial? | Y | Blinding was not used. |
| 2.3. If Y/PY/NI to 2.1 or 2.2: Were there deviations from the intended intervention that arose because of the trial context? | NI | No enough information. |
| 2.4 If Y/PY to 2.3: Were these deviations likely to have affected the outcome? | NA | - |
| 2.5. If Y/PY/NI to 2.4: Were these deviations from intended intervention balanced between groups? | NA | - |
| 2.6 Was an appropriate analysis used to estimate the effect of assignment to intervention? | N | ITT analysis was not used. |
| 2.7 If N/PN/NI to 2.6: Was there potential for a substantial impact (on the result) of the failure to analyze participants in the group to which they were randomized? | NI | No enough information. |

**Bias due to missing outcome data**

| Signaling question | Response | Supporting information |
| --- | --- | --- |
| 3.1 Were data for this outcome available for all, or nearly all, participants randomized? | Y | Authors reported data for almost all subjects participating in randomization. |
| 3.2 If N/PN/NI to 3.1: Is there evidence that the result was not biased by missing outcome data? | NA |  |
| 3.3 If N/PN to 3.2: Could missingness in the outcome depend on its true value? | NA |  |
| 3.4 If Y/PY/NI to 3.3: Is it likely that missingness in the outcome depended on its true value? | NA | . |

**bias in measurement of the outcome**

| Signaling question | Response | Supporting information |
| --- | --- | --- |
| 4.1 Was the method of measuring the outcome inappropriate? | N | The method was appropriate. |
| 4.2 Could measurement or ascertainment of the outcome have differed between intervention groups? | N | There was no difference between groups. |
| 4.3 If N/PN/NI to 4.1 and 4.2: Were outcome assessors aware of the intervention received by study participants? | Y | Blinding was not used. |
| 4.4 If Y/PY/NI to 4.3: Could assessment of the outcome have been influenced by knowledge of intervention received? | N | The outcome involves no judgement. |
| 4.5 If Y/PY/NI to 4.4: Is it likely that assessment of the outcome was influenced by knowledge of intervention received? | NA |  |

**bias in selection of the reported result**

| Signaling question | Response | Supporting information |
| --- | --- | --- |
| 5.1 Were the data that produced this result analyzed in accordance with a pre-specified analysis plan that was finalized before unblinded outcome data were available for analysis? | NI | Protocol was not available. |
| s the numerical result being assessed likely to have been selected, on the basis of the results, from... |  |  |
| 5.2. ... multiple eligible outcome measurements (e.g. scales, definitions, time points) within the outcome domain? | NI | Protocol was not available. |
| 5.3 ... multiple eligible analyses of the data? | NI | Protocol was not available. |

## Hongyun Sun, 2018

**Bias arising from the randomization process**

| Signaling question | Response | Supporting information |
| --- | --- | --- |
| 1.1 Was the allocation sequence random ? | N | The author did not use random methods. |
| 1.2 Was the allocation sequence concealed until participants were enrolled and assigned to interventions ? | N | The author did use allocation sequence concealment. |
| 1.3 Did baseline differences between intervention groups suggest a problem with the randomization process? | NI | The baseline was not detailed enough. |

**bias due to deviations from the intended interventions (effect of assignment to intervention)**

| Signaling question | Response | Supporting information |
| --- | --- | --- |
| 2.1. Were participants aware of their assigned intervention during the trial? | Y | Blinding was not used. |
| 2.2. Were carers and people delivering the interventions aware of participants' assigned intervention during the trial? | Y | Blinding was not used. |
| 2.3. If Y/PY/NI to 2.1 or 2.2: Were there deviations from the intended intervention that arose because of the trial context? | NI | No enough information. |
| 2.4 If Y/PY to 2.3: Were these deviations likely to have affected the outcome? | NA | - |
| 2.5. If Y/PY/NI to 2.4: Were these deviations from intended intervention balanced between groups? | NA | - |
| 2.6 Was an appropriate analysis used to estimate the effect of assignment to intervention? | N | ITT analysis was not used. |
| 2.7 If N/PN/NI to 2.6: Was there potential for a substantial impact (on the result) of the failure to analyze participants in the group to which they were randomized? | NI | No enough information. |

**Bias due to missing outcome data**

| Signaling question | Response | Supporting information |
| --- | --- | --- |
| 3.1 Were data for this outcome available for all, or nearly all, participants randomized? | Y | Authors reported data for almost all subjects participating in randomization. |
| 3.2 If N/PN/NI to 3.1: Is there evidence that the result was not biased by missing outcome data? | NA |  |
| 3.3 If N/PN to 3.2: Could missingness in the outcome depend on its true value? | NA |  |
| 3.4 If Y/PY/NI to 3.3: Is it likely that missingness in the outcome depended on its true value? | NA | . |

**bias in measurement of the outcome**

| Signaling question | Response | Supporting information |
| --- | --- | --- |
| 4.1 Was the method of measuring the outcome inappropriate? | N | The method was appropriate. |
| 4.2 Could measurement or ascertainment of the outcome have differed between intervention groups? | N | There was no difference between groups. |
| 4.3 If N/PN/NI to 4.1 and 4.2: Were outcome assessors aware of the intervention received by study participants? | Y | Blinding was not used. |
| 4.4 If Y/PY/NI to 4.3: Could assessment of the outcome have been influenced by knowledge of intervention received? | N | The outcome involves no judgement. |
| 4.5 If Y/PY/NI to 4.4: Is it likely that assessment of the outcome was influenced by knowledge of intervention received? | NA |  |

**bias in selection of the reported result**

| Signaling question | Response | Supporting information |
| --- | --- | --- |
| 5.1 Were the data that produced this result analyzed in accordance with a pre-specified analysis plan that was finalized before unblinded outcome data were available for analysis? | NI | Protocol was not available. |
| s the numerical result being assessed likely to have been selected, on the basis of the results, from... |  |  |
| 5.2. ... multiple eligible outcome measurements (e.g. scales, definitions, time points) within the outcome domain? | NI | Protocol was not available. |
| 5.3 ... multiple eligible analyses of the data? | NI | Protocol was not available. |

## Cuirong Hou, 2017

**Bias arising from the randomization process**

| Signaling question | Response | Supporting information |
| --- | --- | --- |
| 1.1 Was the allocation sequence random ? | NI | The author did not report in detail. |
| 1.2 Was the allocation sequence concealed until participants were enrolled and assigned to interventions ? | NI | The author did not report in detail. |
| 1.3 Did baseline differences between intervention groups suggest a problem with the randomization process? | NI | The baseline was not detailed enough. |

**bias due to deviations from the intended interventions (effect of assignment to intervention)**

| Signaling question | Response | Supporting information |
| --- | --- | --- |
| 2.1. Were participants aware of their assigned intervention during the trial? | Y | Blinding was not used. |
| 2.2. Were carers and people delivering the interventions aware of participants' assigned intervention during the trial? | Y | Blinding was not used. |
| 2.3. If Y/PY/NI to 2.1 or 2.2: Were there deviations from the intended intervention that arose because of the trial context? | NI | No enough information. |
| 2.4 If Y/PY to 2.3: Were these deviations likely to have affected the outcome? | NA | - |
| 2.5. If Y/PY/NI to 2.4: Were these deviations from intended intervention balanced between groups? | NA | - |
| 2.6 Was an appropriate analysis used to estimate the effect of assignment to intervention? | N | ITT analysis was not used. |
| 2.7 If N/PN/NI to 2.6: Was there potential for a substantial impact (on the result) of the failure to analyze participants in the group to which they were randomized? | NI | No enough information. |

**Bias due to missing outcome data**

| Signaling question | Response | Supporting information |
| --- | --- | --- |
| 3.1 Were data for this outcome available for all, or nearly all, participants randomized? | Y | Authors reported data for almost all subjects participating in randomization. |
| 3.2 If N/PN/NI to 3.1: Is there evidence that the result was not biased by missing outcome data? | NA |  |
| 3.3 If N/PN to 3.2: Could missingness in the outcome depend on its true value? | NA |  |
| 3.4 If Y/PY/NI to 3.3: Is it likely that missingness in the outcome depended on its true value? | NA | . |

**bias in measurement of the outcome**

| Signaling question | Response | Supporting information |
| --- | --- | --- |
| 4.1 Was the method of measuring the outcome inappropriate? | N | The method was appropriate. |
| 4.2 Could measurement or ascertainment of the outcome have differed between intervention groups? | N | There was no difference between groups. |
| 4.3 If N/PN/NI to 4.1 and 4.2: Were outcome assessors aware of the intervention received by study participants? | Y | Blinding was not used. |
| 4.4 If Y/PY/NI to 4.3: Could assessment of the outcome have been influenced by knowledge of intervention received? | N | The outcome involves no judgement. |
| 4.5 If Y/PY/NI to 4.4: Is it likely that assessment of the outcome was influenced by knowledge of intervention received? | NA |  |

**bias in selection of the reported result**

| Signaling question | Response | Supporting information |
| --- | --- | --- |
| 5.1 Were the data that produced this result analyzed in accordance with a pre-specified analysis plan that was finalized before unblinded outcome data were available for analysis? | NI | Protocol was not available. |
| s the numerical result being assessed likely to have been selected, on the basis of the results, from... |  |  |
| 5.2. ... multiple eligible outcome measurements (e.g. scales, definitions, time points) within the outcome domain? | NI | Protocol was not available. |
| 5.3 ... multiple eligible analyses of the data? | NI | Protocol was not available. |

## Lina Chen, 2012

**Bias arising from the randomization process**

| Signaling question | Response | Supporting information |
| --- | --- | --- |
| 1.1 Was the allocation sequence random ? | NI | The author did not report in detail. |
| 1.2 Was the allocation sequence concealed until participants were enrolled and assigned to interventions ? | NI | The author did not report in detail. |
| 1.3 Did baseline differences between intervention groups suggest a problem with the randomization process? | NI | The baseline was not detailed enough. |

**bias due to deviations from the intended interventions (effect of assignment to intervention)**

| Signaling question | Response | Supporting information |
| --- | --- | --- |
| 2.1. Were participants aware of their assigned intervention during the trial? | Y | Blinding was not used. |
| 2.2. Were carers and people delivering the interventions aware of participants' assigned intervention during the trial? | Y | Blinding was not used. |
| 2.3. If Y/PY/NI to 2.1 or 2.2: Were there deviations from the intended intervention that arose because of the trial context? | NI | No enough information. |
| 2.4 If Y/PY to 2.3: Were these deviations likely to have affected the outcome? | NA | - |
| 2.5. If Y/PY/NI to 2.4: Were these deviations from intended intervention balanced between groups? | NA | - |
| 2.6 Was an appropriate analysis used to estimate the effect of assignment to intervention? | N | ITT analysis was not used. |
| 2.7 If N/PN/NI to 2.6: Was there potential for a substantial impact (on the result) of the failure to analyze participants in the group to which they were randomized? | NI | No enough information. |

**Bias due to missing outcome data**

| Signaling question | Response | Supporting information |
| --- | --- | --- |
| 3.1 Were data for this outcome available for all, or nearly all, participants randomized? | Y | Authors reported data for almost all subjects participating in randomization. |
| 3.2 If N/PN/NI to 3.1: Is there evidence that the result was not biased by missing outcome data? | NA |  |
| 3.3 If N/PN to 3.2: Could missingness in the outcome depend on its true value? | NA |  |
| 3.4 If Y/PY/NI to 3.3: Is it likely that missingness in the outcome depended on its true value? | NA | . |

**bias in measurement of the outcome**

| Signaling question | Response | Supporting information |
| --- | --- | --- |
| 4.1 Was the method of measuring the outcome inappropriate? | N | The method was appropriate. |
| 4.2 Could measurement or ascertainment of the outcome have differed between intervention groups? | N | There was no difference between groups. |
| 4.3 If N/PN/NI to 4.1 and 4.2: Were outcome assessors aware of the intervention received by study participants? | Y | Blinding was not used. |
| 4.4 If Y/PY/NI to 4.3: Could assessment of the outcome have been influenced by knowledge of intervention received? | N | The outcome involves no judgement. |
| 4.5 If Y/PY/NI to 4.4: Is it likely that assessment of the outcome was influenced by knowledge of intervention received? | NA |  |

**bias in selection of the reported result**

| Signaling question | Response | Supporting information |
| --- | --- | --- |
| 5.1 Were the data that produced this result analyzed in accordance with a pre-specified analysis plan that was finalized before unblinded outcome data were available for analysis? | NI | Protocol was not available. |
| s the numerical result being assessed likely to have been selected, on the basis of the results, from... |  |  |
| 5.2. ... multiple eligible outcome measurements (e.g. scales, definitions, time points) within the outcome domain? | NI | Protocol was not available. |
| 5.3 ... multiple eligible analyses of the data? | NI | Protocol was not available. |

## Xiaoming Yang, 2011

**Bias arising from the randomization process**

| Signaling question | Response | Supporting information |
| --- | --- | --- |
| 1.1 Was the allocation sequence random ? | N | The author did not use random method. |
| 1.2 Was the allocation sequence concealed until participants were enrolled and assigned to interventions ? | N | The author did not use allocation sequence concealment. |
| 1.3 Did baseline differences between intervention groups suggest a problem with the randomization process? | NI | The baseline was not detailed enough. |

**bias due to deviations from the intended interventions (effect of assignment to intervention)**

| Signaling question | Response | Supporting information |
| --- | --- | --- |
| 2.1. Were participants aware of their assigned intervention during the trial? | Y | Blinding was not used. |
| 2.2. Were carers and people delivering the interventions aware of participants' assigned intervention during the trial? | Y | Blinding was not used. |
| 2.3. If Y/PY/NI to 2.1 or 2.2: Were there deviations from the intended intervention that arose because of the trial context? | NI | No enough information. |
| 2.4 If Y/PY to 2.3: Were these deviations likely to have affected the outcome? | NA | - |
| 2.5. If Y/PY/NI to 2.4: Were these deviations from intended intervention balanced between groups? | NA | - |
| 2.6 Was an appropriate analysis used to estimate the effect of assignment to intervention? | N | ITT analysis was not used. |
| 2.7 If N/PN/NI to 2.6: Was there potential for a substantial impact (on the result) of the failure to analyze participants in the group to which they were randomized? | NI | No enough information. |

**Bias due to missing outcome data**

| Signaling question | Response | Supporting information |
| --- | --- | --- |
| 3.1 Were data for this outcome available for all, or nearly all, participants randomized? | Y | Authors reported data for almost all subjects participating in randomization. |
| 3.2 If N/PN/NI to 3.1: Is there evidence that the result was not biased by missing outcome data? | NA |  |
| 3.3 If N/PN to 3.2: Could missingness in the outcome depend on its true value? | NA |  |
| 3.4 If Y/PY/NI to 3.3: Is it likely that missingness in the outcome depended on its true value? | NA | . |

**bias in measurement of the outcome**

| Signaling question | Response | Supporting information |
| --- | --- | --- |
| 4.1 Was the method of measuring the outcome inappropriate? | N | The method was appropriate. |
| 4.2 Could measurement or ascertainment of the outcome have differed between intervention groups? | N | There was no difference between groups. |
| 4.3 If N/PN/NI to 4.1 and 4.2: Were outcome assessors aware of the intervention received by study participants? | Y | Blinding was not used. |
| 4.4 If Y/PY/NI to 4.3: Could assessment of the outcome have been influenced by knowledge of intervention received? | N | The outcome involves no judgement. |
| 4.5 If Y/PY/NI to 4.4: Is it likely that assessment of the outcome was influenced by knowledge of intervention received? | NA |  |

**bias in selection of the reported result**

| Signaling question | Response | Supporting information |
| --- | --- | --- |
| 5.1 Were the data that produced this result analyzed in accordance with a pre-specified analysis plan that was finalized before unblinded outcome data were available for analysis? | NI | Protocol was not available. |
| s the numerical result being assessed likely to have been selected, on the basis of the results, from... |  |  |
| 5.2. ... multiple eligible outcome measurements (e.g. scales, definitions, time points) within the outcome domain? | NI | Protocol was not available. |
| 5.3 ... multiple eligible analyses of the data? | NI | Protocol was not available. |

## Zhaoyi Huang, 2010

**Bias arising from the randomization process**

| Signaling question | Response | Supporting information |
| --- | --- | --- |
| 1.1 Was the allocation sequence random ? | NI | The author did not report random method in detail. |
| 1.2 Was the allocation sequence concealed until participants were enrolled and assigned to interventions ? | NI | The author did not report allocation sequence concealment in detail. |
| 1.3 Did baseline differences between intervention groups suggest a problem with the randomization process? | NI | The baseline was not detailed enough. |

**bias due to deviations from the intended interventions (effect of assignment to intervention)**

| Signaling question | Response | Supporting information |
| --- | --- | --- |
| 2.1. Were participants aware of their assigned intervention during the trial? | Y | Blinding was not used. |
| 2.2. Were carers and people delivering the interventions aware of participants' assigned intervention during the trial? | Y | Blinding was not used. |
| 2.3. If Y/PY/NI to 2.1 or 2.2: Were there deviations from the intended intervention that arose because of the trial context? | NI | No enough information. |
| 2.4 If Y/PY to 2.3: Were these deviations likely to have affected the outcome? | NA | - |
| 2.5. If Y/PY/NI to 2.4: Were these deviations from intended intervention balanced between groups? | NA | - |
| 2.6 Was an appropriate analysis used to estimate the effect of assignment to intervention? | N | ITT analysis was not used. |
| 2.7 If N/PN/NI to 2.6: Was there potential for a substantial impact (on the result) of the failure to analyze participants in the group to which they were randomized? | NI | No enough information. |

**Bias due to missing outcome data**

| Signaling question | Response | Supporting information |
| --- | --- | --- |
| 3.1 Were data for this outcome available for all, or nearly all, participants randomized? | Y | Authors reported data for almost all subjects participating in randomization. |
| 3.2 If N/PN/NI to 3.1: Is there evidence that the result was not biased by missing outcome data? | NA |  |
| 3.3 If N/PN to 3.2: Could missingness in the outcome depend on its true value? | NA |  |
| 3.4 If Y/PY/NI to 3.3: Is it likely that missingness in the outcome depended on its true value? | NA | . |

**bias in measurement of the outcome**

| Signaling question | Response | Supporting information |
| --- | --- | --- |
| 4.1 Was the method of measuring the outcome inappropriate? | N | The method was appropriate. |
| 4.2 Could measurement or ascertainment of the outcome have differed between intervention groups? | N | There was no difference between groups. |
| 4.3 If N/PN/NI to 4.1 and 4.2: Were outcome assessors aware of the intervention received by study participants? | Y | Blinding was not used. |
| 4.4 If Y/PY/NI to 4.3: Could assessment of the outcome have been influenced by knowledge of intervention received? | N | The outcome involves no judgement. |
| 4.5 If Y/PY/NI to 4.4: Is it likely that assessment of the outcome was influenced by knowledge of intervention received? | NA |  |

**bias in selection of the reported result**

| Signaling question | Response | Supporting information |
| --- | --- | --- |
| 5.1 Were the data that produced this result analyzed in accordance with a pre-specified analysis plan that was finalized before unblinded outcome data were available for analysis? | NI | Protocol was not available. |
| s the numerical result being assessed likely to have been selected, on the basis of the results, from... |  |  |
| 5.2. ... multiple eligible outcome measurements (e.g. scales, definitions, time points) within the outcome domain? | NI | Protocol was not available. |
| 5.3 ... multiple eligible analyses of the data? | NI | Protocol was not available. |

## Xinyi Zhang, 2011

**Bias arising from the randomization process**

| Signaling question | Response | Supporting information |
| --- | --- | --- |
| 1.1 Was the allocation sequence random ? | NI | The author did not report random method in detail. |
| 1.2 Was the allocation sequence concealed until participants were enrolled and assigned to interventions ? | NI | The author did not report allocation sequence concealment in detail. |
| 1.3 Did baseline differences between intervention groups suggest a problem with the randomization process? | PN | The baseline was probably balanced. |

**bias due to deviations from the intended interventions (effect of assignment to intervention)**

| Signaling question | Response | Supporting information |
| --- | --- | --- |
| 2.1. Were participants aware of their assigned intervention during the trial? | Y | Blinding was not used. |
| 2.2. Were carers and people delivering the interventions aware of participants' assigned intervention during the trial? | Y | Blinding was not used. |
| 2.3. If Y/PY/NI to 2.1 or 2.2: Were there deviations from the intended intervention that arose because of the trial context? | NI | No enough information. |
| 2.4 If Y/PY to 2.3: Were these deviations likely to have affected the outcome? | NA | - |
| 2.5. If Y/PY/NI to 2.4: Were these deviations from intended intervention balanced between groups? | NA | - |
| 2.6 Was an appropriate analysis used to estimate the effect of assignment to intervention? | N | ITT analysis was not used. |
| 2.7 If N/PN/NI to 2.6: Was there potential for a substantial impact (on the result) of the failure to analyze participants in the group to which they were randomized? | NI | No enough information. |

**Bias due to missing outcome data**

| Signaling question | Response | Supporting information |
| --- | --- | --- |
| 3.1 Were data for this outcome available for all, or nearly all, participants randomized? | Y | Authors reported data for almost all subjects participating in randomization. |
| 3.2 If N/PN/NI to 3.1: Is there evidence that the result was not biased by missing outcome data? | NA |  |
| 3.3 If N/PN to 3.2: Could missingness in the outcome depend on its true value? | NA |  |
| 3.4 If Y/PY/NI to 3.3: Is it likely that missingness in the outcome depended on its true value? | NA | . |

**bias in measurement of the outcome**

| Signaling question | Response | Supporting information |
| --- | --- | --- |
| 4.1 Was the method of measuring the outcome inappropriate? | N | The method was appropriate. |
| 4.2 Could measurement or ascertainment of the outcome have differed between intervention groups? | N | There was no difference between groups. |
| 4.3 If N/PN/NI to 4.1 and 4.2: Were outcome assessors aware of the intervention received by study participants? | Y | Blinding was not used. |
| 4.4 If Y/PY/NI to 4.3: Could assessment of the outcome have been influenced by knowledge of intervention received? | N | The outcome involves no judgement. |
| 4.5 If Y/PY/NI to 4.4: Is it likely that assessment of the outcome was influenced by knowledge of intervention received? | NA |  |

**bias in selection of the reported result**

| Signaling question | Response | Supporting information |
| --- | --- | --- |
| 5.1 Were the data that produced this result analyzed in accordance with a pre-specified analysis plan that was finalized before unblinded outcome data were available for analysis? | NI | Protocol was not available. |
| s the numerical result being assessed likely to have been selected, on the basis of the results, from... |  |  |
| 5.2. ... multiple eligible outcome measurements (e.g. scales, definitions, time points) within the outcome domain? | NI | Protocol was not available. |
| 5.3 ... multiple eligible analyses of the data? | NI | Protocol was not available. |

## Yinzhong Li, 2012

**Bias arising from the randomization process**

| Signaling question | Response | Supporting information |
| --- | --- | --- |
| 1.1 Was the allocation sequence random ? | NI | The author did not report random method in detail. |
| 1.2 Was the allocation sequence concealed until participants were enrolled and assigned to interventions ? | NI | The author did not report allocation sequence concealment in detail. |
| 1.3 Did baseline differences between intervention groups suggest a problem with the randomization process? | NI | The baseline was not detailed. |

**bias due to deviations from the intended interventions (effect of assignment to intervention)**

| Signaling question | Response | Supporting information |
| --- | --- | --- |
| 2.1. Were participants aware of their assigned intervention during the trial? | Y | Blinding was not used. |
| 2.2. Were carers and people delivering the interventions aware of participants' assigned intervention during the trial? | Y | Blinding was not used. |
| 2.3. If Y/PY/NI to 2.1 or 2.2: Were there deviations from the intended intervention that arose because of the trial context? | NI | No enough information. |
| 2.4 If Y/PY to 2.3: Were these deviations likely to have affected the outcome? | NA | - |
| 2.5. If Y/PY/NI to 2.4: Were these deviations from intended intervention balanced between groups? | NA | - |
| 2.6 Was an appropriate analysis used to estimate the effect of assignment to intervention? | N | ITT analysis was not used. |
| 2.7 If N/PN/NI to 2.6: Was there potential for a substantial impact (on the result) of the failure to analyze participants in the group to which they were randomized? | NI | No enough information. |

**Bias due to missing outcome data**

| Signaling question | Response | Supporting information |
| --- | --- | --- |
| 3.1 Were data for this outcome available for all, or nearly all, participants randomized? | Y | Authors reported data for almost all subjects participating in randomization. |
| 3.2 If N/PN/NI to 3.1: Is there evidence that the result was not biased by missing outcome data? | NA |  |
| 3.3 If N/PN to 3.2: Could missingness in the outcome depend on its true value? | NA |  |
| 3.4 If Y/PY/NI to 3.3: Is it likely that missingness in the outcome depended on its true value? | NA | . |

**bias in measurement of the outcome**

| Signaling question | Response | Supporting information |
| --- | --- | --- |
| 4.1 Was the method of measuring the outcome inappropriate? | N | The method was appropriate. |
| 4.2 Could measurement or ascertainment of the outcome have differed between intervention groups? | N | There was no difference between groups. |
| 4.3 If N/PN/NI to 4.1 and 4.2: Were outcome assessors aware of the intervention received by study participants? | Y | Blinding was not used. |
| 4.4 If Y/PY/NI to 4.3: Could assessment of the outcome have been influenced by knowledge of intervention received? | N | The outcome involves no judgement. |
| 4.5 If Y/PY/NI to 4.4: Is it likely that assessment of the outcome was influenced by knowledge of intervention received? | NA |  |

**bias in selection of the reported result**

| Signaling question | Response | Supporting information |
| --- | --- | --- |
| 5.1 Were the data that produced this result analyzed in accordance with a pre-specified analysis plan that was finalized before unblinded outcome data were available for analysis? | NI | Protocol was not available. |
| s the numerical result being assessed likely to have been selected, on the basis of the results, from... |  |  |
| 5.2. ... multiple eligible outcome measurements (e.g. scales, definitions, time points) within the outcome domain? | NI | Protocol was not available. |
| 5.3 ... multiple eligible analyses of the data? | NI | Protocol was not available. |

## Yang Xiao, 2016

**Bias arising from the randomization process**

| Signaling question | Response | Supporting information |
| --- | --- | --- |
| 1.1 Was the allocation sequence random ? | Y | The author uses computer-generated random number. |
| 1.2 Was the allocation sequence concealed until participants were enrolled and assigned to interventions ? | NI | The author did not report allocation sequence concealment in detail. |
| 1.3 Did baseline differences between intervention groups suggest a problem with the randomization process? | PN | The baseline was not detailed. |

**bias due to deviations from the intended interventions (effect of assignment to intervention)**

| Signaling question | Response | Supporting information |
| --- | --- | --- |
| 2.1. Were participants aware of their assigned intervention during the trial? | Y | Blinding was not used. |
| 2.2. Were carers and people delivering the interventions aware of participants' assigned intervention during the trial? | Y | Blinding was not used. |
| 2.3. If Y/PY/NI to 2.1 or 2.2: Were there deviations from the intended intervention that arose because of the trial context? | NI | No enough information. |
| 2.4 If Y/PY to 2.3: Were these deviations likely to have affected the outcome? | NA | - |
| 2.5. If Y/PY/NI to 2.4: Were these deviations from intended intervention balanced between groups? | NA | - |
| 2.6 Was an appropriate analysis used to estimate the effect of assignment to intervention? | N | ITT analysis was not used. |
| 2.7 If N/PN/NI to 2.6: Was there potential for a substantial impact (on the result) of the failure to analyze participants in the group to which they were randomized? | NI | No enough information. |

**Bias due to missing outcome data**

| Signaling question | Response | Supporting information |
| --- | --- | --- |
| 3.1 Were data for this outcome available for all, or nearly all, participants randomized? | Y | Authors reported data for almost all subjects participating in randomization. |
| 3.2 If N/PN/NI to 3.1: Is there evidence that the result was not biased by missing outcome data? | NA |  |
| 3.3 If N/PN to 3.2: Could missingness in the outcome depend on its true value? | NA |  |
| 3.4 If Y/PY/NI to 3.3: Is it likely that missingness in the outcome depended on its true value? | NA | . |

**bias in measurement of the outcome**

| Signaling question | Response | Supporting information |
| --- | --- | --- |
| 4.1 Was the method of measuring the outcome inappropriate? | N | The method was appropriate. |
| 4.2 Could measurement or ascertainment of the outcome have differed between intervention groups? | N | There was no difference between groups. |
| 4.3 If N/PN/NI to 4.1 and 4.2: Were outcome assessors aware of the intervention received by study participants? | Y | Blinding was not used. |
| 4.4 If Y/PY/NI to 4.3: Could assessment of the outcome have been influenced by knowledge of intervention received? | N | The outcome involves no judgement. |
| 4.5 If Y/PY/NI to 4.4: Is it likely that assessment of the outcome was influenced by knowledge of intervention received? | NA |  |

**bias in selection of the reported result**

| Signaling question | Response | Supporting information |
| --- | --- | --- |
| 5.1 Were the data that produced this result analyzed in accordance with a pre-specified analysis plan that was finalized before unblinded outcome data were available for analysis? | NI | Protocol was not available. |
| s the numerical result being assessed likely to have been selected, on the basis of the results, from... |  |  |
| 5.2. ... multiple eligible outcome measurements (e.g. scales, definitions, time points) within the outcome domain? | NI | Protocol was not available. |
| 5.3 ... multiple eligible analyses of the data? | NI | Protocol was not available. |

## Fan Jia, 2016

**Bias arising from the randomization process**

| Signaling question | Response | Supporting information |
| --- | --- | --- |
| 1.1 Was the allocation sequence random ? | NI | The author uses computer-generated random number. |
| 1.2 Was the allocation sequence concealed until participants were enrolled and assigned to interventions ? | NI | The author did not report allocation sequence concealment in detail. |
| 1.3 Did baseline differences between intervention groups suggest a problem with the randomization process? | NI | The baseline was not detailed. |

**bias due to deviations from the intended interventions (effect of assignment to intervention)**

| Signaling question | Response | Supporting information |
| --- | --- | --- |
| 2.1. Were participants aware of their assigned intervention during the trial? | Y | Blinding was not used. |
| 2.2. Were carers and people delivering the interventions aware of participants' assigned intervention during the trial? | Y | Blinding was not used. |
| 2.3. If Y/PY/NI to 2.1 or 2.2: Were there deviations from the intended intervention that arose because of the trial context? | NI | No enough information. |
| 2.4 If Y/PY to 2.3: Were these deviations likely to have affected the outcome? | NA | - |
| 2.5. If Y/PY/NI to 2.4: Were these deviations from intended intervention balanced between groups? | NA | - |
| 2.6 Was an appropriate analysis used to estimate the effect of assignment to intervention? | N | ITT analysis was not used. |
| 2.7 If N/PN/NI to 2.6: Was there potential for a substantial impact (on the result) of the failure to analyze participants in the group to which they were randomized? | NI | No enough information. |

**Bias due to missing outcome data**

| Signaling question | Response | Supporting information |
| --- | --- | --- |
| 3.1 Were data for this outcome available for all, or nearly all, participants randomized? | Y | Authors reported data for almost all subjects participating in randomization. |
| 3.2 If N/PN/NI to 3.1: Is there evidence that the result was not biased by missing outcome data? | NA |  |
| 3.3 If N/PN to 3.2: Could missingness in the outcome depend on its true value? | NA |  |
| 3.4 If Y/PY/NI to 3.3: Is it likely that missingness in the outcome depended on its true value? | NA | . |

**bias in measurement of the outcome**

| Signaling question | Response | Supporting information |
| --- | --- | --- |
| 4.1 Was the method of measuring the outcome inappropriate? | NI | There is no enough information. |
| 4.2 Could measurement or ascertainment of the outcome have differed between intervention groups? | NI | There is no enough information. |
| 4.3 If N/PN/NI to 4.1 and 4.2: Were outcome assessors aware of the intervention received by study participants? | PY | Blinding was not used. |
| 4.4 If Y/PY/NI to 4.3: Could assessment of the outcome have been influenced by knowledge of intervention received? | PN | The outcome involves no judgement. |
| 4.5 If Y/PY/NI to 4.4: Is it likely that assessment of the outcome was influenced by knowledge of intervention received? | NA | - |

**bias in selection of the reported result**

| Signaling question | Response | Supporting information |
| --- | --- | --- |
| 5.1 Were the data that produced this result analyzed in accordance with a pre-specified analysis plan that was finalized before unblinded outcome data were available for analysis? | NI | Protocol was not available. |
| s the numerical result being assessed likely to have been selected, on the basis of the results, from... |  |  |
| 5.2. ... multiple eligible outcome measurements (e.g. scales, definitions, time points) within the outcome domain? | NI | Protocol was not available. |
| 5.3 ... multiple eligible analyses of the data? | NI | Protocol was not available. |

# Supporting information for data analysis

## 6.1 Forest plot of HbA1c by combination treatment compared with hypoglycemic agents alone





## 6.2 Sensitivity analysis of HbA1c by combination treatment compared with hypoglycemic agents alone





## 6.3 Subgroup analysis of HbA1c by combination treatment compared with hypoglycemic agents alone according to ages





## 6.4 Further comparisons of combination treatment compared with hypoglycemic agents on HbA1c based on specific medicines


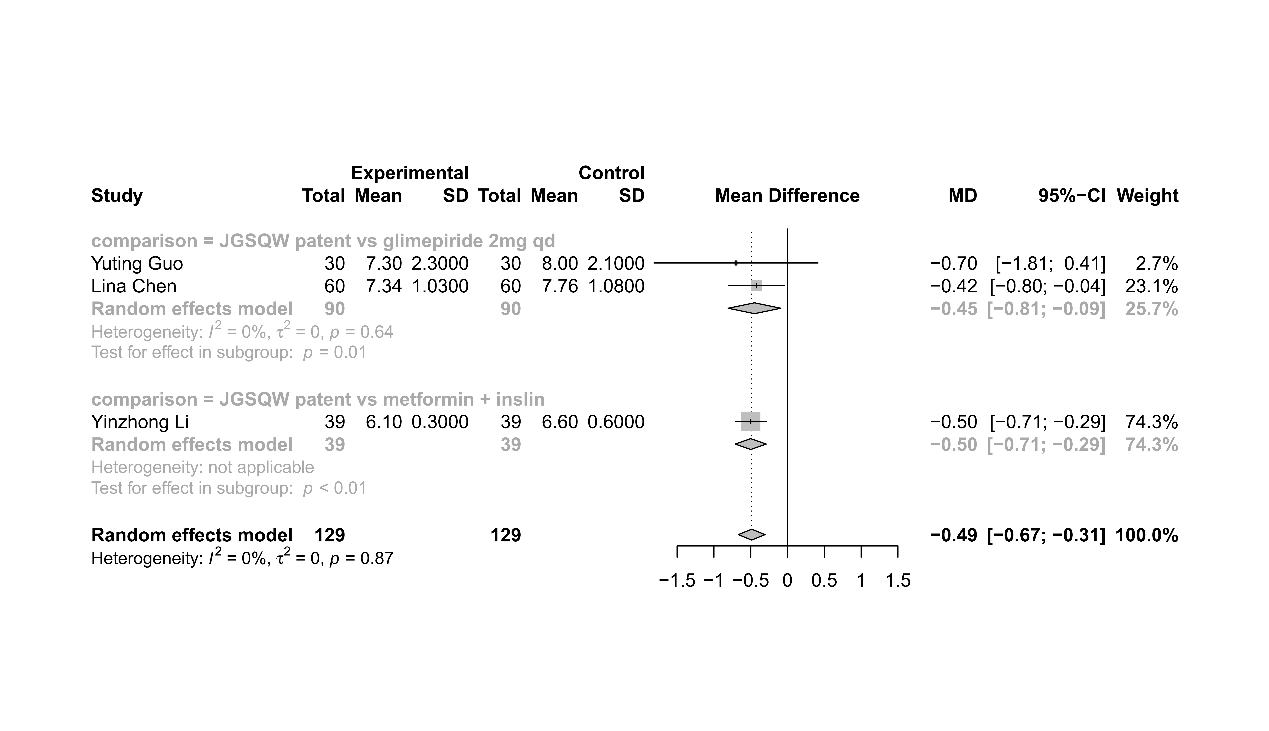


## 6.5 Forest plot of effect on HbA1c by JGSQW compared with hypoglycemic agents





## 6.6 Forest plot of FBG by combination treatment compared with hypoglycemic agents alone


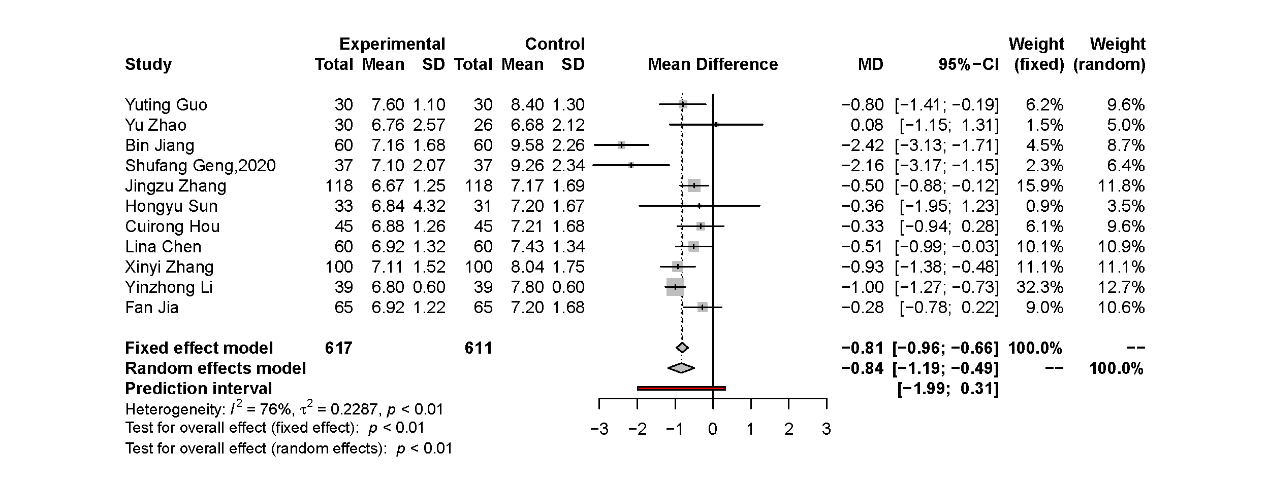


## 6.7 Sensitivity analysis of FBG by combination treatment compared with hypoglycemic agents alone





## 6.8 Subgroup analysis of FBG by combination treatment compared with hypoglycemic agents alone according to ages





## 6.9 Subgroup analysis of FBG by combination treatment compared with hypoglycemic agents alone according to comorbidity





## 6.10 Subgroup analysis of FBG by combination treatment compared with hypoglycemic agents alone according to forms of JGSQW


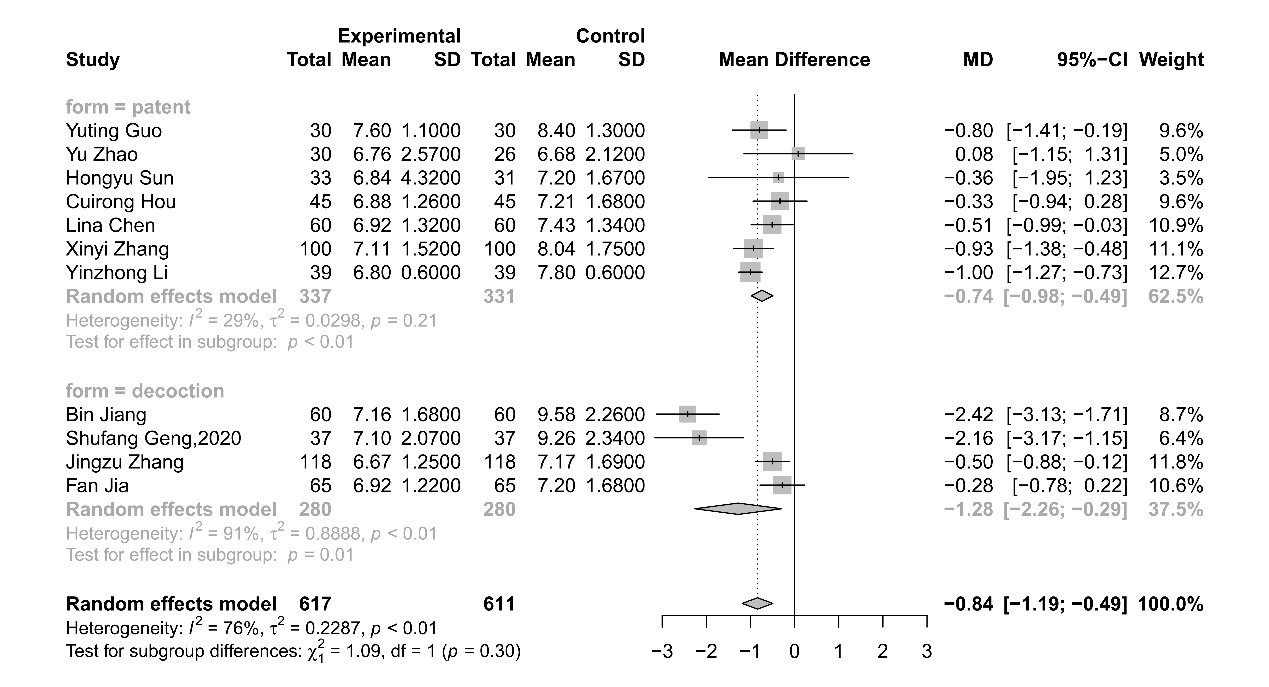


## 6.11 Subgroup analysis of FBG by combination treatment compared with hypoglycemic agents alone according to baseline level of FBG


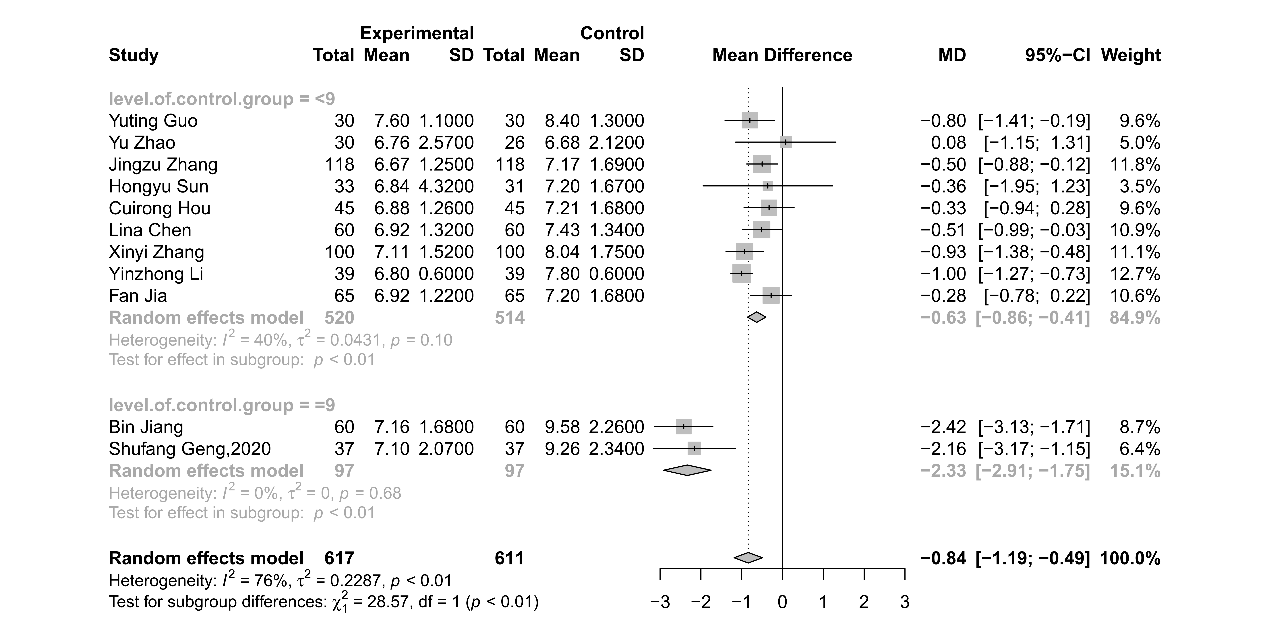


## 6.12 Further comparisons of combination treatment compared with hypoglycemic agents on FBG based on specific medicines


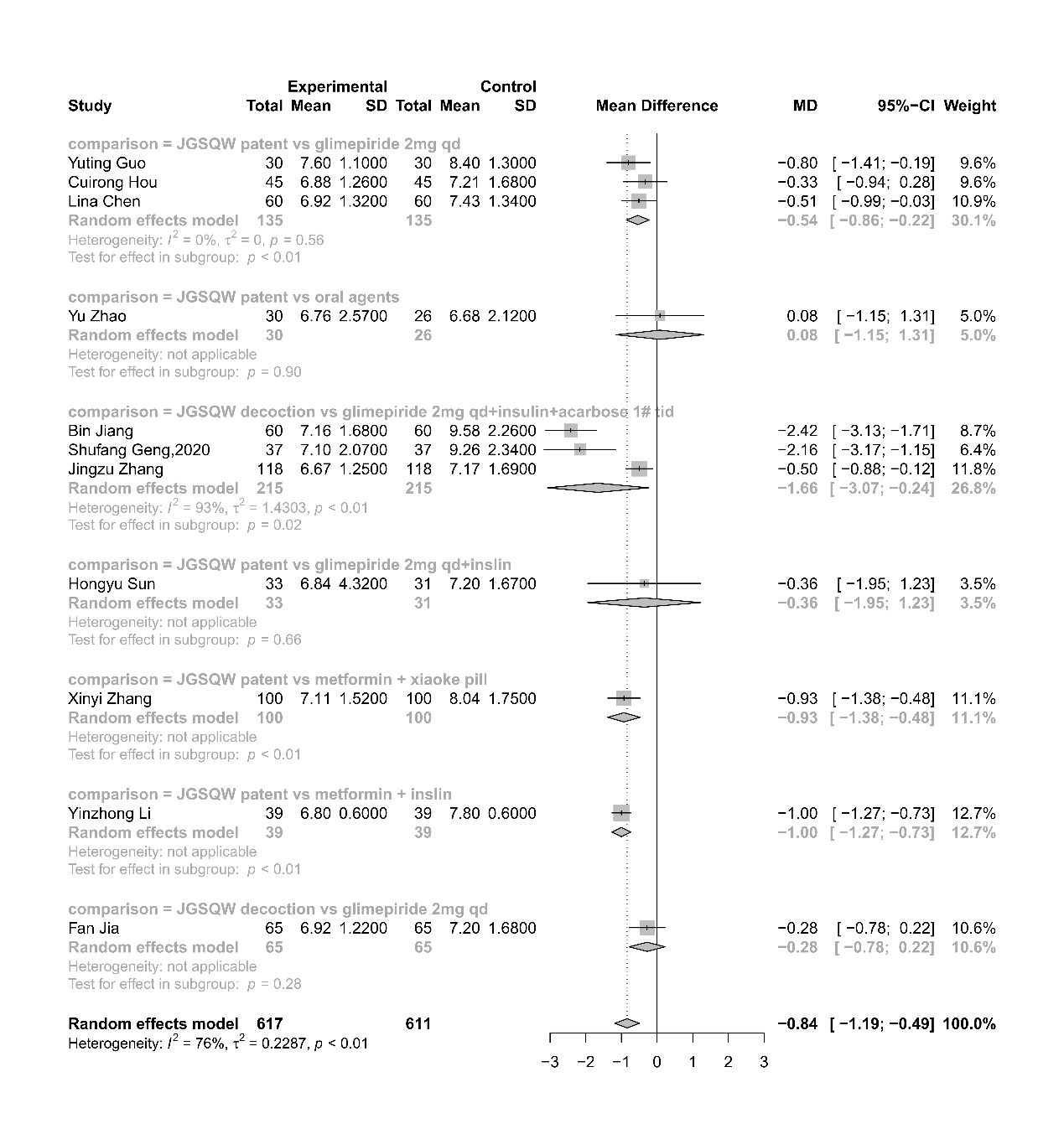


## 6.13 Effect on FBG by JGSQW compared with hypoglycemic agents





## 6.14 Forest plot of 2hBG by combination treatment compared with hypoglycemic agents alone


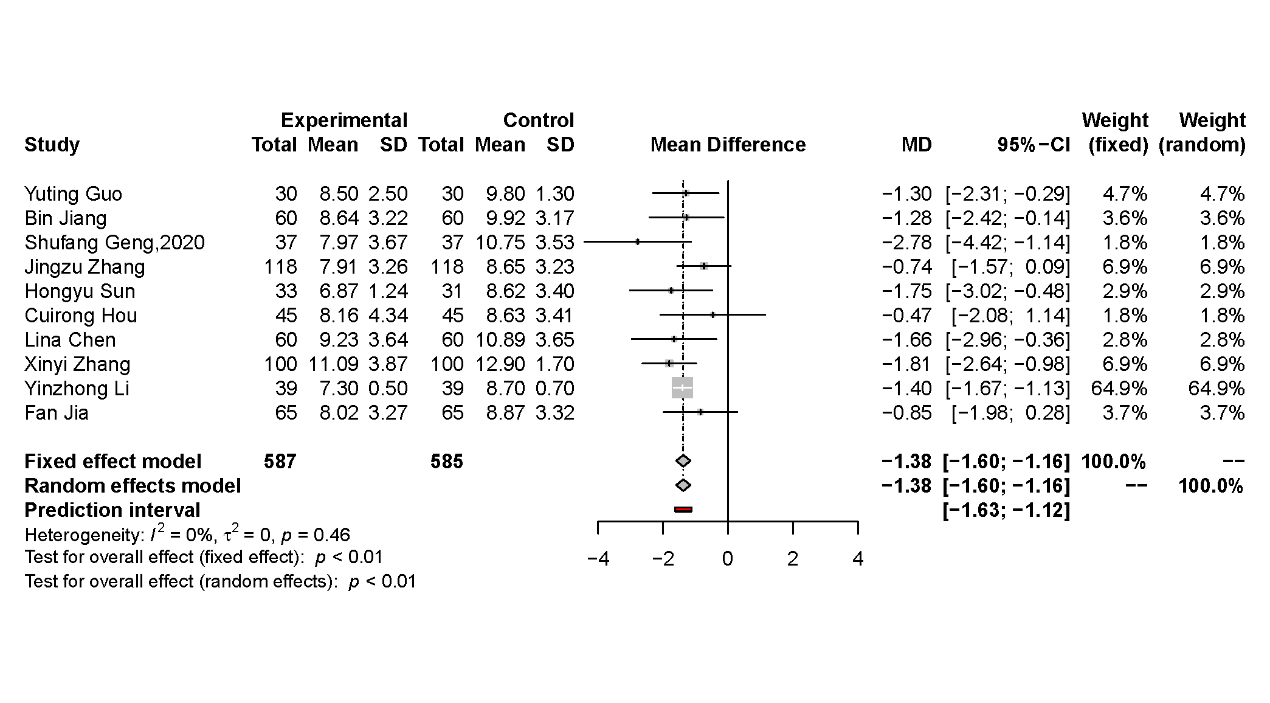


## 6.15 Sensitivity analysis of 2hBG by combination treatment compared with hypoglycemic agents alone





## 6.16 Subgroup analysis of 2hBG by combination treatment compared with hypoglycemic agents alone according to ages





## 6.17 Subgroup analysis of 2hBG by combination treatment compared with hypoglycemic agents alone according to comorbidity





## 6.18 Subgroup analysis of 2hBG by combination treatment compared with hypoglycemic agents alone according to forms of JGSQW


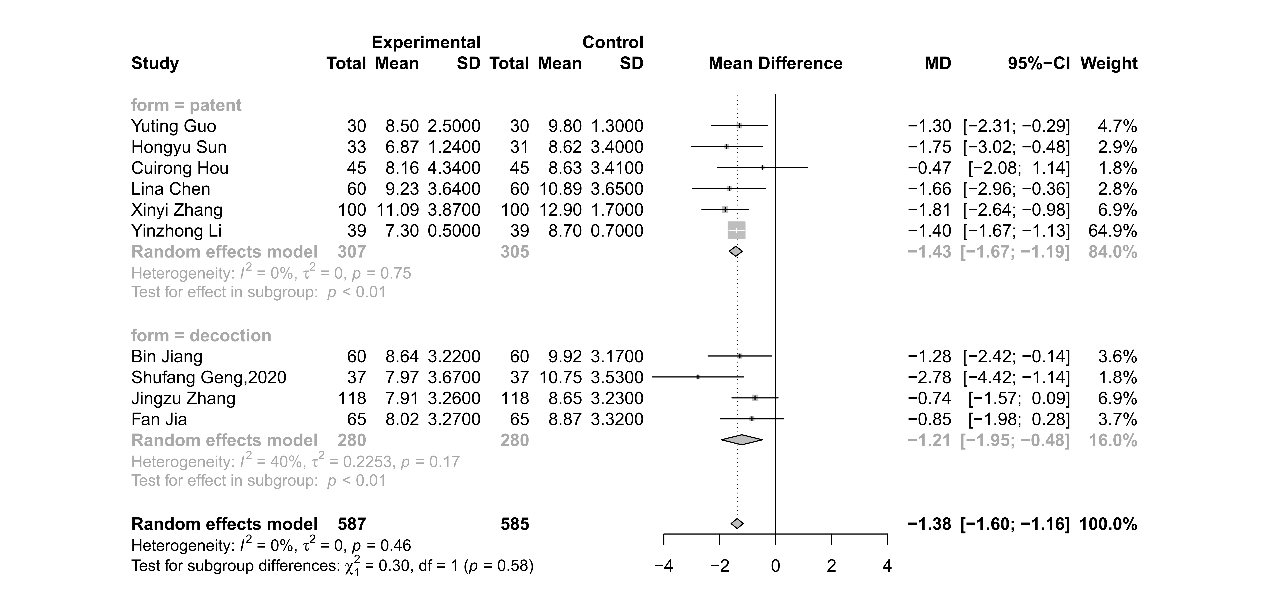


## 6.19 Further comparisons of combination treatment compared with hypoglycemic agents on 2hBG based on specific medicines


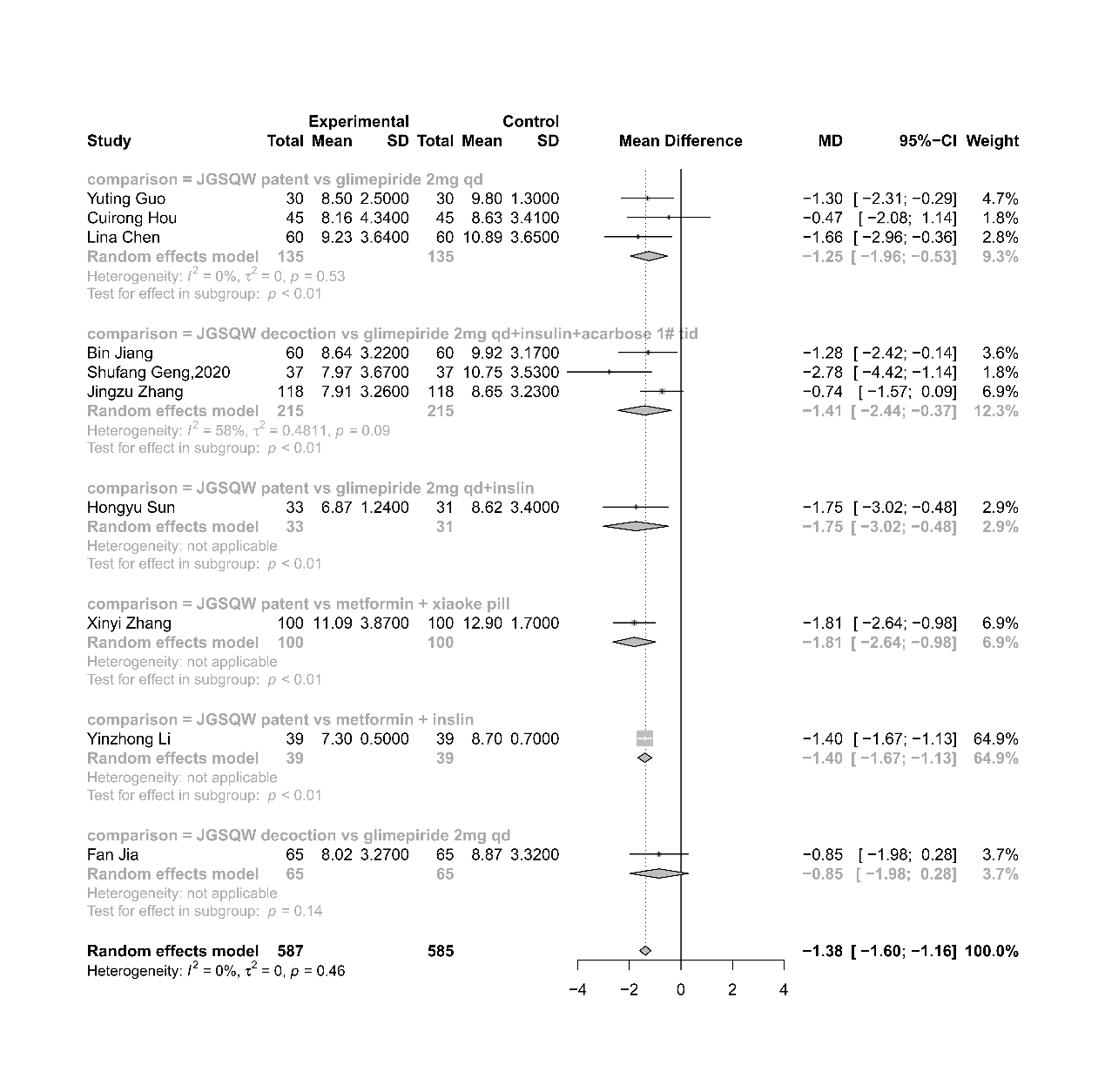


## 6.20 Forest plot on 2hBG by JGSQW compared with hypoglycemic agents





## 6.21 Forest plot of HDL-C by combination treatment compared with hypoglycemic agents alone





## 6.22 Forest plot of HDL-C by JGSQW compared with hypoglycemic agents





## 6.23 Forest plot of LDL-C by JGSQW compared with Xiaoke Pills





## 6.24 Forest plot on TC by combination treatment compared with hypoglycemic agents alone


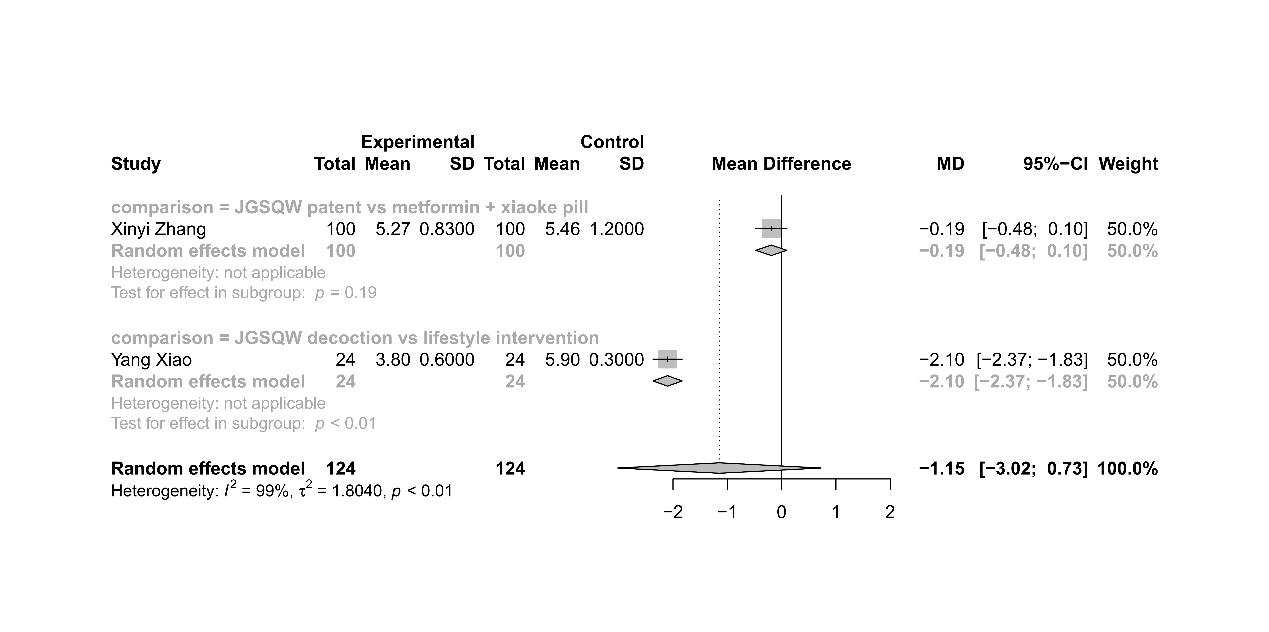


## 6.25 Forest plot of TC by JGSQW compared with hypoglycemic agents





## 6.26 Forest plot of TG by combination treatment compared with hypoglycemic agents alone


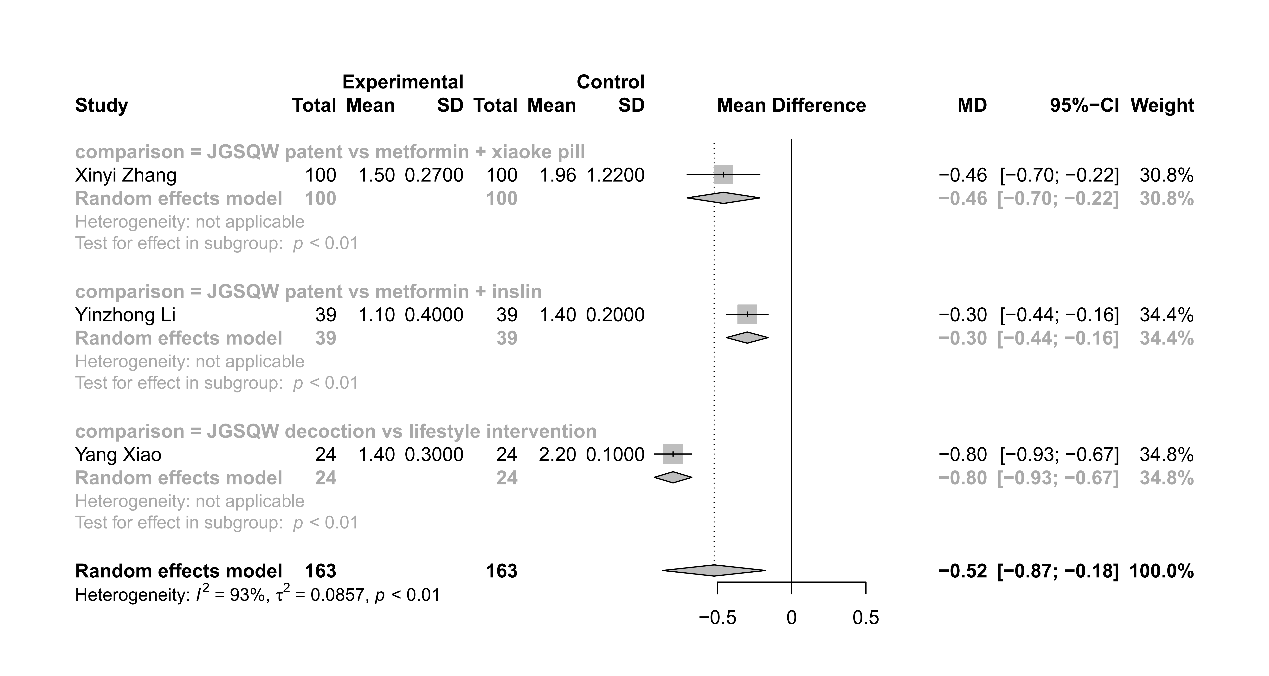


## 6.27 Forest plot of TG by JGSQW compared with hypoglycemic agents





## 6.28 Funnel plot of 2hBG





## 6.29 Funnel plot of FBG





# Phytochemical profile of preparation

## 7.1 Phytochemical profile of Rehmannia glutinosa (Gaertn.) DC. Orobanchaceae (*dì huáng*)

| [Mol ID](https://old.tcmsp-e.com/tcmspsearch.php?qr=Rehmanniae%20Radix%20Praeparata&qsr=herb_en_name&token=314294d6a2fa9a6cea11d6eaec01ab12) | **Molecule Name** | **MW** | **AlogP** | **Hdon** | **Hacc** | **OB (%)** | **Caco-2** | **BBB** | **DL** | **FASA-** | **HL** |
| --- | --- | --- | --- | --- | --- | --- | --- | --- | --- | --- | --- |
| MOL000359 | [sitosterol](https://old.tcmsp-e.com/molecule.php?qn=359) | 414.79 | 8.08 | 1 | 1 | 36.91 | 1.32 | 0.87 | 0.75 | 0.22 | 5.37 |
| MOL000449 | [Stigmasterol](https://old.tcmsp-e.com/molecule.php?qn=449) | 412.77 | 7.64 | 1 | 1 | 43.83 | 1.44 | 1 | 0.76 | 0.22 | 5.57 |

## 7.2 Phytochemical profile of Dioscorea oppositifolia L. Dioscoreaceae (*huái shān yào*)

| [Mol ID](https://old.tcmsp-e.com/tcmspsearch.php?qr=Rhizoma%20Dioscoreae&qsr=herb_en_name&token=314294d6a2fa9a6cea11d6eaec01ab12) | **Molecule Name** | **MW** | **AlogP** | **Hdon** | **Hacc** | **OB (%)** | **Caco-2** | **BBB** | **DL** | **FASA-** | **HL** |
| --- | --- | --- | --- | --- | --- | --- | --- | --- | --- | --- | --- |
| MOL001559 | [piperlonguminine](https://old.tcmsp-e.com/molecule.php?qn=1559) | 273.36 | 2.93 | 1 | 4 | 30.71 | 0.95 | 0.27 | 0.18 | 0.35 | 8.66 |
| MOL001736 | [(-)-taxifolin](https://old.tcmsp-e.com/molecule.php?qn=1736) | 304.27 | 1.49 | 5 | 7 | 60.51 | -0.24 | -1.02 | 0.27 | 0.41 | 14.37 |
| MOL000310 | [Denudatin B](https://old.tcmsp-e.com/molecule.php?qn=310) | 356.45 | 2.8 | 0 | 5 | 61.47 | 0.9 | 0.35 | 0.38 | 0.24 | 7.71 |
| MOL000322 | [Kadsurenone](https://old.tcmsp-e.com/molecule.php?qn=322) | 356.45 | 2.8 | 0 | 5 | 54.72 | 0.82 | 0.52 | 0.38 | 0.24 | 9.16 |
| MOL005429 | [hancinol](https://old.tcmsp-e.com/molecule.php?qn=5429) | 372.5 | 2.46 | 1 | 5 | 64.01 | 0.53 | 0.17 | 0.37 | 0.25 | 4.06 |
| MOL005430 | [hancinone C](https://old.tcmsp-e.com/molecule.php?qn=5430) | 400.51 | 3.34 | 0 | 6 | 59.05 | 0.74 | 0.06 | 0.39 | 0.19 | 4.14 |
| MOL005435 | [24-Methylcholest-5-enyl-3belta-O-glucopyranoside_qt](https://old.tcmsp-e.com/molecule.php?qn=5435) | 400.76 | 7.63 | 1 | 1 | 37.58 | 1.33 | 0.96 | 0.72 | 0.22 | 4.91 |
| MOL005438 | [campesterol](https://old.tcmsp-e.com/molecule.php?qn=5438) | 400.76 | 7.63 | 1 | 1 | 37.58 | 1.34 | 0.95 | 0.71 | 0.22 | 4.83 |
| MOL005440 | [Isofucosterol](https://old.tcmsp-e.com/molecule.php?qn=5440) | 412.77 | 7.83 | 1 | 1 | 43.78 | 1.36 | 0.97 | 0.76 | 0.24 | 5.18 |
| MOL000449 | [Stigmasterol](https://old.tcmsp-e.com/molecule.php?qn=449) | 412.77 | 7.64 | 1 | 1 | 43.83 | 1.44 | 1 | 0.76 | 0.22 | 5.57 |
| MOL005458 | [Dioscoreside C_qt](https://old.tcmsp-e.com/molecule.php?qn=5458) | 444.72 | 3.96 | 2 | 4 | 36.38 | 0.39 | -0.44 | 0.87 | 0.19 | 5.49 |
| MOL000546 | [diosgenin](https://old.tcmsp-e.com/molecule.php?qn=546) | 414.69 | 4.63 | 1 | 3 | 80.88 | 0.82 | 0.27 | 0.81 | 0.19 | 4.14 |
| MOL005461 | [Doradexanthin](https://old.tcmsp-e.com/molecule.php?qn=5461) | 584.96 | 8.73 | 2 | 3 | 38.16 | 0.52 | -1.19 | 0.54 | 0.34 | 4.13 |
| MOL005463 | [Methylcimicifugoside_qt](https://old.tcmsp-e.com/molecule.php?qn=5463) | 556.81 | 3.21 | 1 | 7 | 31.69 | 0.21 | -0.41 | 0.24 | 0.23 | 11.29 |
| MOL005465 | [AIDS180907](https://old.tcmsp-e.com/molecule.php?qn=5465) | 394.45 | 4.81 | 3 | 6 | 45.33 | 0.73 | 0 | 0.77 | 0.27 | 14.86 |
| MOL000953 | [CLR](https://old.tcmsp-e.com/molecule.php?qn=953) | 386.73 | 7.38 | 1 | 1 | 37.87 | 1.43 | 1.13 | 0.68 | 0.2 | 4.52 |

## 7.3 Phytochemical profile of Cornus officinalis Siebold & Zucc. Cornaceae (*shān zhū yú*)

| [Mol ID](https://old.tcmsp-e.com/tcmspsearch.php?qr=Cornus%20Officinalis%20Sieb.%20Et%20Zucc.&qsr=herb_en_name&token=314294d6a2fa9a6cea11d6eaec01ab12) | **Molecule Name** | **MW** | **AlogP** | **Hdon** | **Hacc** | **OB (%)** | **Caco-2** | **BBB** | **DL** | **FASA-** | **HL** |
| --- | --- | --- | --- | --- | --- | --- | --- | --- | --- | --- | --- |
| MOL001494 | [Mandenol](https://old.tcmsp-e.com/molecule.php?qn=1494) | 308.56 | 6.99 | 0 | 2 | 42 | 1.46 | 1.14 | 0.19 | 0.25 | 5.39 |
| MOL001495 | [Ethyl linolenate](https://old.tcmsp-e.com/molecule.php?qn=1495) | 306.54 | 6.55 | 0 | 2 | 46.1 | 1.54 | 1.12 | 0.2 | 0.25 | 6.2 |
| MOL001771 | [poriferast-5-en-3beta-ol](https://old.tcmsp-e.com/molecule.php?qn=1771) | 414.79 | 8.08 | 1 | 1 | 36.91 | 1.45 | 1.14 | 0.75 | 0 | 5.07 |
| MOL002879 | [Diop](https://old.tcmsp-e.com/molecule.php?qn=2879) | 390.62 | 7.44 | 0 | 4 | 43.59 | 0.79 | 0.26 | 0.39 | 0.28 | 3.6 |
| MOL002883 | [Ethyl oleate (NF)](https://old.tcmsp-e.com/molecule.php?qn=2883) | 310.58 | 7.44 | 0 | 2 | 32.4 | 1.4 | 1.1 | 0.19 | 0.19 | 4.85 |
| MOL003137 | [Leucanthoside](https://old.tcmsp-e.com/molecule.php?qn=3137) | 462.44 | -0.07 | 7 | 11 | 32.12 | -1.27 | -2.41 | 0.78 | 0 | 16.28 |
| MOL000358 | [beta-sitosterol](https://old.tcmsp-e.com/molecule.php?qn=358) | 414.79 | 8.08 | 1 | 1 | 36.91 | 1.32 | 0.99 | 0.75 | 0.23 | 5.36 |
| MOL000359 | [sitosterol](https://old.tcmsp-e.com/molecule.php?qn=359) | 414.79 | 8.08 | 1 | 1 | 36.91 | 1.32 | 0.87 | 0.75 | 0.22 | 5.37 |
| MOL000449 | [Stigmasterol](https://old.tcmsp-e.com/molecule.php?qn=449) | 412.77 | 7.64 | 1 | 1 | 43.83 | 1.44 | 1 | 0.76 | 0.22 | 5.57 |
| MOL005360 | [malkangunin](https://old.tcmsp-e.com/molecule.php?qn=5360) | 432.56 | 1.84 | 2 | 7 | 57.71 | 0.22 | -0.17 | 0.63 | 0.3 | 4.09 |
| MOL005481 | [2,6,10,14,18-pentamethylicosa-2,6,10,14,18-pentaene](https://old.tcmsp-e.com/molecule.php?qn=5481) | 342.67 | 9.51 | 0 | 0 | 33.4 | 1.94 | 1.99 | 0.24 | 0.29 | 6.05 |
| MOL005486 | [3,4-Dehydrolycopen-16-al](https://old.tcmsp-e.com/molecule.php?qn=5486) | 548.92 | 11.48 | 0 | 1 | 46.64 | 2 | 0.6 | 0.49 | 0.37 | 4.29 |
| MOL005489 | [3,6-Digalloylglucose](https://old.tcmsp-e.com/molecule.php?qn=5489) | 484.4 | -0.03 | 9 | 14 | 31.42 | -1.95 | -3.05 | 0.66 | 0.38 | 3.39 |
| MOL005503 | [Cornudentanone](https://old.tcmsp-e.com/molecule.php?qn=5503) | 378.56 | 4.97 | 0 | 5 | 39.66 | 0.47 | 0.09 | 0.33 | 0.21 | 2.83 |
| MOL005530 | [Hydroxygenkwanin](https://old.tcmsp-e.com/molecule.php?qn=5530) | 300.28 | 2.32 | 3 | 6 | 36.47 | 0.52 | -0.44 | 0.27 | 0.31 | 15.22 |
| MOL005531 | [Telocinobufagin](https://old.tcmsp-e.com/molecule.php?qn=5531) | 402.58 | 2.11 | 3 | 5 | 69.99 | -0.12 | -0.85 | 0.79 | 0.26 | 5.15 |
| MOL008457 | [Tetrahydroalstonine](https://old.tcmsp-e.com/molecule.php?qn=8457) | 352.47 | 2.66 | 1 | 4 | 32.42 | 0.9 | 0.33 | 0.81 | 0.23 | 10.55 |
| MOL000554 | [gallic acid-3-O-(6'-O-galloyl)-glucoside](https://old.tcmsp-e.com/molecule.php?qn=554) | 484.4 | -0.03 | 9 | 14 | 30.25 | -1.96 | -2.76 | 0.67 | 0.36 | 2.48 |
| MOL005552 | [gemin D](https://old.tcmsp-e.com/molecule.php?qn=5552) | 634.49 | 0.73 | 11 | 18 | 68.83 | -2.17 | -2.71 | 0.56 | 0.38 | 5.55 |
| MOL005557 | [lanosta-8,24-dien-3-ol,3-acetate](https://old.tcmsp-e.com/molecule.php?qn=5557) | 468.84 | 8.5 | 0 | 2 | 44.3 | 1.45 | 1.31 | 0.82 | 0.25 | 7.21 |

## 7.4 Phytochemical profile of Alisma plantago-aquatica L. Alismataceae (*zé xiè*)

| [Mol ID](https://old.tcmsp-e.com/tcmspsearch.php?qr=Alisma%20Orientale%20(Sam.)%20Juz.&qsr=herb_en_name&token=314294d6a2fa9a6cea11d6eaec01ab12) | **Molecule Name** | **MW** | **AlogP** | **Hdon** | **Hacc** | **OB (%)** | **Caco-2** | **BBB** | **DL** | **FASA-** | **HL** |
| --- | --- | --- | --- | --- | --- | --- | --- | --- | --- | --- | --- |
| MOL000359 | [sitosterol](https://old.tcmsp-e.com/molecule.php?qn=359) | 414.79 | 8.08 | 1 | 1 | 36.91 | 1.32 | 0.87 | 0.75 | 0.22 | 5.37 |
| MOL000830 | [Alisol B](https://old.tcmsp-e.com/molecule.php?qn=830) | 472.78 | 4.64 | 2 | 4 | 34.47 | 0.04 | -0.54 | 0.82 | 0.27 | 9.14 |
| MOL000831 | [Alisol B monoacetate](https://old.tcmsp-e.com/molecule.php?qn=831) | 514.82 | 5.02 | 1 | 5 | 35.58 | 0.46 | -0.18 | 0.81 | 0.26 | 10.97 |
| MOL000832 | [alisol,b,23-acetate](https://old.tcmsp-e.com/molecule.php?qn=832) | 446.74 | 3.82 | 2 | 4 | 32.52 | -0.06 | -0.94 | 0.82 | 0.27 | 7.65 |
| MOL000849 | [16β-methoxyalisol B monoacetate](https://old.tcmsp-e.com/molecule.php?qn=849) | 544.85 | 4.33 | 1 | 6 | 32.43 | 0.07 | -0.6 | 0.77 | 0.26 | 10.31 |
| MOL000853 | [alisol B](https://old.tcmsp-e.com/molecule.php?qn=853) | 444.72 | 3.76 | 2 | 4 | 36.76 | 0.07 | -0.67 | 0.82 | 0.26 | 7.35 |
| MOL000854 | [alisol C](https://old.tcmsp-e.com/molecule.php?qn=854) | 486.76 | 3.5 | 2 | 5 | 32.7 | -0.34 | -0.97 | 0.82 | 0.31 | 7.89 |
| MOL000856 | [alisol C monoacetate](https://old.tcmsp-e.com/molecule.php?qn=856) | 514.77 | 3.67 | 1 | 6 | 33.06 | -0.18 | -0.73 | 0.83 | 0.32 | 10.43 |
| MOL002464 | [1-Monolinolein](https://old.tcmsp-e.com/molecule.php?qn=2464) | 354.59 | 5.59 | 2 | 4 | 37.18 | 0.32 | -0.32 | 0.3 | 0.23 | 4.36 |
| MOL000862 | [[(1S,3R)-1-[(2R)-3,3-dimethyloxiran-2-yl]-3-[(5R,8S,9S,10S,11S,14R)-11-hydroxy-4,4,8,10,14-pentamethyl-3-oxo-1,2,5,6,7,9,11,12,15,16-decahydrocyclopenta[a]phenanthren-17-yl]butyl] acetate](https://old.tcmsp-e.com/molecule.php?qn=862) | 514.82 | 5.02 | 1 | 5 | 35.58 | 0.34 | -0.27 | 0.81 | 0.29 | 12.04 |

## 7.5 Phytochemical profile of Smilax glabra Roxb. Smilacaceae (*fú líng*)

| [Mol ID](https://old.tcmsp-e.com/tcmspsearch.php?qr=Poria%20Cocos(Schw.)%20Wolf.&qsr=herb_en_name&token=314294d6a2fa9a6cea11d6eaec01ab12) | **Molecule Name** | **MW** | **AlogP** | **Hdon** | **Hacc** | **OB (%)** | **Caco-2** | **BBB** | **DL** | **FASA-** | **HL** |
| --- | --- | --- | --- | --- | --- | --- | --- | --- | --- | --- | --- |
| MOL000273 | [(2R)-2-[(3S,5R,10S,13R,14R,16R,17R)-3,16-dihydroxy-4,4,10,13,14-pentamethyl-2,3,5,6,12,15,16,17-octahydro-1H-cyclopenta[a]phenanthren-17-yl]-6-methylhept-5-enoic acid](https://old.tcmsp-e.com/molecule.php?qn=273) | 470.76 | 5.41 | 3 | 4 | 30.93 | 0.01 | -0.76 | 0.81 | 0 | 6.81 |
| MOL000275 | [trametenolic acid](https://old.tcmsp-e.com/molecule.php?qn=275) | 456.78 | 7.03 | 2 | 3 | 38.71 | 0.52 | -0.14 | 0.8 | 0 | 7.78 |
| MOL000276 | [7,9(11)-dehydropachymic acid](https://old.tcmsp-e.com/molecule.php?qn=276) | 526.83 | 6.1 | 2 | 5 | 35.11 | 0.03 | -0.87 | 0.81 | 0 | 7.34 |
| MOL000279 | [Cerevisterol](https://old.tcmsp-e.com/molecule.php?qn=279) | 430.74 | 5.15 | 3 | 3 | 37.96 | 0.28 | -0.39 | 0.77 | 0 | 5.31 |
| MOL000280 | [(2R)-2-[(3S,5R,10S,13R,14R,16R,17R)-3,16-dihydroxy-4,4,10,13,14-pentamethyl-2,3,5,6,12,15,16,17-octahydro-1H-cyclopenta[a]phenanthren-17-yl]-5-isopropyl-hex-5-enoic acid](https://old.tcmsp-e.com/molecule.php?qn=280) | 484.79 | 5.72 | 3 | 4 | 31.07 | 0.05 | -0.7 | 0.82 | 0 | 7.42 |
| MOL000282 | [ergosta-7,22E-dien-3beta-ol](https://old.tcmsp-e.com/molecule.php?qn=282) | 398.74 | 7.18 | 1 | 1 | 43.51 | 1.32 | 0.91 | 0.72 | 0 | 5.11 |
| MOL000283 | [Ergosterol peroxide](https://old.tcmsp-e.com/molecule.php?qn=283) | 430.74 | 7.17 | 1 | 3 | 40.36 | 0.84 | 0.34 | 0.81 | 0 | 3.43 |
| MOL000285 | [(2R)-2-[(5R,10S,13R,14R,16R,17R)-16-hydroxy-3-keto-4,4,10,13,14-pentamethyl-1,2,5,6,12,15,16,17-octahydrocyclopenta[a]phenanthren-17-yl]-5-isopropyl-hex-5-enoic acid](https://old.tcmsp-e.com/molecule.php?qn=285) | 482.77 | 5.68 | 2 | 4 | 38.26 | 0.12 | -0.57 | 0.82 | 0 | 6.77 |
| MOL000287 | [3beta-Hydroxy-24-methylene-8-lanostene-21-oic acid](https://old.tcmsp-e.com/molecule.php?qn=287) | 470.81 | 7.33 | 2 | 3 | 38.7 | 0.61 | -0.04 | 0.81 | 0 | 6.59 |
| MOL000289 | [pachymic acid](https://old.tcmsp-e.com/molecule.php?qn=289) | 528.85 | 6.54 | 2 | 5 | 33.63 | 0.1 | -0.57 | 0.81 | 0 | 9.27 |
| MOL000290 | [Poricoic acid A](https://old.tcmsp-e.com/molecule.php?qn=290) | 498.77 | 5.94 | 3 | 5 | 30.61 | -0.14 | -0.93 | 0.76 | 0 | 8.26 |
| MOL000291 | [Poricoic acid B](https://old.tcmsp-e.com/molecule.php?qn=291) | 484.74 | 5.64 | 3 | 5 | 30.52 | -0.08 | -0.87 | 0.75 | 0 | 8.67 |
| MOL000292 | [poricoic acid C](https://old.tcmsp-e.com/molecule.php?qn=292) | 482.77 | 7.11 | 2 | 4 | 38.15 | 0.32 | -0.41 | 0.75 | 0 | 7.73 |
| MOL000296 | [hederagenin](https://old.tcmsp-e.com/molecule.php?qn=296) | 414.79 | 8.08 | 1 | 1 | 36.91 | 1.32 | 0.96 | 0.75 | 0 | 5.35 |
| MOL000300 | [dehydroeburicoic acid](https://old.tcmsp-e.com/molecule.php?qn=300) | 453.75 | 6.35 | 1 | 3 | 44.17 | 0.38 | -0.16 | 0.83 | 0.04 | 7.04 |

## 7.6 Phytochemical profile of Paeonia × suffruticosa Andrews. Paeoniaceae (*mŭ dān pí*)

| [Mol ID](https://old.tcmsp-e.com/tcmspsearch.php?qr=Cortex%20Moutan&qsr=herb_en_name&token=314294d6a2fa9a6cea11d6eaec01ab12) | **Molecule Name** | **MW** | **AlogP** | **Hdon** | **Hacc** | **OB (%)** | **Caco-2** | **BBB** | **DL** | **FASA-** | **HL** |
| --- | --- | --- | --- | --- | --- | --- | --- | --- | --- | --- | --- |
| MOL001925 | [paeoniflorin_qt](https://old.tcmsp-e.com/molecule.php?qn=1925) | 318.35 | 0.46 | 2 | 6 | 68.18 | -0.34 | -0.73 | 0.4 | 0.39 | 8.81 |
| MOL000211 | [Mairin](https://old.tcmsp-e.com/molecule.php?qn=211) | 456.78 | 6.52 | 2 | 3 | 55.38 | 0.73 | 0.22 | 0.78 | 0.26 | 8.87 |
| MOL000359 | [sitosterol](https://old.tcmsp-e.com/molecule.php?qn=359) | 414.79 | 8.08 | 1 | 1 | 36.91 | 1.32 | 0.87 | 0.75 | 0.22 | 5.37 |
| MOL000422 | [kaempferol](https://old.tcmsp-e.com/molecule.php?qn=422) | 286.25 | 1.77 | 4 | 6 | 41.88 | 0.26 | -0.55 | 0.24 | 0 | 14.74 |
| MOL000492 | [(+)-catechin](https://old.tcmsp-e.com/molecule.php?qn=492) | 290.29 | 1.92 | 5 | 6 | 54.83 | -0.03 | -0.73 | 0.24 | 0 | 0.61 |
| MOL007003 | [benzoyl paeoniflorin](https://old.tcmsp-e.com/molecule.php?qn=7003) | 584.62 | 0.76 | 4 | 12 | 31.14 | -1.35 | -2.08 | 0.54 | 0.4 | 15.66 |
| MOL007369 | [4-O-methylpaeoniflorin_qt](https://old.tcmsp-e.com/molecule.php?qn=7369) | 332.38 | 0.87 | 1 | 6 | 67.24 | 0.15 | -0.15 | 0.43 | 0.34 | 9.51 |
| MOL007374 | [5-[[5-(4-methoxyphenyl)-2-furyl]methylene]barbituric acid](https://old.tcmsp-e.com/molecule.php?qn=7374) | 312.3 | 2.05 | 2 | 7 | 43.44 | 0.09 | -0.7 | 0.3 | 0.29 | 2.59 |
| MOL007382 | [mudanpioside-h_qt 2](https://old.tcmsp-e.com/molecule.php?qn=7382) | 336.37 | -0.03 | 3 | 7 | 42.36 | -0.39 | -0.96 | 0.37 | 0.32 | 7.6 |
| MOL007384 | [paeonidanin_qt](https://old.tcmsp-e.com/molecule.php?qn=7384) | 330.41 | 0.92 | 1 | 5 | 65.31 | -0.09 | -0.59 | 0.35 | 0.35 | 7.1 |
| MOL000098 | [quercetin](https://old.tcmsp-e.com/molecule.php?qn=98) | 302.25 | 1.5 | 5 | 7 | 46.43 | 0.05 | -0.77 | 0.28 | 0.38 | 14.4 |

## 7.7 Phytochemical profile of Neolitsea cassia (L.) Kosterm. Lauraceae (*guì zhī*)

| [Mol ID](https://old.tcmsp-e.com/tcmspsearch.php?qr=Cinnamomi%20Ramulus&qsr=herb_en_name&token=314294d6a2fa9a6cea11d6eaec01ab12) | **Molecule Name** | **MW** | **AlogP** | **Hdon** | **Hacc** | **OB (%)** | **Caco-2** | **BBB** | **DL** | **FASA-** | **HL** |
| --- | --- | --- | --- | --- | --- | --- | --- | --- | --- | --- | --- |
| MOL001736 | [(-)-taxifolin](https://old.tcmsp-e.com/molecule.php?qn=1736) | 304.27 | 1.49 | 5 | 7 | 60.51 | -0.24 | -1.02 | 0.27 | 0.41 | 14.37 |
| MOL000358 | [beta-sitosterol](https://old.tcmsp-e.com/molecule.php?qn=358) | 414.79 | 8.08 | 1 | 1 | 36.91 | 1.32 | 0.99 | 0.75 | 0.23 | 5.36 |
| MOL000359 | [sitosterol](https://old.tcmsp-e.com/molecule.php?qn=359) | 414.79 | 8.08 | 1 | 1 | 36.91 | 1.32 | 0.87 | 0.75 | 0.22 | 5.37 |
| MOL000492 | [(+)-catechin](https://old.tcmsp-e.com/molecule.php?qn=492) | 290.29 | 1.92 | 5 | 6 | 54.83 | -0.03 | -0.73 | 0.24 | 0 | 0.61 |
| MOL000073 | [ent-Epicatechin](https://old.tcmsp-e.com/molecule.php?qn=73) | 290.29 | 1.92 | 5 | 6 | 48.96 | 0.02 | -0.64 | 0.24 | 0.34 | 0.63 |
| MOL004576 | [taxifolin](https://old.tcmsp-e.com/molecule.php?qn=4576) | 304.27 | 1.49 | 5 | 7 | 57.84 | -0.23 | -0.8 | 0.27 | 0.39 | 14.41 |
| MOL011169 | [Peroxyergosterol](https://old.tcmsp-e.com/molecule.php?qn=11169) | 428.72 | 6.73 | 1 | 3 | 44.39 | 0.86 | 0.43 | 0.82 | 0.24 | 4.06 |

## 7.8 Phytochemical profile of Aconitum carmichaeli Debeaux. Ranunculaceae (*zhì fù zĭ*)

| [Mol ID](https://old.tcmsp-e.com/tcmspsearch.php?qr=Aconiti%20Lateralis%20Radix%20Praeparata&qsr=herb_en_name&token=314294d6a2fa9a6cea11d6eaec01ab12) | **Molecule Name** | **MW** | **AlogP** | **Hdon** | **Hacc** | **OB (%)** | **Caco-2** | **BBB** | **DL** | **FASA-** | **HL** |
| --- | --- | --- | --- | --- | --- | --- | --- | --- | --- | --- | --- |
| MOL002211 | [11,14-eicosadienoic acid](https://old.tcmsp-e.com/molecule.php?qn=2211) | 308.56 | 7.3 | 1 | 2 | 39.99 | 1.22 | 0.76 | 0.2 | 0.23 | 5.6 |
| MOL002388 | [Delphin_qt](https://old.tcmsp-e.com/molecule.php?qn=2388) | 303.26 | 1.67 | 6 | 7 | 57.76 | 0.12 | -0.66 | 0.28 | 0.35 | 1.43 |
| MOL002392 | [Deltoin](https://old.tcmsp-e.com/molecule.php?qn=2392) | 328.39 | 2.48 | 0 | 5 | 46.69 | 0.55 | -0.12 | 0.37 | 0.32 | 7.7 |
| MOL002393 | [Demethyldelavaine A](https://old.tcmsp-e.com/molecule.php?qn=2393) | 700.91 | -0.12 | 4 | 13 | 34.52 | -0.69 | -1.19 | 0.18 | 0.23 | 26.05 |
| MOL002394 | [Demethyldelavaine B](https://old.tcmsp-e.com/molecule.php?qn=2394) | 700.91 | -0.12 | 4 | 13 | 34.52 | -0.7 | -1.01 | 0.18 | 0.22 | 26.75 |
| MOL002395 | [Deoxyandrographolide](https://old.tcmsp-e.com/molecule.php?qn=2395) | 334.5 | 3.02 | 2 | 4 | 56.3 | 0.18 | -0.49 | 0.31 | 0.27 | 2.79 |
| MOL002397 | [karakoline](https://old.tcmsp-e.com/molecule.php?qn=2397) | 377.58 | -0.05 | 3 | 5 | 51.73 | 0.32 | -0.03 | 0.73 | 0.15 | 11.1 |
| MOL002398 | [Karanjin](https://old.tcmsp-e.com/molecule.php?qn=2398) | 292.3 | 2.94 | 0 | 4 | 69.56 | 1.22 | 0.62 | 0.34 | 0.26 | 13.15 |
| MOL002401 | [Neokadsuranic acid B](https://old.tcmsp-e.com/molecule.php?qn=2401) | 452.74 | 7.05 | 1 | 3 | 43.1 | 0.69 | -0.01 | 0.85 | 0.29 | 12.05 |
| MOL002406 | [2,7-Dideacetyl-2,7-dibenzoyl-taxayunnanine F](https://old.tcmsp-e.com/molecule.php?qn=2406) | 776.9 | 3.12 | 1 | 14 | 39.43 | -0.75 | -1.3 | 0.38 | 0.38 | 10.95 |
| MOL002410 | [benzoylnapelline](https://old.tcmsp-e.com/molecule.php?qn=2410) | 463.67 | 3.12 | 2 | 5 | 34.06 | 0.19 | -0.38 | 0.53 | 0.31 | 15.72 |
| MOL002415 | [6-Demethyldesoline](https://old.tcmsp-e.com/molecule.php?qn=2415) | 453.64 | -1.97 | 4 | 8 | 51.87 | -0.26 | -0.57 | 0.66 | 0.12 | 13.14 |
| MOL002416 | [deoxyaconitine](https://old.tcmsp-e.com/molecule.php?qn=2416) | 629.82 | 0.25 | 2 | 11 | 30.96 | -0.23 | -0.61 | 0.24 | 0.19 | 22.64 |
| MOL002419 | [(R)-Norcoclaurine](https://old.tcmsp-e.com/molecule.php?qn=2419) | 271.34 | 2.57 | 4 | 4 | 82.54 | 0.63 | 0.03 | 0.21 | 0.34 | 3.85 |
| MOL002421 | [ignavine](https://old.tcmsp-e.com/molecule.php?qn=2421) | 449.59 | 1.22 | 3 | 6 | 84.08 | -0.07 | -0.49 | 0.25 | 0.32 | 28.93 |
| MOL002422 | [isotalatizidine](https://old.tcmsp-e.com/molecule.php?qn=2422) | 407.61 | -0.73 | 3 | 6 | 50.82 | -0.11 | -0.62 | 0.73 | 0.14 | 11.59 |
| MOL002423 | [jesaconitine](https://old.tcmsp-e.com/molecule.php?qn=2423) | 675.85 | -0.99 | 3 | 13 | 33.41 | -0.44 | -1.16 | 0.19 | 0.19 | 26.06 |
| MOL002433 | [(3R,8S,9R,10R,13R,14S,17R)-3-hydroxy-4,4,9,13,14-pentamethyl-17-[(E,2R)-6-methyl-7-[(2R,3R,4S,5S,6R)-3,4,5-trihydroxy-6-[[(2R,3R,4S,5S,6R)-3,4,5-trihydroxy-6-(hydroxymethyl)oxan-2-yl]oxymethyl]oxan-2-yl]oxyhept-5-en-2-yl]-1,2,3,7,8,10,12,15,16,17-decahydr](https://old.tcmsp-e.com/molecule.php?qn=2433) | 781.1 | 2.2 | 8 | 13 | 41.52 | -2.31 | -3.18 | 0.22 | 0.25 | 10.51 |
| MOL002434 | [Carnosifloside I_qt](https://old.tcmsp-e.com/molecule.php?qn=2434) | 456.78 | 5.69 | 2 | 3 | 38.16 | 0.28 | -0.77 | 0.8 | 0.25 | 7 |
| MOL000359 | [sitosterol](https://old.tcmsp-e.com/molecule.php?qn=359) | 414.79 | 8.08 | 1 | 1 | 36.91 | 1.32 | 0.87 | 0.75 | 0.22 | 5.37 |
| MOL000538 | [hypaconitine](https://old.tcmsp-e.com/molecule.php?qn=538) | 615.79 | -0.1 | 2 | 11 | 31.39 | -0.34 | -0.78 | 0.26 | 0.21 | 19.87 |

## 7.9 Phytochemical profile of JGSQW

| [Mol ID](https://old.tcmsp-e.com/tcmspsearch.php?qr=Rehmanniae%20Radix%20Praeparata&qsr=herb_en_name&token=314294d6a2fa9a6cea11d6eaec01ab12) | **Molecule Name** | **MW** | **AlogP** | **Hdon** | **Hacc** | **OB (%)** | **Caco-2** | **BBB** | **DL** | **FASA-** | **HL** |
| --- | --- | --- | --- | --- | --- | --- | --- | --- | --- | --- | --- |
| MOL000359 | [sitosterol](https://old.tcmsp-e.com/molecule.php?qn=359) | 414.79 | 8.08 | 1 | 1 | 36.91 | 1.32 | 0.87 | 0.75 | 0.22 | 5.37 |
| MOL000449 | [Stigmasterol](https://old.tcmsp-e.com/molecule.php?qn=449) | 412.77 | 7.64 | 1 | 1 | 43.83 | 1.44 | 1 | 0.76 | 0.22 | 5.57 |
| MOL001494 | [Mandenol](https://old.tcmsp-e.com/molecule.php?qn=1494) | 308.56 | 6.99 | 0 | 2 | 42 | 1.46 | 1.14 | 0.19 | 0.25 | 5.39 |
| MOL001495 | [Ethyl linolenate](https://old.tcmsp-e.com/molecule.php?qn=1495) | 306.54 | 6.55 | 0 | 2 | 46.1 | 1.54 | 1.12 | 0.2 | 0.25 | 6.2 |
| MOL001771 | [poriferast-5-en-3beta-ol](https://old.tcmsp-e.com/molecule.php?qn=1771) | 414.79 | 8.08 | 1 | 1 | 36.91 | 1.45 | 1.14 | 0.75 | 0 | 5.07 |
| MOL002879 | [Diop](https://old.tcmsp-e.com/molecule.php?qn=2879) | 390.62 | 7.44 | 0 | 4 | 43.59 | 0.79 | 0.26 | 0.39 | 0.28 | 3.6 |
| MOL002883 | [Ethyl oleate (NF)](https://old.tcmsp-e.com/molecule.php?qn=2883) | 310.58 | 7.44 | 0 | 2 | 32.4 | 1.4 | 1.1 | 0.19 | 0.19 | 4.85 |
| MOL003137 | [Leucanthoside](https://old.tcmsp-e.com/molecule.php?qn=3137) | 462.44 | -0.07 | 7 | 11 | 32.12 | -1.27 | -2.41 | 0.78 | 0 | 16.28 |
| MOL000358 | [beta-sitosterol](https://old.tcmsp-e.com/molecule.php?qn=358) | 414.79 | 8.08 | 1 | 1 | 36.91 | 1.32 | 0.99 | 0.75 | 0.23 | 5.36 |
| MOL005360 | [malkangunin](https://old.tcmsp-e.com/molecule.php?qn=5360) | 432.56 | 1.84 | 2 | 7 | 57.71 | 0.22 | -0.17 | 0.63 | 0.3 | 4.09 |
| MOL005481 | [2,6,10,14,18-pentamethylicosa-2,6,10,14,18-pentaene](https://old.tcmsp-e.com/molecule.php?qn=5481) | 342.67 | 9.51 | 0 | 0 | 33.4 | 1.94 | 1.99 | 0.24 | 0.29 | 6.05 |
| MOL005486 | [3,4-Dehydrolycopen-16-al](https://old.tcmsp-e.com/molecule.php?qn=5486) | 548.92 | 11.48 | 0 | 1 | 46.64 | 2 | 0.6 | 0.49 | 0.37 | 4.29 |
| MOL005489 | [3,6-Digalloylglucose](https://old.tcmsp-e.com/molecule.php?qn=5489) | 484.4 | -0.03 | 9 | 14 | 31.42 | -1.95 | -3.05 | 0.66 | 0.38 | 3.39 |
| MOL005503 | [Cornudentanone](https://old.tcmsp-e.com/molecule.php?qn=5503) | 378.56 | 4.97 | 0 | 5 | 39.66 | 0.47 | 0.09 | 0.33 | 0.21 | 2.83 |
| MOL005530 | [Hydroxygenkwanin](https://old.tcmsp-e.com/molecule.php?qn=5530) | 300.28 | 2.32 | 3 | 6 | 36.47 | 0.52 | -0.44 | 0.27 | 0.31 | 15.22 |
| MOL005531 | [Telocinobufagin](https://old.tcmsp-e.com/molecule.php?qn=5531) | 402.58 | 2.11 | 3 | 5 | 69.99 | -0.12 | -0.85 | 0.79 | 0.26 | 5.15 |
| MOL008457 | [Tetrahydroalstonine](https://old.tcmsp-e.com/molecule.php?qn=8457) | 352.47 | 2.66 | 1 | 4 | 32.42 | 0.9 | 0.33 | 0.81 | 0.23 | 10.55 |
| MOL000554 | [gallic acid-3-O-(6'-O-galloyl)-glucoside](https://old.tcmsp-e.com/molecule.php?qn=554) | 484.4 | -0.03 | 9 | 14 | 30.25 | -1.96 | -2.76 | 0.67 | 0.36 | 2.48 |
| MOL005552 | [gemin D](https://old.tcmsp-e.com/molecule.php?qn=5552) | 634.49 | 0.73 | 11 | 18 | 68.83 | -2.17 | -2.71 | 0.56 | 0.38 | 5.55 |
| MOL005557 | [lanosta-8,24-dien-3-ol,3-acetate](https://old.tcmsp-e.com/molecule.php?qn=5557) | 468.84 | 8.5 | 0 | 2 | 44.3 | 1.45 | 1.31 | 0.82 | 0.25 | 7.21 |
| MOL001559 | [piperlonguminine](https://old.tcmsp-e.com/molecule.php?qn=1559) | 273.36 | 2.93 | 1 | 4 | 30.71 | 0.95 | 0.27 | 0.18 | 0.35 | 8.66 |
| MOL001736 | [(-)-taxifolin](https://old.tcmsp-e.com/molecule.php?qn=1736) | 304.27 | 1.49 | 5 | 7 | 60.51 | -0.24 | -1.02 | 0.27 | 0.41 | 14.37 |
| MOL000310 | [Denudatin B](https://old.tcmsp-e.com/molecule.php?qn=310) | 356.45 | 2.8 | 0 | 5 | 61.47 | 0.9 | 0.35 | 0.38 | 0.24 | 7.71 |
| MOL000322 | [Kadsurenone](https://old.tcmsp-e.com/molecule.php?qn=322) | 356.45 | 2.8 | 0 | 5 | 54.72 | 0.82 | 0.52 | 0.38 | 0.24 | 9.16 |
| MOL005429 | [hancinol](https://old.tcmsp-e.com/molecule.php?qn=5429) | 372.5 | 2.46 | 1 | 5 | 64.01 | 0.53 | 0.17 | 0.37 | 0.25 | 4.06 |
| MOL005430 | [hancinone C](https://old.tcmsp-e.com/molecule.php?qn=5430) | 400.51 | 3.34 | 0 | 6 | 59.05 | 0.74 | 0.06 | 0.39 | 0.19 | 4.14 |
| MOL005435 | [24-Methylcholest-5-enyl-3belta-O-glucopyranoside_qt](https://old.tcmsp-e.com/molecule.php?qn=5435) | 400.76 | 7.63 | 1 | 1 | 37.58 | 1.33 | 0.96 | 0.72 | 0.22 | 4.91 |
| MOL005438 | [campesterol](https://old.tcmsp-e.com/molecule.php?qn=5438) | 400.76 | 7.63 | 1 | 1 | 37.58 | 1.34 | 0.95 | 0.71 | 0.22 | 4.83 |
| MOL005440 | [Isofucosterol](https://old.tcmsp-e.com/molecule.php?qn=5440) | 412.77 | 7.83 | 1 | 1 | 43.78 | 1.36 | 0.97 | 0.76 | 0.24 | 5.18 |
| MOL005458 | [Dioscoreside C_qt](https://old.tcmsp-e.com/molecule.php?qn=5458) | 444.72 | 3.96 | 2 | 4 | 36.38 | 0.39 | -0.44 | 0.87 | 0.19 | 5.49 |
| MOL000546 | [diosgenin](https://old.tcmsp-e.com/molecule.php?qn=546) | 414.69 | 4.63 | 1 | 3 | 80.88 | 0.82 | 0.27 | 0.81 | 0.19 | 4.14 |
| MOL005461 | [Doradexanthin](https://old.tcmsp-e.com/molecule.php?qn=5461) | 584.96 | 8.73 | 2 | 3 | 38.16 | 0.52 | -1.19 | 0.54 | 0.34 | 4.13 |
| MOL005463 | [Methylcimicifugoside_qt](https://old.tcmsp-e.com/molecule.php?qn=5463) | 556.81 | 3.21 | 1 | 7 | 31.69 | 0.21 | -0.41 | 0.24 | 0.23 | 11.29 |
| MOL005465 | [AIDS180907](https://old.tcmsp-e.com/molecule.php?qn=5465) | 394.45 | 4.81 | 3 | 6 | 45.33 | 0.73 | 0 | 0.77 | 0.27 | 14.86 |
| MOL000953 | [CLR](https://old.tcmsp-e.com/molecule.php?qn=953) | 386.73 | 7.38 | 1 | 1 | 37.87 | 1.43 | 1.13 | 0.68 | 0.2 | 4.52 |
| MOL000830 | [Alisol B](https://old.tcmsp-e.com/molecule.php?qn=830) | 472.78 | 4.64 | 2 | 4 | 34.47 | 0.04 | -0.54 | 0.82 | 0.27 | 9.14 |
| MOL000831 | [Alisol B monoacetate](https://old.tcmsp-e.com/molecule.php?qn=831) | 514.82 | 5.02 | 1 | 5 | 35.58 | 0.46 | -0.18 | 0.81 | 0.26 | 10.97 |
| MOL000832 | [alisol,b,23-acetate](https://old.tcmsp-e.com/molecule.php?qn=832) | 446.74 | 3.82 | 2 | 4 | 32.52 | -0.06 | -0.94 | 0.82 | 0.27 | 7.65 |
| MOL000849 | [16β-methoxyalisol B monoacetate](https://old.tcmsp-e.com/molecule.php?qn=849) | 544.85 | 4.33 | 1 | 6 | 32.43 | 0.07 | -0.6 | 0.77 | 0.26 | 10.31 |
| MOL000854 | [alisol C](https://old.tcmsp-e.com/molecule.php?qn=854) | 486.76 | 3.5 | 2 | 5 | 32.7 | -0.34 | -0.97 | 0.82 | 0.31 | 7.89 |
| MOL000856 | [alisol C monoacetate](https://old.tcmsp-e.com/molecule.php?qn=856) | 514.77 | 3.67 | 1 | 6 | 33.06 | -0.18 | -0.73 | 0.83 | 0.32 | 10.43 |
| MOL002464 | [1-Monolinolein](https://old.tcmsp-e.com/molecule.php?qn=2464) | 354.59 | 5.59 | 2 | 4 | 37.18 | 0.32 | -0.32 | 0.3 | 0.23 | 4.36 |
| MOL000862 | [[(1S,3R)-1-[(2R)-3,3-dimethyloxiran-2-yl]-3-[(5R,8S,9S,10S,11S,14R)-11-hydroxy-4,4,8,10,14-pentamethyl-3-oxo-1,2,5,6,7,9,11,12,15,16-decahydrocyclopenta[a]phenanthren-17-yl]butyl] acetate](https://old.tcmsp-e.com/molecule.php?qn=862) | 514.82 | 5.02 | 1 | 5 | 35.58 | 0.34 | -0.27 | 0.81 | 0.29 | 12.04 |
| MOL000273 | [(2R)-2-[(3S,5R,10S,13R,14R,16R,17R)-3,16-dihydroxy-4,4,10,13,14-pentamethyl-2,3,5,6,12,15,16,17-octahydro-1H-cyclopenta[a]phenanthren-17-yl]-6-methylhept-5-enoic acid](https://old.tcmsp-e.com/molecule.php?qn=273) | 470.76 | 5.41 | 3 | 4 | 30.93 | 0.01 | -0.76 | 0.81 | 0 | 6.81 |
| MOL000275 | [trametenolic acid](https://old.tcmsp-e.com/molecule.php?qn=275) | 456.78 | 7.03 | 2 | 3 | 38.71 | 0.52 | -0.14 | 0.8 | 0 | 7.78 |
| MOL000276 | [7,9(11)-dehydropachymic acid](https://old.tcmsp-e.com/molecule.php?qn=276) | 526.83 | 6.1 | 2 | 5 | 35.11 | 0.03 | -0.87 | 0.81 | 0 | 7.34 |
| MOL000279 | [Cerevisterol](https://old.tcmsp-e.com/molecule.php?qn=279) | 430.74 | 5.15 | 3 | 3 | 37.96 | 0.28 | -0.39 | 0.77 | 0 | 5.31 |
| MOL000280 | [(2R)-2-[(3S,5R,10S,13R,14R,16R,17R)-3,16-dihydroxy-4,4,10,13,14-pentamethyl-2,3,5,6,12,15,16,17-octahydro-1H-cyclopenta[a]phenanthren-17-yl]-5-isopropyl-hex-5-enoic acid](https://old.tcmsp-e.com/molecule.php?qn=280) | 484.79 | 5.72 | 3 | 4 | 31.07 | 0.05 | -0.7 | 0.82 | 0 | 7.42 |
| MOL000282 | [ergosta-7,22E-dien-3beta-ol](https://old.tcmsp-e.com/molecule.php?qn=282) | 398.74 | 7.18 | 1 | 1 | 43.51 | 1.32 | 0.91 | 0.72 | 0 | 5.11 |
| MOL000283 | [Ergosterol peroxide](https://old.tcmsp-e.com/molecule.php?qn=283) | 430.74 | 7.17 | 1 | 3 | 40.36 | 0.84 | 0.34 | 0.81 | 0 | 3.43 |
| MOL000285 | [(2R)-2-[(5R,10S,13R,14R,16R,17R)-16-hydroxy-3-keto-4,4,10,13,14-pentamethyl-1,2,5,6,12,15,16,17-octahydrocyclopenta[a]phenanthren-17-yl]-5-isopropyl-hex-5-enoic acid](https://old.tcmsp-e.com/molecule.php?qn=285) | 482.77 | 5.68 | 2 | 4 | 38.26 | 0.12 | -0.57 | 0.82 | 0 | 6.77 |
| MOL000287 | [3beta-Hydroxy-24-methylene-8-lanostene-21-oic acid](https://old.tcmsp-e.com/molecule.php?qn=287) | 470.81 | 7.33 | 2 | 3 | 38.7 | 0.61 | -0.04 | 0.81 | 0 | 6.59 |
| MOL000289 | [pachymic acid](https://old.tcmsp-e.com/molecule.php?qn=289) | 528.85 | 6.54 | 2 | 5 | 33.63 | 0.1 | -0.57 | 0.81 | 0 | 9.27 |
| MOL000290 | [Poricoic acid A](https://old.tcmsp-e.com/molecule.php?qn=290) | 498.77 | 5.94 | 3 | 5 | 30.61 | -0.14 | -0.93 | 0.76 | 0 | 8.26 |
| MOL000291 | [Poricoic acid B](https://old.tcmsp-e.com/molecule.php?qn=291) | 484.74 | 5.64 | 3 | 5 | 30.52 | -0.08 | -0.87 | 0.75 | 0 | 8.67 |
| MOL000292 | [poricoic acid C](https://old.tcmsp-e.com/molecule.php?qn=292) | 482.77 | 7.11 | 2 | 4 | 38.15 | 0.32 | -0.41 | 0.75 | 0 | 7.73 |
| MOL000296 | [hederagenin](https://old.tcmsp-e.com/molecule.php?qn=296) | 414.79 | 8.08 | 1 | 1 | 36.91 | 1.32 | 0.96 | 0.75 | 0 | 5.35 |
| MOL000300 | [dehydroeburicoic acid](https://old.tcmsp-e.com/molecule.php?qn=300) | 453.75 | 6.35 | 1 | 3 | 44.17 | 0.38 | -0.16 | 0.83 | 0.04 | 7.04 |
| MOL001925 | [paeoniflorin_qt](https://old.tcmsp-e.com/molecule.php?qn=1925) | 318.35 | 0.46 | 2 | 6 | 68.18 | -0.34 | -0.73 | 0.4 | 0.39 | 8.81 |
| MOL000211 | [Mairin](https://old.tcmsp-e.com/molecule.php?qn=211) | 456.78 | 6.52 | 2 | 3 | 55.38 | 0.73 | 0.22 | 0.78 | 0.26 | 8.87 |
| MOL000422 | [kaempferol](https://old.tcmsp-e.com/molecule.php?qn=422) | 286.25 | 1.77 | 4 | 6 | 41.88 | 0.26 | -0.55 | 0.24 | 0 | 14.74 |
| MOL000492 | [(+)-catechin](https://old.tcmsp-e.com/molecule.php?qn=492) | 290.29 | 1.92 | 5 | 6 | 54.83 | -0.03 | -0.73 | 0.24 | 0 | 0.61 |
| MOL007003 | [benzoyl paeoniflorin](https://old.tcmsp-e.com/molecule.php?qn=7003) | 584.62 | 0.76 | 4 | 12 | 31.14 | -1.35 | -2.08 | 0.54 | 0.4 | 15.66 |
| MOL007369 | [4-O-methylpaeoniflorin_qt](https://old.tcmsp-e.com/molecule.php?qn=7369) | 332.38 | 0.87 | 1 | 6 | 67.24 | 0.15 | -0.15 | 0.43 | 0.34 | 9.51 |
| MOL007374 | [5-[[5-(4-methoxyphenyl)-2-furyl]methylene]barbituric acid](https://old.tcmsp-e.com/molecule.php?qn=7374) | 312.3 | 2.05 | 2 | 7 | 43.44 | 0.09 | -0.7 | 0.3 | 0.29 | 2.59 |
| MOL007382 | [mudanpioside-h_qt 2](https://old.tcmsp-e.com/molecule.php?qn=7382) | 336.37 | -0.03 | 3 | 7 | 42.36 | -0.39 | -0.96 | 0.37 | 0.32 | 7.6 |
| MOL007384 | [paeonidanin_qt](https://old.tcmsp-e.com/molecule.php?qn=7384) | 330.41 | 0.92 | 1 | 5 | 65.31 | -0.09 | -0.59 | 0.35 | 0.35 | 7.1 |
| MOL000098 | [quercetin](https://old.tcmsp-e.com/molecule.php?qn=98) | 302.25 | 1.5 | 5 | 7 | 46.43 | 0.05 | -0.77 | 0.28 | 0.38 | 14.4 |
| MOL002211 | [11,14-eicosadienoic acid](https://old.tcmsp-e.com/molecule.php?qn=2211) | 308.56 | 7.3 | 1 | 2 | 39.99 | 1.22 | 0.76 | 0.2 | 0.23 | 5.6 |
| MOL002388 | [Delphin_qt](https://old.tcmsp-e.com/molecule.php?qn=2388) | 303.26 | 1.67 | 6 | 7 | 57.76 | 0.12 | -0.66 | 0.28 | 0.35 | 1.43 |
| MOL002392 | [Deltoin](https://old.tcmsp-e.com/molecule.php?qn=2392) | 328.39 | 2.48 | 0 | 5 | 46.69 | 0.55 | -0.12 | 0.37 | 0.32 | 7.7 |
| MOL002393 | [Demethyldelavaine A](https://old.tcmsp-e.com/molecule.php?qn=2393) | 700.91 | -0.12 | 4 | 13 | 34.52 | -0.69 | -1.19 | 0.18 | 0.23 | 26.05 |
| MOL002394 | [Demethyldelavaine B](https://old.tcmsp-e.com/molecule.php?qn=2394) | 700.91 | -0.12 | 4 | 13 | 34.52 | -0.7 | -1.01 | 0.18 | 0.22 | 26.75 |
| MOL002395 | [Deoxyandrographolide](https://old.tcmsp-e.com/molecule.php?qn=2395) | 334.5 | 3.02 | 2 | 4 | 56.3 | 0.18 | -0.49 | 0.31 | 0.27 | 2.79 |
| MOL002397 | [karakoline](https://old.tcmsp-e.com/molecule.php?qn=2397) | 377.58 | -0.05 | 3 | 5 | 51.73 | 0.32 | -0.03 | 0.73 | 0.15 | 11.1 |
| MOL002398 | [Karanjin](https://old.tcmsp-e.com/molecule.php?qn=2398) | 292.3 | 2.94 | 0 | 4 | 69.56 | 1.22 | 0.62 | 0.34 | 0.26 | 13.15 |
| MOL002401 | [Neokadsuranic acid B](https://old.tcmsp-e.com/molecule.php?qn=2401) | 452.74 | 7.05 | 1 | 3 | 43.1 | 0.69 | -0.01 | 0.85 | 0.29 | 12.05 |
| MOL002406 | [2,7-Dideacetyl-2,7-dibenzoyl-taxayunnanine F](https://old.tcmsp-e.com/molecule.php?qn=2406) | 776.9 | 3.12 | 1 | 14 | 39.43 | -0.75 | -1.3 | 0.38 | 0.38 | 10.95 |
| MOL002410 | [benzoylnapelline](https://old.tcmsp-e.com/molecule.php?qn=2410) | 463.67 | 3.12 | 2 | 5 | 34.06 | 0.19 | -0.38 | 0.53 | 0.31 | 15.72 |
| MOL002415 | [6-Demethyldesoline](https://old.tcmsp-e.com/molecule.php?qn=2415) | 453.64 | -1.97 | 4 | 8 | 51.87 | -0.26 | -0.57 | 0.66 | 0.12 | 13.14 |
| MOL002416 | [deoxyaconitine](https://old.tcmsp-e.com/molecule.php?qn=2416) | 629.82 | 0.25 | 2 | 11 | 30.96 | -0.23 | -0.61 | 0.24 | 0.19 | 22.64 |
| MOL002419 | [(R)-Norcoclaurine](https://old.tcmsp-e.com/molecule.php?qn=2419) | 271.34 | 2.57 | 4 | 4 | 82.54 | 0.63 | 0.03 | 0.21 | 0.34 | 3.85 |
| MOL002421 | [ignavine](https://old.tcmsp-e.com/molecule.php?qn=2421) | 449.59 | 1.22 | 3 | 6 | 84.08 | -0.07 | -0.49 | 0.25 | 0.32 | 28.93 |
| MOL002422 | [isotalatizidine](https://old.tcmsp-e.com/molecule.php?qn=2422) | 407.61 | -0.73 | 3 | 6 | 50.82 | -0.11 | -0.62 | 0.73 | 0.14 | 11.59 |
| MOL002423 | [jesaconitine](https://old.tcmsp-e.com/molecule.php?qn=2423) | 675.85 | -0.99 | 3 | 13 | 33.41 | -0.44 | -1.16 | 0.19 | 0.19 | 26.06 |
| MOL002433 | [(3R,8S,9R,10R,13R,14S,17R)-3-hydroxy-4,4,9,13,14-pentamethyl-17-[(E,2R)-6-methyl-7-[(2R,3R,4S,5S,6R)-3,4,5-trihydroxy-6-[[(2R,3R,4S,5S,6R)-3,4,5-trihydroxy-6-(hydroxymethyl)oxan-2-yl]oxymethyl]oxan-2-yl]oxyhept-5-en-2-yl]-1,2,3,7,8,10,12,15,16,17-decahydr](https://old.tcmsp-e.com/molecule.php?qn=2433) | 781.1 | 2.2 | 8 | 13 | 41.52 | -2.31 | -3.18 | 0.22 | 0.25 | 10.51 |
| MOL002434 | [Carnosifloside I_qt](https://old.tcmsp-e.com/molecule.php?qn=2434) | 456.78 | 5.69 | 2 | 3 | 38.16 | 0.28 | -0.77 | 0.8 | 0.25 | 7 |
| MOL000538 | [hypaconitine](https://old.tcmsp-e.com/molecule.php?qn=538) | 615.79 | -0.1 | 2 | 11 | 31.39 | -0.34 | -0.78 | 0.26 | 0.21 | 19.87 |
| MOL000073 | [ent-Epicatechin](https://old.tcmsp-e.com/molecule.php?qn=73) | 290.29 | 1.92 | 5 | 6 | 48.96 | 0.02 | -0.64 | 0.24 | 0.34 | 0.63 |
| MOL004576 | [taxifolin](https://old.tcmsp-e.com/molecule.php?qn=4576) | 304.27 | 1.49 | 5 | 7 | 57.84 | -0.23 | -0.8 | 0.27 | 0.39 | 14.41 |
| MOL011169 | [Peroxyergosterol](https://old.tcmsp-e.com/molecule.php?qn=11169) | 428.72 | 6.73 | 1 | 3 | 44.39 | 0.86 | 0.43 | 0.82 | 0.24 | 4.06 |
